# Ribosomal DNA and Plastid Markers Used to Sample Fungal and Plant Communities from Wetland Soils Reveals Complementary Biotas

**DOI:** 10.1371/journal.pone.0142759

**Published:** 2016-01-05

**Authors:** Teresita M. Porter, Shadi Shokralla, Donald Baird, G. Brian Golding, Mehrdad Hajibabaei

**Affiliations:** 1 McMaster University, Biology Department, Hamilton, ON, L8S 4K1, Canada; 2 Biodiversity Institute of Ontario & Department of Integrative Biology, University of Guelph, Guelph, ON, N1G 2W1, Canada; 3 Environment Canada @ Canadian Rivers Institute, University of New Brunswick, Fredericton, NB, E3B 6E1, Canada; Université Paris-Sud, FRANCE

## Abstract

Though the use of metagenomic methods to sample below-ground fungal communities is common, the use of similar methods to sample plants from their underground structures is not. In this study we use high throughput sequencing of the ribulose-bisphosphate carboxylase large subunit (rbcL) plastid marker to study the plant community as well as the internal transcribed spacer and large subunit ribosomal DNA (rDNA) markers to investigate the fungal community from two wetland sites. Observed community richness and composition varied by marker. The two rDNA markers detected complementary sets of fungal taxa and total fungal composition clustered according to primer rather than by site. The composition of the most abundant plants, however, clustered according to sites as expected. We suggest that future studies consider using multiple genetic markers, ideally generated from different primer sets, to detect a more taxonomically diverse suite of taxa compared with what can be detected by any single marker alone. Conclusions drawn from the presence of even the most frequently observed taxa should be made with caution without corroborating lines of evidence.

## Introduction

Fungi are important members of ecosystem functioning and play critical roles in nutrient cycling as symbionts, saprotrophs, and pathogens [1]. Below-ground mycorrhizal fungi in particular, may physically link the roots of different plant species and help to regulate plant diversity [2–3]. When monitoring fungal and plant communities from bulk soil using DNA-based methods, actively growing fungal mycelia and plant roots are detected as well as inactive propagules such as fungal sclerotia, plant rhizomes, spores, and seeds. However, even inactive portions of the below-ground community may have important future impacts. For example, fungal pathogens can affect the composition of the plant seed bank and subsequent plant recruitment [4–5]. Additionally, fungal mutualists and saprophytes in the fungal spore bank contribute to the rapid turnover of the microbial community in soils in response to disturbance or a change in seasons [6–9].

Due to the recalcitrance of many fungi towards cultivation using standard methods, and an abundance of vegetatively growing fungi with a paucity of characters for morphology-based identification, mycologists were early adopters of PCR-based detection and DNA-based identification methods [10–12]. Many fungal metagenomic studies using standard Sanger sequencing, and now high throughput sequencing, have been conducted in a variety of environments including bulk soil such as [7, 13–15]. In contrast, PCR-based studies to monitor underground plant parts are rare [16–18]. Since plants and fungi co-exist in the same soil matrix these taxa can be studied in tandem to gain a more holistic understanding of below-ground communities in general and plant-fungal interactions in particular.

The internal transcribed spacer (ITS) region of nuclear encoded ribosomal DNA (rDNA) has been proposed as a suitable fungal barcode [19]. The ITS region is comprised of the internal transcribed spacer 1 (ITS1), 5.8S rRNA gene, and the internal transcribed spacer 2 (ITS2) with the greatest sequence variation in the ITS1 and ITS2 regions. Several studies have examined the implications of using ITS for species identification using high throughput sequencing and have found that numerous methodological biases exist [20–26]. Despite these challenges, many ITS rDNA reference sequences are available in the AFTOL (Assembling the Fungal Tree of Life), UNITE, and GenBank sequence databases and tools have been developed to facilitate the use of ITS for fungal metagenomic studies [27–31].

Large subunit (LSU) rDNA contains variable domains at the 5’ end as well as highly conserved regions at the 3’ end suitable for taxonomically diverse phylogenetic analyses as well as species- to family-level classifications. LSU rDNA reference sequences are also available through the AFTOL, UNITE, and GenBank databases. LSU rDNA is particularly heavily sampled for mushroom-forming fungi [32–33] and has been used as a 'barcoding' marker for yeasts [34–35]. Previous fungal metagenomic studies of various soils have also used this region [36–38]. Similar to studies with ITS, methodological biases also exist with the use of LSU rDNA in metagenomic studies [39].

The ribulose-bisphosphate carboxylase large subunit (rbcL) plastid gene is one of two proposed plant barcoding markers [40]. This multi-copy protein-coding gene is relatively conserved and suitable for phylogenetic studies [41] and it has been shown to resolve species in 85% or more of cases when using BLAST against GenBank sequences [42–43]. Though the rbcL marker may not be able to identify all plants to the species level on its own, it was one of the first plant barcoding markers to be used in a multigene identification approach [43]. Because the diversity of plants was expected to be quite tractable compared to fungal diversity, we only used a single marker, rbcL, to survey plant diversity. The rbcL marker is well represented in the NCBI GenBank nucleotide database.

Most metagenomic studies focusing on soil fungal communities involve the use of a single DNA marker. Because we knew that fungal diversity would likely be orders of magnitude higher than plant diversity in soil, we chose to use two fungal markers to increase our chances of detecting as much of this diversity as possible. To the best of our knowledge this is the first study to use two DNA markers (ITS + LSU) with largely fungal-specific primers as well as a plant-specific marker (rbcL) to monitor both the fungal and plant communities from the same soil samples simultaneously. We hypothesized that the fungal community detected by ITS and LSU rDNA would be largely similar, and that the use of the ITS + LSU + rbcL markers would together detect a richer assortment of organisms than any single marker. This study characterizes the reproducibility and taxonomic breath detected by these various markers and highlights areas of potential concern for future metagenomic and biomonitoring studies.

## Materials and Methods

### Field sampling

We sampled soil cores from two key wetland areas within the Peace-Athabasca Delta in Wood Buffalo National Park in northern Alberta, Canada. Field permits were granted by Parks Canada and samples were collected by Environment Canada and Parks Canada staff. The fieldwork did not involve endangered or protected species. Site A falls within Egg Lake (N 58° 54.535’ W 111° 25.398’) and site B falls within Johnny’s Cabin Pond (N 58° 29.688’ W 111° 30.773’). These deltaic wetland sites are currently threatened by industrial hydro-electric development and potential downstream oil sands contamination [44]. Physical and chemical analyses of these two samples are summarized in Table 1.

**Table 1:** Physical and chemical soil measurements.

Sample ID	Site A	Site B
Total Carbon (% dry)	36.7	0.771
Inorganic Carbon (% dry)	0.22	0.3
Organic Carbon (% dry)	36.5	0.471
Organic matter "Walkley-Black" (% dry)	63	1.1
Phosphorus (mg/L soil dry)	11	5.2
Magnesium (mg/L soil dry)	750	270
Potassium (mg/L soil dry)	170	59
Manganese (mg/L soil dry)	4.6	28
Zinc (mg/L soil dry)	12	1.1
pH	6.3	8.1
% soil moisture (% dry)	413.24	32.09
Nitrogen (% dry)	2.52	<0.05

Soil samples were collected in August 2010 using the following method: for each sampling site the top 10cm of soil was sampled at three sampling locations within the site approximately 100 to 200 meters apart. In each sampling location, three soil cores were sampled within one square meter. Each soil core was then stored in 50 ml sterile Falcon tubes. Samples were frozen on dry ice in the field and stored in a -70°C freezer until shipped to the Hajibabaei laboratory at the University of Guelph, Ontario for processing.

### Sample processing

Frozen soil core samples were homogenized and one gram of each soil core was used for total DNA extraction using a PowerSoil DNA isolation kit (cat.# 12888–100, MO BIO Laboratories, Inc., California, USA). Ten extractions (100 mg each) were done for each soil core and each extraction was eluted with 50 μL of molecular biology grade water. DNA extracts of each sample were pooled and used for further amplification. The ITS rDNA region (~ 600 bp) was targeted using the fungal specific ITS1F and ITS4 primers [45–46]. The 5'-LSU rDNA region (~ 900 bp) was targeted using the largely fungal specific LR0R_F and LR5-F primers [47]. The rbcL region (~ 600 bp) was targeted for plant identification using the primers rbcLa-F and rbcLa-R [48–49].

Marker amplification was done in a two-step PCR regime, the first PCR round was done using target specific primers (without the 454 tail). The second PCR round used the same primer sets with hybrid 454 fusion-tailed primers and specifically designed multiplex identifier (MID) tag. Each PCR contained 2 μL DNA template, 17.5 μL molecular biology grade water, 2.5 μL 10x reaction buffer, 1 μl 50x MgCl_2_ (50 mM), 0.5 μL dNTP mix (10 mM), 0.5 μL forward primer (10 mM), 0.5 μL reverse primer (10 mM), and 0.5 μL Invitrogen Platinum Taq polymerase (5 U/μL) in a total volume of 25 μL. PCR conditions were 95°C for 5 min; 15 cycles of 94°C for 40 s, (52°C for ITS, 48°C for LSU and 55°C for rbcL) for 1 min, and 72°C for 30s; and 72°C for 5 min. Amplicons were purified with Qiagen MinElute PCR purification columns and eluted in 50 μL molecular biology grade water. The purified amplicons from the first PCR round were used as template in the second PCR round using 454 fusion tailed and MID-tagged primers in a 30-cycle amplification regime. An Eppendorf Mastercycler ep gradient S thermal cycler was used for all PCR reactions. Negative controls were included in all experiments.

### 454 Pyrosequencing

The three indexed markers amplified from each soil core were purified and fluorometrically quantified. Equimolar amounts of the MID-generated amplicons were combined and sequenced on the 454 Genome Sequencer FLX System (Roche Diagnostics) following the amplicon sequencing protocol with GS Titanium chemistry. Amplicons of each soil core were bidirectionally sequenced in 2 (1/16) regions of a full sequencing run (70 x 75 pico titer plate). Further details of the 454 pyrosequencing run are available by request from the corresponding author. Raw sequence data is available through the NCBI SRA: SRP066030

### Bioinformatic methods

A semi-automated Perl pipeline was created. Raw reads were sorted by primer sequences for the ITS, LSU, and rbcL markers using AGREP version 2.04 allowing 1 mismatch. Sorted reads were quality-trimmed using SeqTrim [50] with a 10 bp sliding window, excluding windows with an average Phred score less than 20, and removing reads less than 80 bp after trimming.

Quality-trimmed reads were sorted by average read quality then clustered into operational taxonomic units (OTUs) with USEARCH version 4.0.43 [51]. Clustering reads into OTUs allowed us to retain many sequence types representing an array of taxonomic groups, while absorbing some of the diversity represented by intraspecific variation. Rare OTUs comprised of only one or two reads (singletons and doubletons) were excluded from downstream analyses to avoid analyzing diversity generated by sequencing error [52–54]. These precautions allowed us to dereplicate our dataset, account for potential chimeras and other sequencing artefacts, and facilitate downstream analyses by being conservative with our inclusion of rare sequence types. A variety of sequence similarity cutoffs were tested (S1 Text, S2 Fig) and we ultimately used a 97% sequence similarity cutoff to delimit OTUs for the ITS marker and for the 5’ LSU, and a 95% similarity cutoff for the 3’ LSU and rbcL marker. The 5’ and 3’ sequence reads were initially analyzed separately to avoid any possible double-counting of the same PCR-template that might inflate richness values. OTUs generated by USEARCH were reformatted using custom Perl scripts so that statistical analyses could be run with MOTHUR v.1.15.0 [55]. OTU classifications were carried out using BLAST (blastall version 2.2.15) against a local installation of the ‘nt’ GenBank database [December 10, 2010] using default parameters with 8 processors per job [56]. To minimize the number of incorrect annotations based on a best BLAST hit approach, this was followed by lowest common ancestor (LCA) parsing using MEGAN version 4.40.6 [23, 57]. We used the following LCA filter settings: minimum support = 1, minimum score = 100, top percent = 1%, win score = 0.0.

Taxonomic comparisons were also performed using MEGAN using three ecological indices. The ITS, LSU, and rbcL datasets were normalized, MEGAN classifications were summarized to the order level, and comparisons were visualized using multi-dimensional scaling plots. The Bray-Curtis dissimilarity statistic measures the number of species unique to either of two sites divided by the total number of species in both sites where each species is equally-weighted [58]. The non-parametric Goodall similarity index, however, gives more weight to differences between rare taxa [59]. This method has been found to be particularly appropriate for comparing microbial metagenomic datasets characterized by large numbers of rare taxa [60]. The UniFrac measure emphasizes the amount of branch length unique to either of two sites compared with the amount of branch length shared by both sites in a phylogeny. This is interpreted as representing evolution among lineages unique to each site that may reflect adaption to a specific environment [61]. MEGAN calculates a simplified UniFrac distance metric based on the NCBI taxonomy.

Differences among the number of observed OTUs from the ITS and LSU datasets from both sites were compared using MEGAN. The directed homogeneity ‘up’ test was used on normalized data with the Bonferroni correction for multiple comparisons.

## Results

### Sampling consistency and effort

The number and average length of reads and OTUs that were sorted, filtered, and clustered for each marker is shown S1 Table. The average number of OTUs sampled from three replicate libraries was relatively similar within each primer and site combination (Table 2). Additionally, the amount of OTU overlap recovered among three replicate soil samples was high, with relatively few OTUs unique to each replicate (Table 3). These replicates were combined in subsequent analyses.

**Table 2:** Average OTU richness from three soil sample replicates.

	5’ primer		3’ primer	
Marker	Site A	Site B	Site A	Site B
ITS	1138 ± 14	1034 ± 39	590 ± 8	583 ± 10
LSU	242 ± 3	325 ± 6	320 ± 2	292 ± 9
rbcL	76 ± 3	32 ± 2	92 ± 2	54 ± 1

**Table 3:** Proportion (number of OTUs excluding singletons and doubletons) of overlap from three soil sample replicates.

	5’ primer		3’ primer	
Marker	Site A	Site B	Site A	Site B
**OTUs present in three replicates**				
ITS	69% (890)	68% (799)	68% (461)	68% (452)
LSU	63% (177)	63% (238)	76% (266)	72% (233)
rbcL	88% (70)	74% (26)	77% (78)	83% (48)
**OTUs present in any two replicates**				
ITS	28% (354)	28% (328)	26% (175)	27% (181)
LSU	32% (91)	32% (122)	22% (78)	26% (85)
rbcL	11% (9)	23% (8)	20% (20)	16% (9)
**OTUs unique to a single replicate**				
ITS	3% (38)	3% (40)	5% (37)	5% (31)
LSU	5% (14)	5% (17)	2% (7)	2% (7)
rbcL	1% (1)	3% (1)	3% (3)	2% (1)

**Fig 1:**
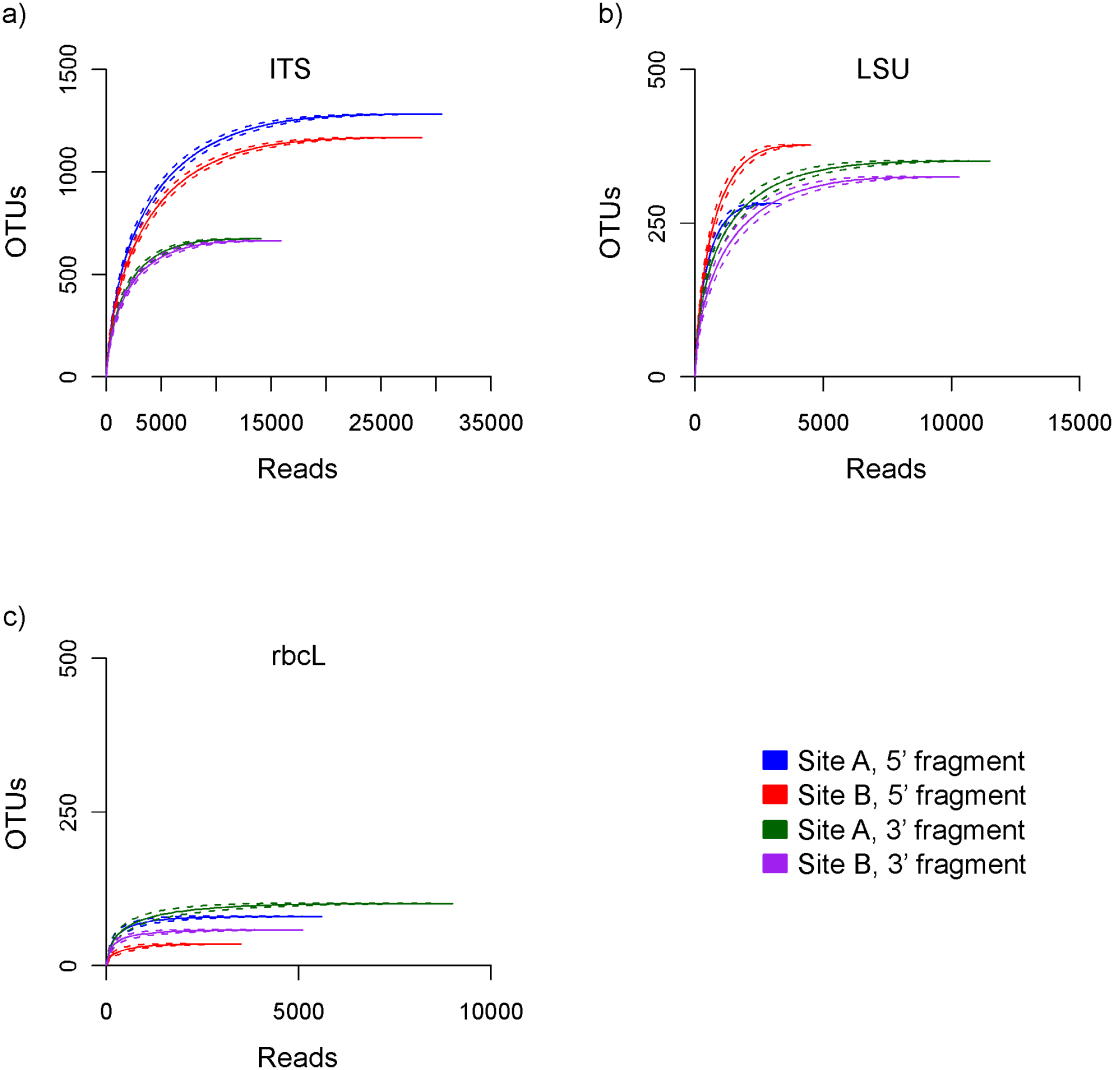
Rarefaction curves. Data are shown for 5’ and 3’ fragments sampled from two sites (A and B) for three loci: (a) ITS, (b) LSU, and (c) rbcL.

We assessed sampling effort by plotting rarefaction curves. We did not rarefy to a standard number of reads because we wanted to see how each marker performed individually without subsampling the data. The number of detected OTUs can still be fairly compared across markers in our plotted curves for any standard number of reads less than or equal to the smallest library size. For each primer and site combination, curves reach a plateau indicating sampling saturation (Fig 1). We assessed the presence of the same OTUs between both sites for each primer by plotting their read frequency distribution (S3 Fig). For each primer, we observed a few OTUs represented by many reads, and many OTUs represented by only a few reads each. For each OTU, a different number of reads were detected from each site. These data, however, are not necessarily quantitative [47].

### Taxonomic classifications

It has been previously observed that the LCA algorithm used in MEGAN may classify reads to high level taxonomic ranks and may not classify all sequences [23, 62]. The proportion of reads that could be classified to any taxonomic rank by MEGAN is shown in Table 4. MEGAN classified 94% (5113) of ITS OTUs, 97% (1658) of LSU OTUs, and 86% (529) of rbcL OTUs. We also assessed the number of OTUs assigned to various taxonomic ranks and the number of categories present at each rank for each marker (S4 Fig). Although MEGAN was able to classify nearly all our OTUs, the number of OTUs classified to the species-level represents only a fraction of the OTUs classified to more inclusive taxonomic ranks, particularly for ITS and LSU. At the species and genus levels, ITS detected more categories than LSU, however, from the family to kingdom levels the number of detected categories is similar for both markers. Overall, more OTUs are detected by ITS and LSU than for rbcL, indicating a generally high fungi to plant ratio similar to that observed from other studies [63]. With MEGAN results summarized to the genus level, we were also able to directly compare the number of taxonomic categories recovered from each marker (Table 5). The number of categories detected by any single marker was less than any two-marker combination and three markers combined detected the greatest number of categories.

**Table 4:** MEGAN classification summary (to any taxonomic rank).

		MEGAN		
Marker	Total OTUs	Assigned	Unassigned	No hits
ITS	5454	94% (5113)	2% (98)	4% (243)
LSU	1710	97% (1658)	0% (6)	3% (46)
rbcL	612	86% (529)	0% (0)	14% (83)

**Table 5:** Number of MEGAN categories at the genus rank.

	5' primer		3' primer	
Marker	Site A	Site B	Site A	Site B
**Single markers**				
ITS	216	182	138	130
LSU	89	100	113	113
rbcL	20	13	22	19
**Two-marker combinations**				
ITS+LSU	268	244	212	205
ITS+rbcL	236	195	160	149
LSU+rbcL	109	113	135	131
**Three markers**				
ITS+LSU+rbcL	288	257	234	224

### Dataset comparisons

**Fig 2:**
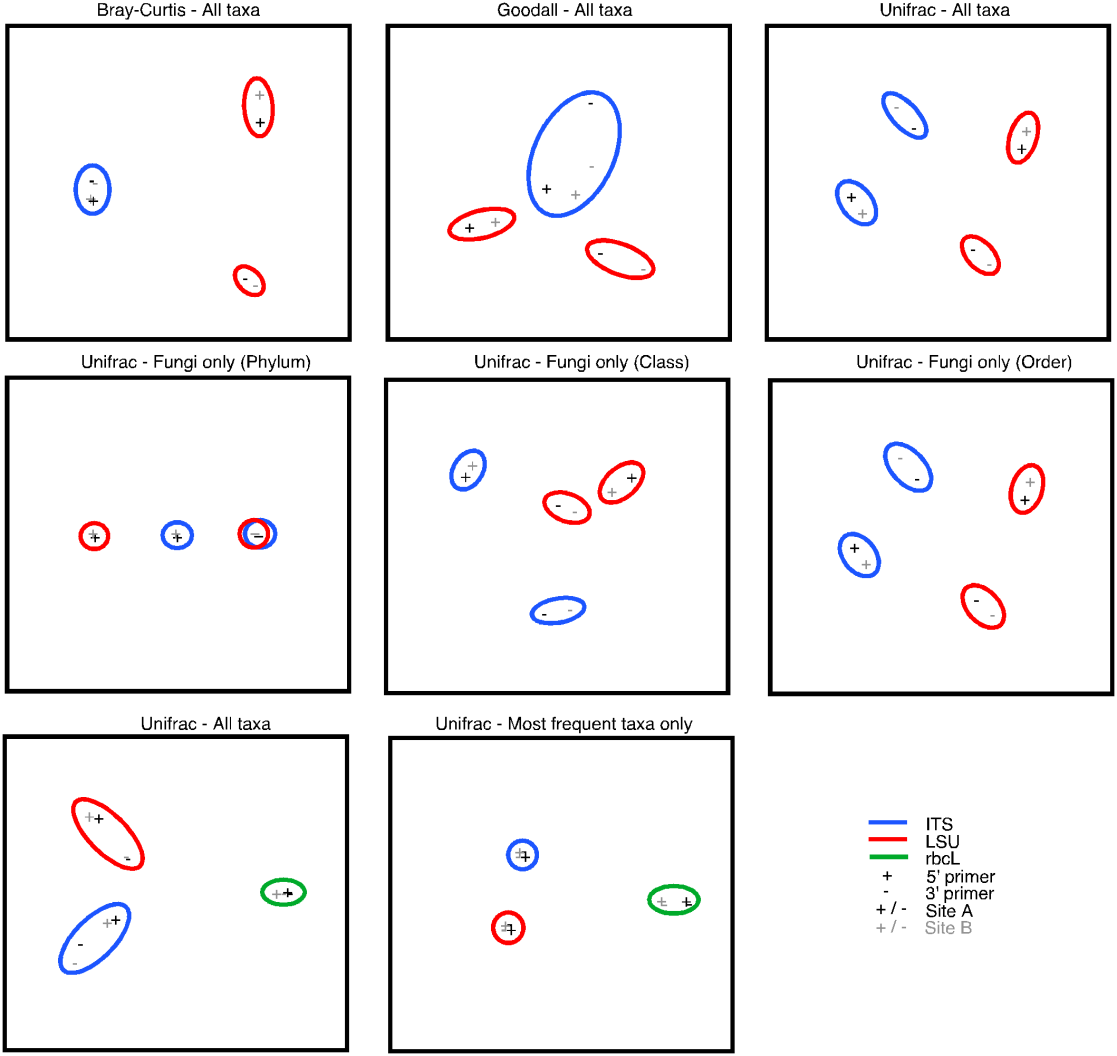
Comparison of the taxonomic content among the metagenomic datasets. Normalized reads were used to compare datasets in MEGAN for a variety of ecological indices including the Bray-Curtis metric, Goodall ecological index, and a simplified Unifrac metric. Each marker is indicated by circled points: ITS (blue), LSU (red), and rbcL (green). Datasets generated using the forward 5’ primer (+) or the reverse 3’ primer (-) from two sites A (black) and B (grey) are shown.

A comparison of the taxonomic content of the datasets using several different ecological indices in MEGAN is shown in Fig 2. When ITS and LSU taxa are summarized at the order level, three of the ecological indices give different cluster patterns depending on what component of diversity is emphasized by each measure. Giving more weight to distances among rare taxa with the Goodall index emphasizes differences in the taxonomic composition of the ITS datasets compared with the Bray-Curtis statistic where each taxon is equally-weighted. With the UniFrac metric ITS and LSU datasets cluster by primer emphasizing the presence of unique taxonomic lineages. When the ITS and LSU datasets are summarized at increasingly more exclusive taxonomic levels from phylum to order, the clustering pattern breaks down such that eventually each dataset clusters mainly by primer. When the UniFrac metric is used with the ITS + LSU + rbcL datasets, points cluster by marker with sub-clustering by primer for the ITS and LSU datasets. When only the most frequently observed taxa are considered, datasets cluster mainly by marker without any sub-clustering by primer. For the rbcL dataset, where only the most frequently observed taxa were analyzed, datasets show some sub-clustering by site.

**Fig 3:**
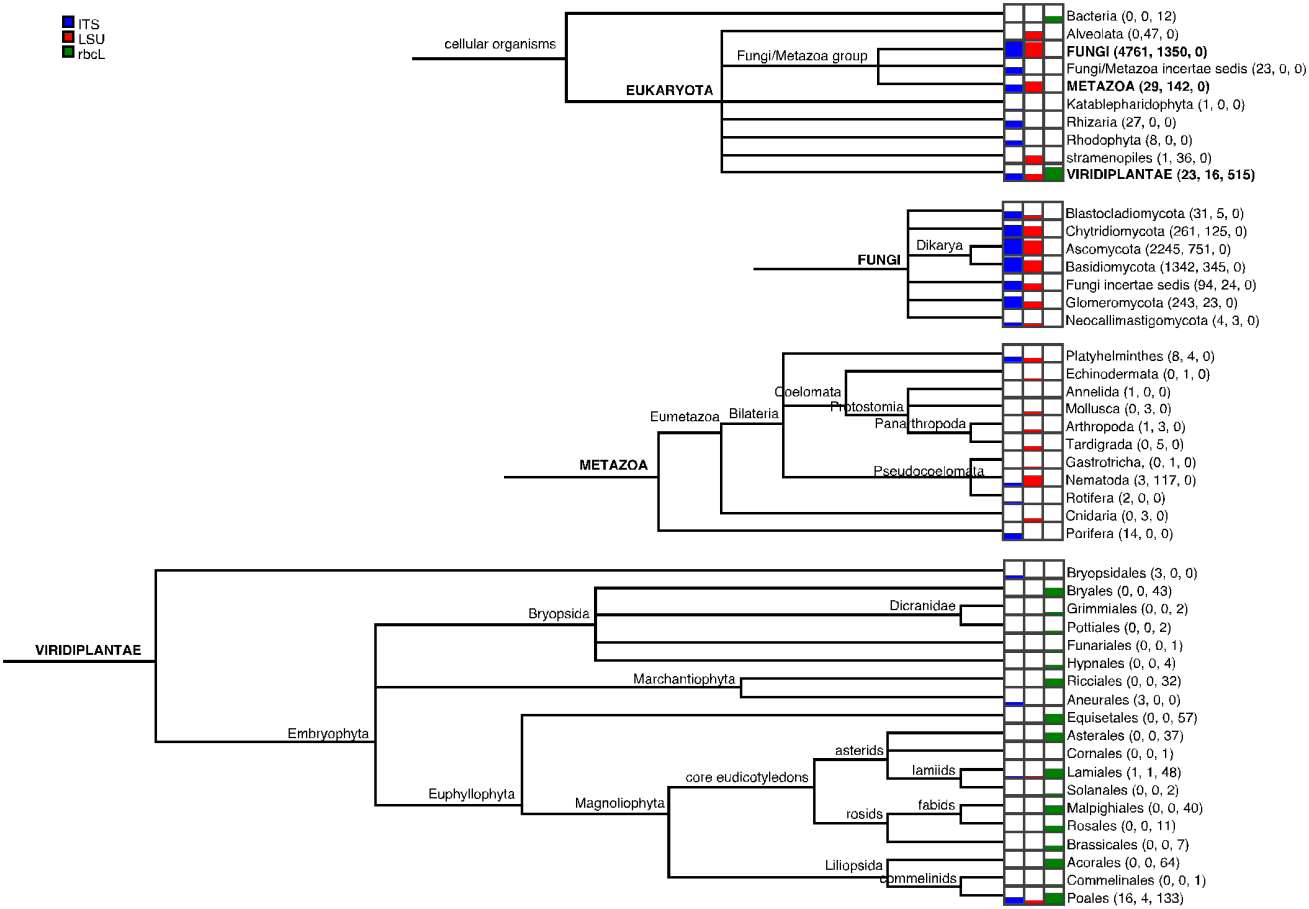
Taxonomic distribution of MEGAN-classified OTUs. Taxonomic distributions are summarized for the Eukaryota at the Kingdom rank, for the Fungi at the phylum rank, for the Metazoa at the phylum rank, and for the Viridiplantae at the order rank. Each dataset (columns) shows meters representing the absolute number of reads classified to various taxonomic ranks (rows/leaves).

A summary of the OTUs from each marker and classified by MEGAN is shown in Fig 3. A detailed breakdown of classifications is available in S2 and S3 Tables. Only the ITS primers recovered OTUs classified as Fungi/Metazoa *incertae sedis*, Katablepharidiophyta (heterotrophic flagellates), Rhizaria (unicellular eukaryotes, protists, amoeboids, flagellates), and Rhodophyta (red algae). Only the LSU primers recovered OTUs classified as Alveolata (mostly single celled eukaryotes, protists, protozoa, flagellates) and stramenopiles (mostly algae and filamentous Oomycetes). Only the rbcL primers recovered OTUs classified as Bacteria. This last likely represents the presence of RuBisCO or RuBisCO-like proteins in these Bacteria [41, 64]. The ITS + LSU markers both recovered OTUs classified as Fungi (yeasts, moulds, mushrooms) and Metazoa (multicellular eukaryotes, animals). The ITS + LSU + rbcL markers each recovered OTUs classified as Viridiplantae (plants), though the greatest number and diversity of plants by far was detected with rbcL.

The top ten most frequently sampled MEGAN categories summarized at the order rank are shown in Table 6. With ITS, only fungal orders are most frequently sampled. With LSU, both fungi and nematodes are frequently sampled. The communities retrieved using the ITS and LSU markers were not found to differ among sites and the taxa listed here were present in both sites. For many categories, the number of OTUs detected by the ITS and LSU marker are significantly different. Five of the most frequently observed orders detected by both ITS and LSU are the Pezizales (moulds, morels, and cup fungi), Helotiales, Pleosporales, Agaricales (mushroom-forming fungi), and Hypocreales. The mitosporic Ascomycota category, frequently sampled by the ITS and LSU markers, includes a heterogeneous group of asexual Ascomycota fungi for which a sexual stage is unknown or does not exist. These groups represent an array of saprotrophic, mycorrhizal, pathogenic, endophytic, and lichen-forming taxa. Taxa from the Capnodiales, Glomerales (arbuscular mycorrhizal), Thelphorales (ectomycorrhizal), and Tremellales (jelly fungi and yeasts) were most frequently found with ITS. Taxa from the Tylenchida and Rhabditida (nematodes), Polyporales (bracket fungi), Sordariales (saptrotrophic fungi), Platygloeales (saprotrophic and plant parasitic fungi), and Chytridiales (aquatic fungi with flagellated zoospores) were most frequently sampled with LSU. Although site A was much more wet than site B, the number of OTUs of aquatic fungi in the Chytridiales was similar. Among the most frequently observed rbcL OTUs, are orders of plants expected to be found in wet habitats, especially site A, such as the ubiquitous Poales (grasses and sedges), the Acorales (grass-like evergreen plants), as well as the spore-dispersed Equisetales (horsetails) and Bryales (mosses).

**Table 6:** The most frequent MEGAN categories at the order rank for each marker.

MEGAN Node / Order^a^	Number of OTUs (Site A, Site B)	
	**ITS**	**LSU**
Pezizales (Fungi, Ascomycota)*	330 (168, 162)	98 (44, 54)
Helotiales (Fungi, Ascomycota)*	259 (132, 127)	80 (34, 46)
Pleosporales (Fungi, Ascomycota)*	198 (97, 101)	42 (17, 25)
Agaricales (Fungi, Basidiomycota)	194 (100, 94)	37 (17, 20)
Hypocreales (Fungi, Ascomycota)	157 (78, 79)	66 (31, 35)
Capnodiales (Fungi, Ascomycota)*	121 (61, 60)	22 (12, 10)
Glomerales (Fungi, Glomeromycota)*	116 (61, 55)	3 (2, 1)
Mitosporic Ascomycota (Fungi, Ascomycota)*	114 (57, 57)	32 (14, 18)
Thelphorales (Fungi, Basidiomycota)*	111 (55, 56)	15 (7, 8)
Tremellales (Fungi, Basidiomycota)*	106 (58, 48)	21 (10, 11)
	**LSU**	**ITS**
Pezizales (Fungi, Ascomycota)*	98 (44, 54)	330 (168, 162)
Helotiales (Fungi, Ascomycota)*	80 (34, 46)	259 (132, 127)
Hypocreales (Fungi, Ascomycota)	66 (31, 35)	157 (78, 79)
Tylenchida (Metazoa, Nematoda)*	57 (25, 32)	0 (0, 0)
Polyporales (Fungi, Basidiomycota)*	45 (19, 26)	82 (41, 41)
Pleosporales (Fungi, Ascomycota)*	42 (17, 25)	198 (97, 101)
Agaricales (Fungi, Basidiomycota)	37 (17, 20)	194 (100, 94)
Rhabditida (Metazoa, Nematoda)*	37 (17, 20)	0 (0, 0)
Sordariales (Fungi, Ascomycota)*	34 (17, 17)	81 (30, 51)
Mitosporic Ascomycota (Fungi, Ascomycota)*	32 (14, 18)	114 (57, 57)
Platygloeales (Fungi, Basidiomycota)*	32 (19, 13)	46 (25, 21)
Chytridiales (Fungi, Chytridiomycota)*	24 (12, 12)	75 (36, 39)
	**rbcL**	
Poales (Viridiplantae, Streptophyta)	133 (107, 26)	
Acorales (Viridiplantae, Streptophyta)	64 (27, 37)	
Equisetales (Viridiplantae, Streptophyta)	57 (30, 27)	
Lamiales (Viridiplantae, Streptophyta)	48 (30, 18)	
Bryales (Viridiplantae, Streptophyta)	43 (33, 10)	
Malpighiales (Viridiplantae, Streptophyta)	40 (21, 19)	
Asterales (Viridiplantae, Streptophyta)	37 (23, 14)	
Ricciales (Viridiplantae, Streptophyta)	32 (20, 12)	
Rosales (Viridiplantae, Streptophyta)	11 (11, 0)	
Brassicales (Viridiplantae, Streptophyta)	7 (7, 0)	

^a^Results from the ITS and LSU markers are compared at each MEGAN node using the directed homogeneity ‘up’ test performed in MEGAN using normalized datasets with Bonferroni-corrected comparisons.

Significant differences in the number of observed ITS and LSU OTUs are indicated by an asterisk (*).

The top ten most frequently sampled OTUs summarized to species rank by MEGAN are shown in S4 Table. A mixture of fungi comprised of mycorrhizal symbionts, saprotrophs, and parasitic species; as well as plants known to be mycorrhizal and expected to be abundant in a wetland habitat, appear among the most frequently observed OTUs. Additionally, two nematode species, one alveolate, and one stramenopile species were identified. Only two MEGAN classified species of fungi, *Peziza badia* (cup fungus) and *Pterula echo* (coral fungus), were frequently detected by both ITS and LSU. With rbcL, we were only able to classify some of the most abundant taxa to the genus level using MEGAN. The genus *Typha*, bulrushes or cattails, was the most frequently sampled rbcL OTU. The genus *Acorus*, grasslike evergreen plants, was the second most frequently sampled rbcL OTU.

### Site A versus Site B

Though the ITS and LSU markers did not distinguish between the two wetland sites, the rbcL marker did. rbcL OTU richness differed significantly between sites. On average 76–92 rbcL OTUs were detected from Site A and 32–54 rbcL OTUs were detected from Site B. Additionally, the taxonomical composition of rbcL OTUs differed among sites. Site A is characterized by the presence of bacterial rbcL OTUs classified in the Chromatiales, Caulobacteriales, and Rhizobiales. Among the most frequently sampled rbcL OTUs, only the Rosales and Brassicales were detected from site A. Site B is characterized by the presence of mosses in the Grimmiales (moss that grows on rocks) and Pottiales.

## Discussion

### Marker specificity

Whole genome shotgun metagenomic approaches can utilize data from an array of markers selected *a posteriori* to track taxonomic groups of taxa. Using this approach previous work in the literature was able to track genus- to phylum-level Bacterial groups using six markers [65]. Though this method avoids the use of potentially biased primer-based amplification, it generates data from many loci that lack reference databases to allow species level identification. The alternative approach is to select markers *a priori* based on the availability of existing reference databases. Although the ability to link data from multiple markers to specific individuals is often lost using metagenomic methods, the data can be used to provide corroborating evidence for species presence and prevalence as we have done here.

We hypothesized that the ITS and LSU rDNA markers would recover similar sets of fungal taxa and that the ITS + LSU + rbcL markers together would recover a richer assortment of taxa than any marker on its own. We produced thousands of OTUs from the ITS, LSU, and rbcL markers that we directly compared showing a significant “rare biosphere” [66]. We observed some similarity between the ITS and LSU datasets when taxa were compared at the most inclusive taxonomic levels, however, this similarity breaks down at more specific taxonomic levels even among the most frequently observed taxa. Each marker detected a taxonomically distinct community that varied more by primer than by site, particularly for the rDNA markers. To date, only a single fungal study that we are aware of has used more than one rDNA region, SSU and ITS, to survey hundreds of fungal sequence types from bulk soil using Sanger sequencing [13]. A previous fungal study also showed that using alternative primers can affect the recovered richness and community composition of root tips that were sequenced both individually and from a pooled sample [52]. Our study supports their assertion and shows how community richness, overall taxonomic composition, and even the presence of the most frequently encountered taxa may differ according to the primer and marker used for monitoring. Recent studies in arthropods have shown support for multiple primer and multiple gene frameworks [67–68].

### Classification complexities

How can we explain our inability to detect differences among sites using the ITS and LSU markers? First, fungi are significantly more diverse than plants and our fungal sampling was not exhaustive. Despite sequencing three soil sample replicates and producing saturated rarefaction curves, the use of additional primer sets for each marker would likely recover additional taxa [69]. Second, previous work has shown that partial sequences from the 5’ and 3’ ends of the ITS region may BLAST to different species despite coming from the same full length sequence. This type of BLAST result is often used to diagnose putative chimeras in full length ITS sequences [70, 71]. Using a dataset of fungal environmental sequences previous work in the literature showed that 40% of partial ITS1 and ITS2 sequences from the same full length query may BLAST to different species [22]. Using a well-annotated fungal ITS dataset generated from individual PCRs, it was shown that partial sequences from the 5’ and 3’ ends of the same parent sequence had best BLAST matches to the correct species as well as to an incorrect species in 6% of cases for 400 bp fragments and in 15% of cases for 50 bp fragments [23]. These BLAST results may be best explained by lack of resolution among partial length ITS fragments, insufficient database coverage, or incorrectly annotated database sequences. The consequence of these observations is that taxonomic diversity recovered by the short fragments using different primers in our study may be inflated. Third, intragenomic variation among multicopy rDNA regions means that relaxed concerted evolution may result in sequences that are divergent from the consensus or barcode sequence for a species [72–74]. This type of variation can be detected from individuals by cloning and sequencing or from bulk soil DNA amplified with mixed-template PCR [75]. As a consequence, there is poor database representation for these rare alleles, and this may result in spurious BLAST matches to incorrect taxa. Fourth, the number of named fungal ITS sequences in GenBank available as references to identify new environmental sequences is greatly exceeded by the number of unnamed environmental sequences [76]. To improve the utility of reference databases, there has been a plea for increasing the sequencing of type cultures and specimens as well as for the formal classification of environmental sequences [76–78]. Progress towards automated sequence-based identification of fungal ITS sequences has been made [79–80]. As the representation in reference databases increases, so too will our ability to correctly classify taxa.

### Suggestions for future biomonitoring efforts

It is possible that next-generation sequencing platforms producing longer paired-end reads up to 600 bp may be able to produce full length ITS sequences for most fungi to circumvent the problem of working with partial ITS reads. The use of paired-end approaches would also allow forward and reverse reads to be assembled, providing an additional level of quality assurance [81]. In contrast to the rDNA markers, the rbcL marker did not show strong clustering by primer, though species level identifications using MEGAN were not always possible due to the conserved nature of this gene region. As such, it may be appropriate to use a second marker such as matK to track below-ground plant structures and to corroborate rbcL results.

The general rules for setting up mixed-template PCRs that detect the greatest sample diversity, particularly with 16S rDNA, have been known for some time and include using a low PCR cycle number, longer elongation times, and pooling multiple PCR reactions [82–84]. It is clear now that the use of multiple markers and even multiple amplicons for each marker, generated using different primers, may also be a good way to address the issue of primer bias and detect the broadest range of taxa from an environmental sample [69]. We suggest that future studies consider these parameters carefully since the high throughput nature of next-generation sequencing exaggerates these effects and even brute force sequencing will not detect maximum diversity if the primers and PCR conditions do not facilitate this. In conclusion, high throughput sequencing with multiple markers to study fungal and plant communities will be important for biomonitoring efforts such as in the Alberta oil sands.

## Supporting Information

S1 Fig Characterizing the ITS1/ITS2 component of our ITS reads using the Fungal ITS Extractor. After quality trimming and pooling all of our ITS reads, the proportion of reads in the following categories are shown in (a) ITS1 and ITS2 regions were both detected (blue); only the ITS1 region was detected (red); only the ITS2 region was detected (green); and neither the ITS1 nor the ITS2 region was detected (purple). The number of reads of various lengths is shown in (b) for the ITS1 region (red) and the ITS2 region (blue).
(PDF)

S2 Fig Effect of sequence similarity cutoffs on the number of clustered OTUs. In (a), sequence similarity cutoff values used with USEARCH are shown on the x-axis and the relative number of recovered OTUs, with respect to the total number of OTUs recovered at 100% sequence similarity, is shown on the y-axis. The following series are shown: the ITS region (5’—blue, 3’–red), the LSU region (5’—green, 3’—purple), and the rbcL region (5’–teal, 3’—orange). In (b), the increasing proportion of clustered OTUs is shown with increasing sequence similarity cutoffs. Sequence similarity increases of 1% intervals are shown on the x-axis. The resulting increase in the proportion of OTUs is shown on the y-axis using a log_2_ scale. A coverline at 20% OTU increase is shown as a black dashed line.
(PDF)

S3 Fig Frequency distributions comparing OTU recovery consistency among sites. Individual OTUs were plotted in rank order based on abundance at site A. Data are shown for three loci (ITS, LSU, and rbcL) from 5’ and 3’ fragments. Blue represents site A and red represents site B.
(PDF)

S4 Fig Distribution and richness of MEGAN-classified OTUs at each rank. Data are shown for three loci (ITS, LSU, rbcL) from 5’ and 3’ primers from two sites (A and B) combined: (a) distribution of classified OTUs across ranks and (b) richness at each rank.
(PDF)

S5 Fig Neighbor joining analysis of seed sequences classified as *Pterula echo*. ITS1 analysis used 21 taxa, including four reference sequences from GenBank, and 279 aligned characters. ITS2 analysis used 16 taxa, including four reference sequences, and 242 aligned characters. Neighbor joining analysis used the Kimura two parameter model. 1000 neighbor joining bootstrap (NJB) replicates were conducted and clades supported by greater than 60% NJB are labeled at the nodes.
(PDF)

S1 Table Raw read statistics for each library after sorting by primer sequence. (DOCX)

S2 Table Number of OTUs from order-level MEGAN classifications using GenBank taxonomy. (DOCX)

S3 Table Number of OTUs from species-level MEGAN classifications using GenBank taxonomy. (DOCX)

S4 Table The most frequent ITS, LSU, and rbcL categories summarized by MEGAN at the species level. (DOCX)

S1 Text Supporting methods and results. (DOCX)
