# Supplementary figures and images for: Ribosomal DNA and Plastid Markers Used to Sample Fungal and Plant Communities from Wetland Soils Reveals Complementary Biotas
Source: PLoS One. 2016 Jan 5;11(1):e0142759. doi: 10.1371/journal.pone.0142759 (PMC4712138; doi:10.1371/journal.pone.0142759)

a)

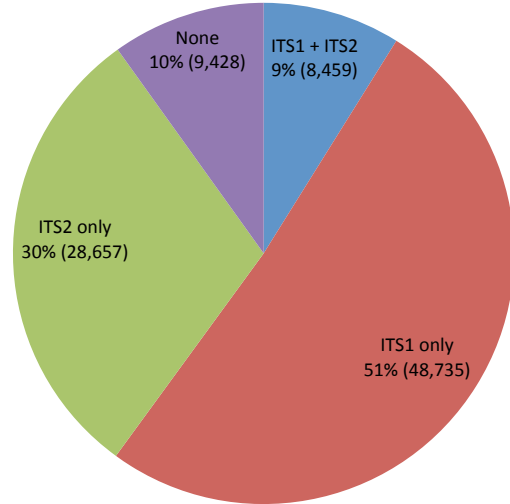

b)

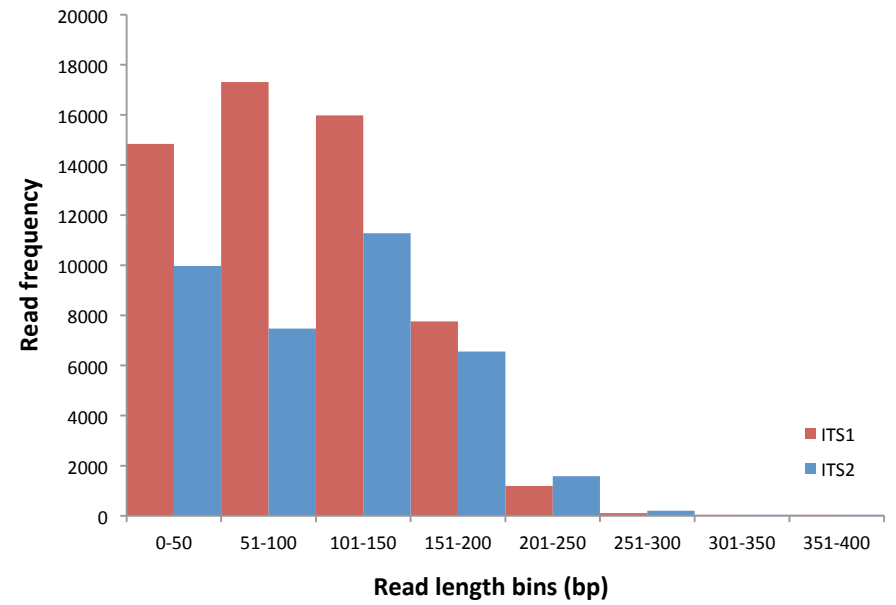

Supplement: S1 Fig — After quality trimming and pooling all of our ITS reads, the proportion of reads in the following categories are shown in (a) ITS1 and ITS2 regions were both detected (blue); only the ITS1 region was detected (red); only the ITS2 region was detected (green); and neither the ITS1 nor the ITS2 region was detected (purple). The number of reads of various lengths is shown in (b) for the ITS1 region (red) and the ITS2 region (blue). (PDF) [file pone.0142759.s001.pdf]

a)

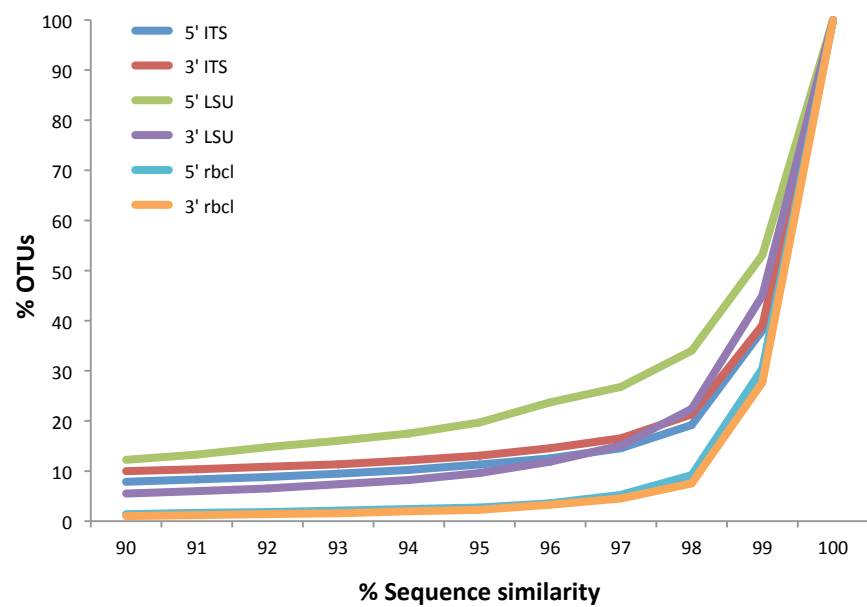

b)

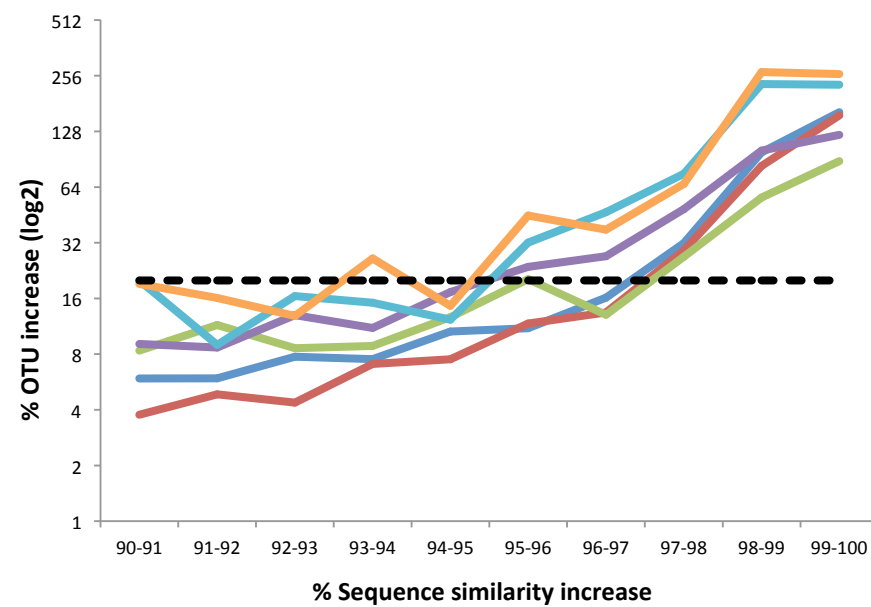

Supplement: S2 Fig — In (a), sequence similarity cutoff values used with USEARCH are shown on the x-axis and the relative number of recovered OTUs, with respect to the total number of OTUs recovered at 100% sequence similarity, is shown on the y-axis. The following series are shown: the ITS region (5’—blue, 3’–red), the LSU region (5’—green, 3’—purple), and the rbcL region (5’–teal, 3’—orange). In (b), the increasing proportion of clustered OTUs is shown with increasing sequence similarity cutoffs. Sequence similarity increases of 1% intervals are shown on the x-axis. The resulting increase in the proportion of OTUs is shown on the y-axis using a log2 scale. A coverline at 20% OTU increase is shown as a black dashed line. (PDF) [file pone.0142759.s002.pdf]

ITS

LSU

rbcl

5' OTUs

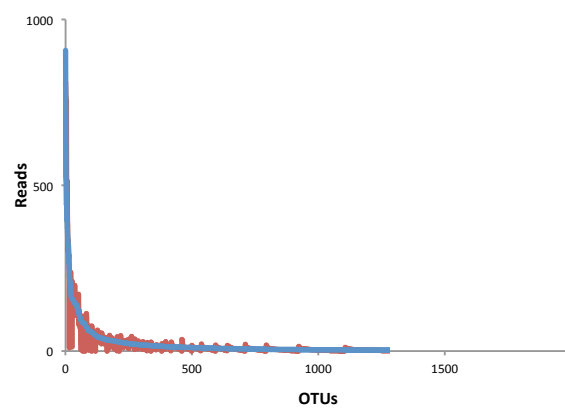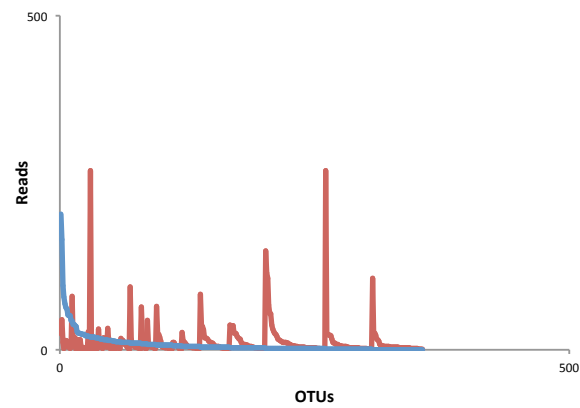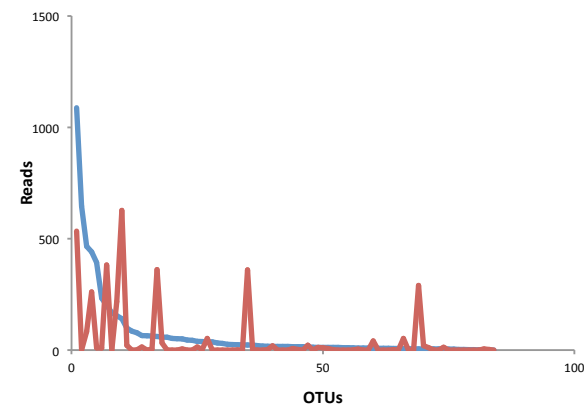

3' OTUs

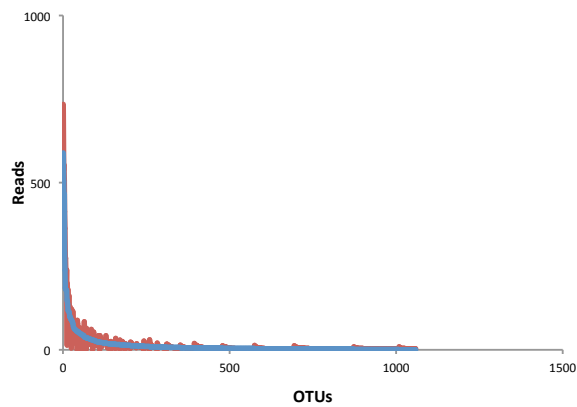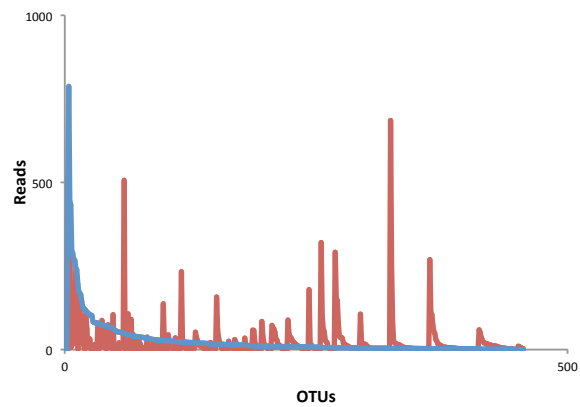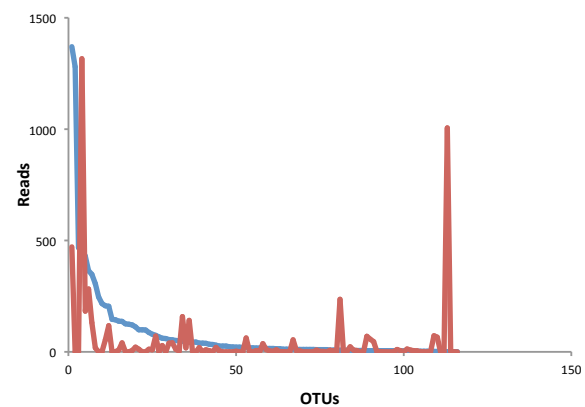

Supplement: S3 Fig — Individual OTUs were plotted in rank order based on abundance at site A. Data are shown for three loci (ITS, LSU, and rbcL) from 5’ and 3’ fragments. Blue represents site A and red represents site B. (PDF) [file pone.0142759.s003.pdf]

a)

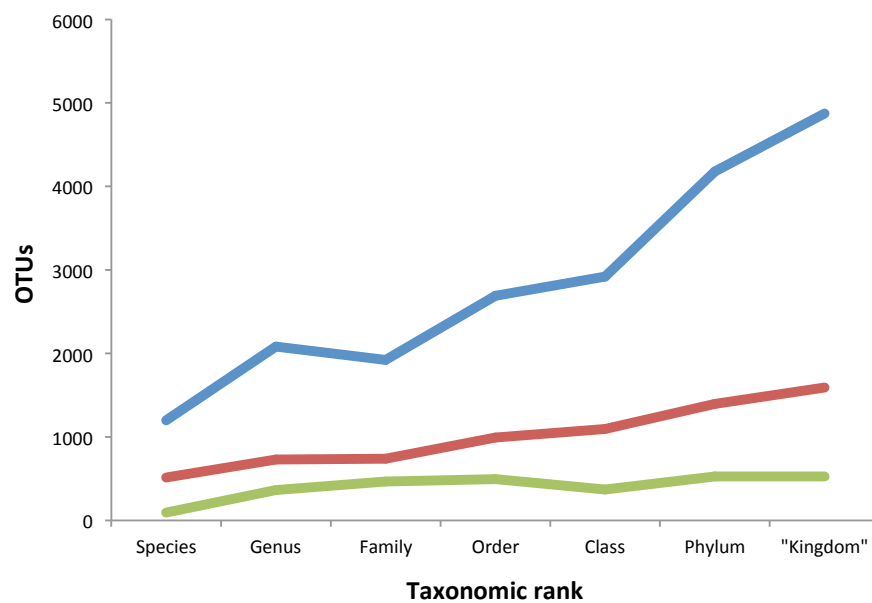

b)

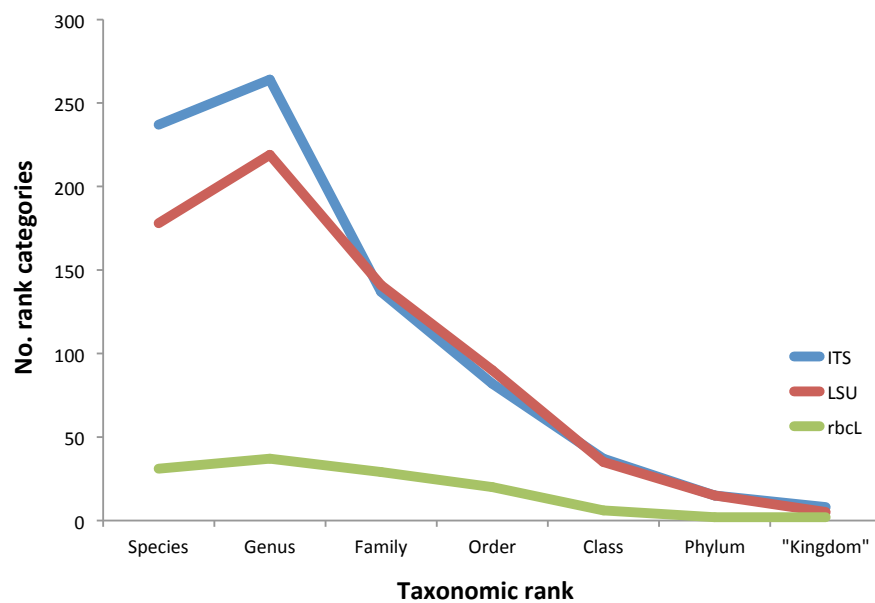

Supplement: S4 Fig — Data are shown for three loci (ITS, LSU, rbcL) from 5’ and 3’ primers from two sites (A and B) combined: (a) distribution of classified OTUs across ranks and (b) richness at each rank. (PDF) [file pone.0142759.s004.pdf]

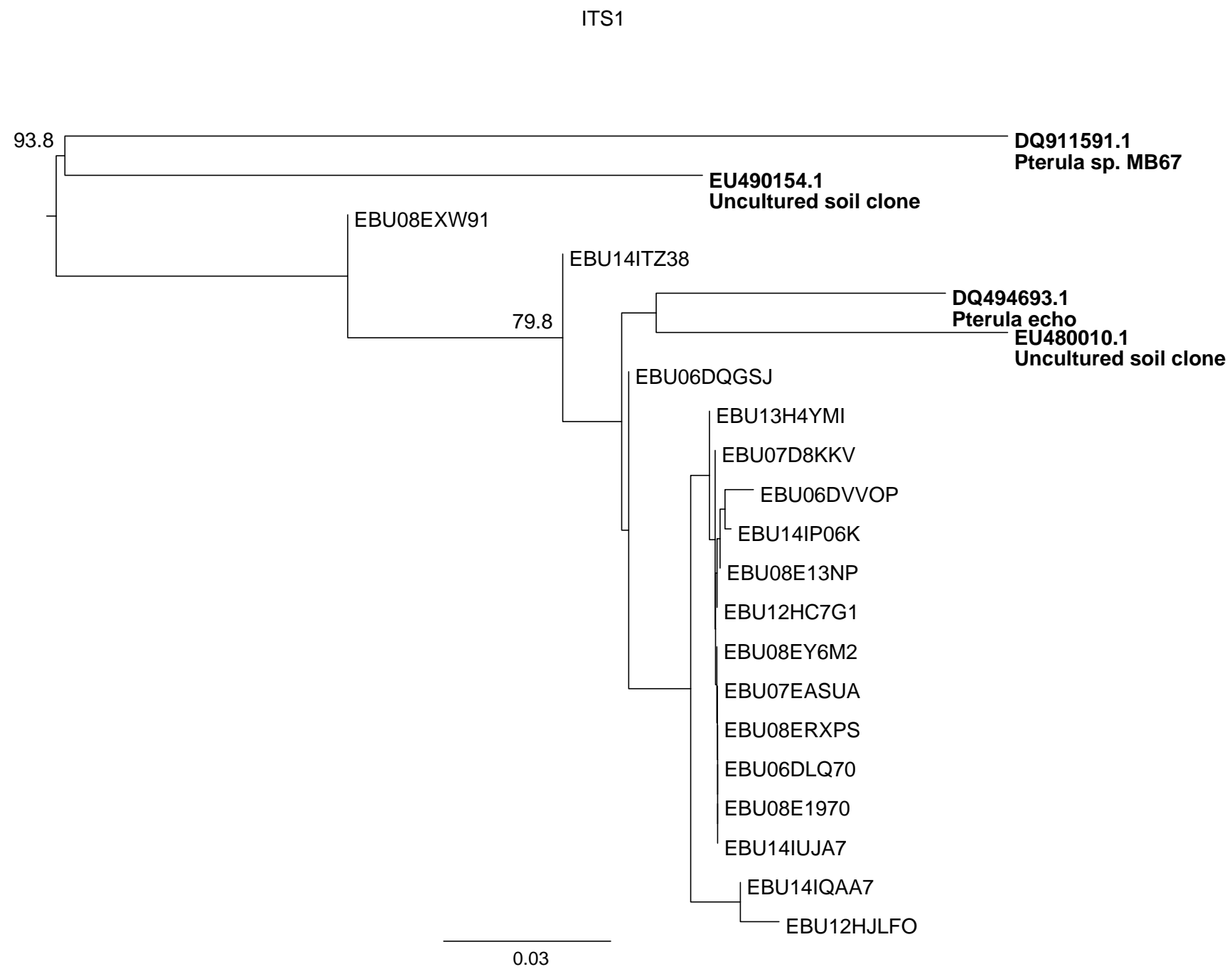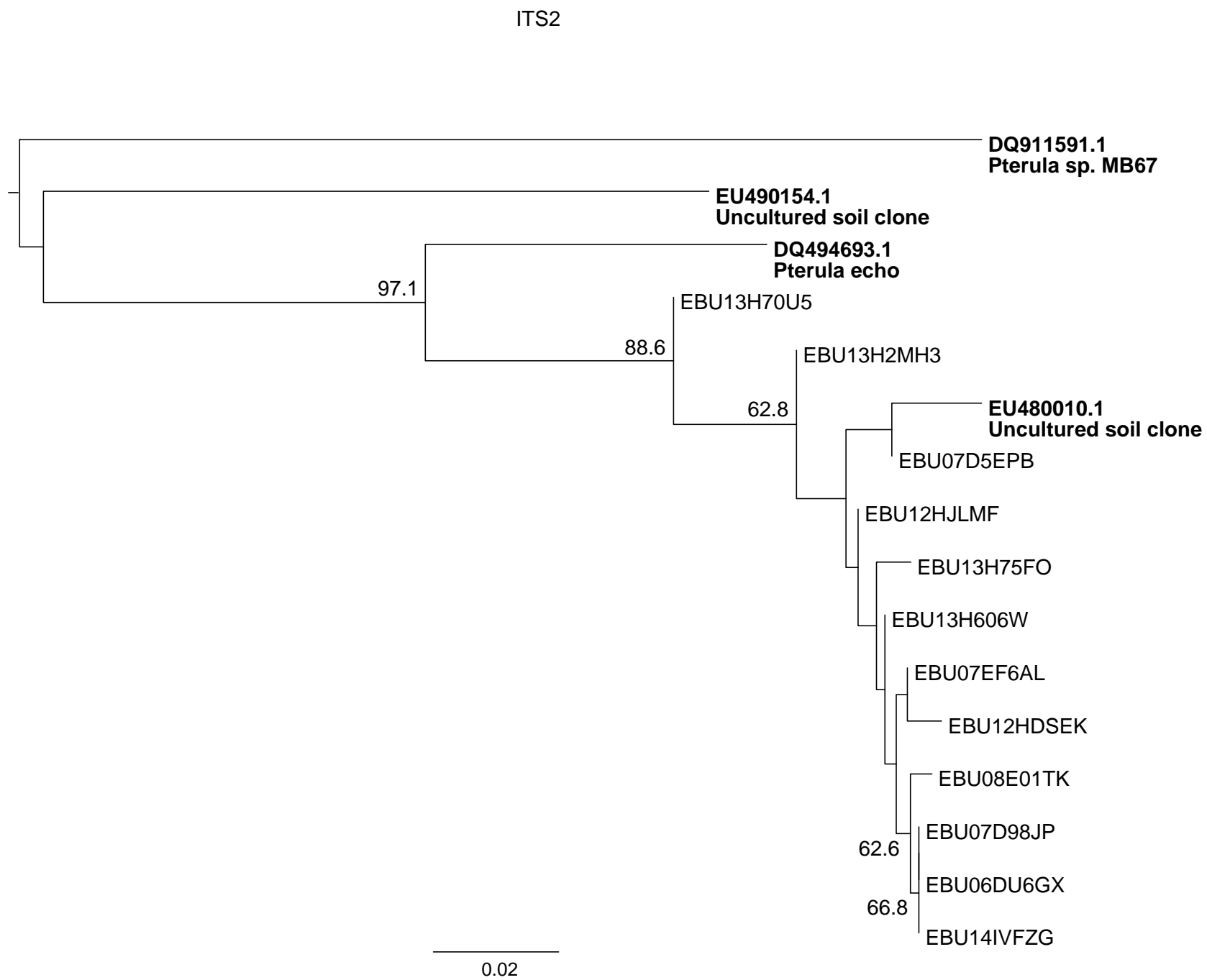

Supplement: S5 Fig — ITS1 analysis used 21 taxa, including four reference sequences from GenBank, and 279 aligned characters. ITS2 analysis used 16 taxa, including four reference sequences, and 242 aligned characters. Neighbor joining analysis used the Kimura two parameter model. 1000 neighbor joining bootstrap (NJB) replicates were conducted and clades supported by greater than 60% NJB are labeled at the nodes. (PDF) [file pone.0142759.s005.pdf]
