# Supplementary material for: Ribosomal DNA and Plastid Markers Used to Sample Fungal and Plant Communities from Wetland Soils Reveals Complementary Biotas
Source: PLoS One. 2016 Jan 5;11(1):e0142759. doi: 10.1371/journal.pone.0142759 (PMC4712138; doi:10.1371/journal.pone.0142759)
Supplement: S1 Table — (DOCX) [file pone.0142759.s006.docx]

**S1 Table. Raw read statistics for each library after sorting by primer sequence.**

|  |  |  |  | **Raw reads^1^** | | **Quality trimmed reads** | | **OTUs** | |
| --- | --- | --- | --- | --- | --- | --- | --- | --- | --- |
| **Marker** | **Site** | **Replicate** | **Primer** | **Number of reads** | **Mean length (bp)** | **Number of reads** | **Mean length (bp)** | **Number of seeds^2^** | **Mean length (bp)** |
| ITS | A | 1 | ITS1F | 14,633 | 275.0 | 11,337 | 179.9 | 1,527 | 184.2 |
|  |  |  | ITS4 | 8,690 | 220.1 | 5,023 | 196.2 | 989 | 185.1 |
|  |  | 2 | ITS1F | 13,176 | 309.1 | 10,392 | 198.6 | 1,509 | 197.8 |
|  |  |  | ITS4 | 8,801 | 245.6 | 5,271 | 220.4 | 958 | 203.3 |
|  |  | 3 | ITS1F | 13,879 | 263.6 | 10,511 | 173.6 | 1,428 | 182.5 |
|  |  |  | ITS4 | 9,156 | 211.5 | 5,138 | 191.4 | 985 | 184.5 |
|  | B | 1 | ITS1F | 15,599 | 289.4 | 11,924 | 192.5 | 1,609 | 195.3 |
|  |  |  | ITS4 | 9,303 | 243.9 | 5,804 | 213.1 | 1,017 | 198.4 |
|  |  | 2 | ITS1F | 11,720 | 282.5 | 9,007 | 194.6 | 1,302 | 190.9 |
|  |  |  | ITS4 | 8,673 | 237.0 | 5,593 | 210.4 | 912 | 193.1 |
|  |  | 3 | ITS1F | 12,448 | 286.7 | 9,424 | 200.5 | 1,355 | 198.0 |
|  |  |  | ITS4 | 9,104 | 247.3 | 5,855 | 225.6 | 977 | 204.1 |
| LSU | A | 1 | LR0R | 1,798 | 249.4 | 1,338 | 184.4 | 416 | 196.3 |
|  |  |  | LR5 | 4,749 | 237.3 | 4,155 | 159.6 | 381 | 174.9 |
|  |  | 2 | LR0R | 1,597 | 285.1 | 1,254 | 209.6 | 392 | 205.4 |
|  |  |  | LR5 | 4,180 | 273.0 | 3,802 | 186.6 | 397 | 193.1 |
|  |  | 3 | LR0R | 1,828 | 242.2 | 1,357 | 176.7 | 417 | 171.2 |
|  |  |  | LR5 | 4,595 | 239.5 | 4,086 | 158.9 | 387 | 180.8 |
|  | B | 1 | LR0R | 2,455 | 272.5 | 1,932 | 198.7 | 532 | 203.2 |
|  |  |  | LR5 | 4,677 | 257.5 | 4,243 | 178.7 | 410 | 185.1 |
|  |  | 2 | LR0R | 2,254 | 260.4 | 1,774 | 191.2 | 512 | 187.6 |
|  |  |  | LR5 | 3,591 | 240.3 | 3,266 | 170.9 | 310 | 177.0 |
|  |  | 3 | LR0R | 2,126 | 273.7 | 1,664 | 209.1 | 465 | 204.0 |
|  |  |  | LR5 | 3,590 | 254.3 | 3,272 | 179.2 | 346 | 186.7 |
| rbcL | A | 1 | a_f | 2,268 | 329.9 | 1,996 | 272.0 | 83 | 215.9 |
|  |  |  | a_r | 3,888 | 408.1 | 3,421 | 268.2 | 110 | 197.2 |
|  |  | 2 | a_f | 1,746 | 275.2 | 1,386 | 208.7 | 72 | 170.1 |
|  |  |  | a_r | 3,340 | 335.4 | 2,569 | 196.8 | 135 | 158.5 |
|  |  | 3 | a_f | 2,728 | 277.4 | 2,250 | 220.1 | 79 | 172.9 |
|  |  |  | a_r | 3,681 | 342.3 | 3,080 | 246.2 | 107 | 173.7 |
|  | B | 1 | a_f | 1,296 | 353.2 | 1,139 | 235.0 | 46 | 170.7 |
|  |  |  | a_r | 1,993 | 409.2 | 1,664 | 223.6 | 61 | 182.2 |
|  |  | 2 | a_f | 1,481 | 349.4 | 1,301 | 242.7 | 33 | 234.5 |
|  |  |  | a_r | 2,054 | 412.6 | 1,754 | 261.9 | 53 | 226.1 |
|  |  | 3 | a_f | 1,344 | 301.9 | 1,151 | 230.0 | 38 | 194.9 |
|  |  |  | a_r | 2,072 | 374.0 | 1,743 | 240.6 | 53 | 184.5 |

^1^Includes primer sequence.

^2^The first read in a new OTU is referred to as the seed.
