# Supplementary material for: Ribosomal DNA and Plastid Markers Used to Sample Fungal and Plant Communities from Wetland Soils Reveals Complementary Biotas
Source: PLoS One. 2016 Jan 5;11(1):e0142759. doi: 10.1371/journal.pone.0142759 (PMC4712138; doi:10.1371/journal.pone.0142759)
Supplement: S2 Table — (DOCX) [file pone.0142759.s007.docx]

**S2 Table. Number of OTUs from order-level MEGAN classifications using GenBank taxonomy.**

|  |  |  | **ITS** | | | | **LSU** | | | | **rbcL** | | | |
| --- | --- | --- | --- | --- | --- | --- | --- | --- | --- | --- | --- | --- | --- | --- |
|  |  |  | **5’** | | **3’** | | **5’** | | **3’** | | **5’** | | **3’** | |
| **NCBI Taxonomy** | | | **A** | **B** | **A** | **B** | **A** | **B** | **A** | **B** | **A** | **B** | **A** | **B** |
| Bacteria |  |  |  |  |  |  |  |  |  |  |  |  |  |  |
|  | Proteobacteria | Caulobacterales | 0 | 0 | 0 | 0 | 0 | 0 | 0 | 0 | 0 | 0 | 1 | 0 |
|  |  | Rhizobiales | 0 | 0 | 0 | 0 | 0 | 0 | 0 | 0 | 0 | 0 | 1 | 0 |
|  |  | Chromatiales | 0 | 0 | 0 | 0 | 0 | 0 | 0 | 0 | 1 | 0 | 5 | 0 |
| Alveolata |  |  |  |  |  |  |  |  |  |  |  |  |  |  |
|  | Ciliophora | Euplotida | 0 | 0 | 0 | 0 | 1 | 0 | 0 | 0 | 0 | 0 | 0 | 0 |
|  |  | Sporadotrichida | 0 | 0 | 0 | 0 | 0 | 2 | 6 | 3 | 0 | 0 | 0 | 0 |
|  |  | Stichotrichida | 0 | 0 | 0 | 0 | 2 | 4 | 0 | 0 | 0 | 0 | 0 | 0 |
|  |  | Urostylida | 0 | 0 | 0 | 0 | 1 | 3 | 9 | 8 | 0 | 0 | 0 | 0 |
| Fungi |  |  |  |  |  |  |  |  |  |  |  |  |  |  |
|  | Blastocladiomycota | Blastocladiales | 5 | 8 | 1 | 1 | 0 | 0 | 2 | 3 | 0 | 0 | 0 | 0 |
|  | Chytridiomycota | Chytridiales | 25 | 27 | 11 | 12 | 2 | 2 | 10 | 10 | 0 | 0 | 0 | 0 |
|  |  | Cladochytriales | 0 | 0 | 2 | 3 | 0 | 1 | 1 | 2 | 0 | 0 | 0 | 0 |
|  |  | Rhizophydiales | 4 | 4 | 3 | 4 | 2 | 4 | 5 | 5 | 0 | 0 | 0 | 0 |
|  |  | Spizellomycetales | 5 | 2 | 1 | 1 | 1 | 2 | 9 | 8 | 0 | 0 | 0 | 0 |
|  |  | Monoblepharidales | 14 | 17 | 10 | 8 | 0 | 0 | 0 | 1 | 0 | 0 | 0 | 0 |
|  |  | unclassified Chytridiomycota | 1 | 0 | 3 | 8 | 2 | 4 | 0 | 0 | 0 | 0 | 0 | 0 |
|  | Ascomycota | Ascomycota incertae sedis | 2 | 4 | 0 | 0 | 3 | 3 | 0 | 0 | 0 | 0 | 0 | 0 |
|  |  | mitosporic Ascomycota | 39 | 35 | 18 | 22 | 9 | 8 | 5 | 10 | 0 | 0 | 0 | 0 |
|  |  | Botryosphaeriales | 0 | 0 | 0 | 0 | 1 | 2 | 0 | 0 | 0 | 0 | 0 | 0 |
|  |  | Tubeufiaceae | 0 | 0 | 1 | 2 | 0 | 1 | 0 | 0 | 0 | 0 | 0 | 0 |
|  |  | Venturiaceae | 5 | 6 | 0 | 1 | 0 | 1 | 0 | 0 | 0 | 0 | 0 | 0 |
|  |  | Capnodiales | 43 | 49 | 18 | 11 | 7 | 8 | 5 | 2 | 0 | 0 | 0 | 0 |
|  |  | Dothideales | 1 | 1 | 0 | 0 | 0 | 0 | 0 | 0 | 0 | 0 | 0 | 0 |
|  |  | Myriangiales | 0 | 0 | 0 | 0 | 0 | 1 | 0 | 0 | 0 | 0 | 0 | 0 |
|  |  | mitosporic Dothideomycetes | 0 | 0 | 0 | 1 | 0 | 0 | 0 | 0 | 0 | 0 | 0 | 0 |
|  |  | Mytilinidiales | 0 | 0 | 0 | 0 | 0 | 0 | 1 | 0 | 0 | 0 | 0 | 0 |
|  |  | Pleosporales | 54 | 65 | 43 | 36 | 9 | 14 | 8 | 11 | 0 | 0 | 0 | 0 |
|  |  | unclassified Dothideomycetes | 0 | 0 | 1 | 0 | 0 | 0 | 0 | 0 | 0 | 0 | 0 | 0 |
|  |  | Chaetothyriales | 5 | 2 | 0 | 0 | 0 | 0 | 1 | 0 | 0 | 0 | 0 | 0 |
|  |  | Eurotiales | 0 | 0 | 1 | 0 | 0 | 0 | 0 | 0 | 0 | 0 | 0 | 0 |
|  |  | Onygenales | 2 | 1 | 0 | 0 | 0 | 0 | 0 | 0 | 0 | 0 | 0 | 0 |
|  |  | Umbilicariales | 5 | 3 | 0 | 0 | 0 | 0 | 0 | 0 | 0 | 0 | 0 | 0 |
|  |  | Lecanorales | 0 | 0 | 0 | 0 | 0 | 0 | 2 | 3 | 0 | 0 | 0 | 0 |
|  |  | Peltigerales | 6 | 6 | 0 | 1 | 0 | 0 | 0 | 0 | 0 | 0 | 0 | 0 |
|  |  | Agyriales | 0 | 0 | 0 | 0 | 0 | 0 | 0 | 1 | 0 | 0 | 0 | 0 |
|  |  | Ostropales | 0 | 0 | 0 | 0 | 0 | 0 | 1 | 1 | 0 | 0 | 0 | 0 |
|  |  | Lichinales | 0 | 0 | 1 | 0 | 0 | 0 | 0 | 0 | 0 | 0 | 0 | 0 |
|  |  | Pyxidiophorales | 0 | 0 | 0 | 0 | 0 | 0 | 2 | 3 | 0 | 0 | 0 | 0 |
|  |  | Cyttariales | 0 | 0 | 0 | 0 | 4 | 5 | 0 | 0 | 0 | 0 | 0 | 0 |
|  |  | Erysiphales | 0 | 0 | 1 | 0 | 2 | 2 | 0 | 0 | 0 | 0 | 0 | 0 |
|  |  | Helotiales | 78 | 73 | 54 | 54 | 20 | 36 | 14 | 10 | 0 | 0 | 0 | 0 |
|  |  | Leotiomycetes incertae sedis | 19 | 16 | 24 | 17 | 2 | 4 | 1 | 3 | 0 | 0 | 0 | 0 |
|  |  | mitosporic Leotiomycetes | 2 | 5 | 0 | 0 | 0 | 0 | 0 | 0 | 0 | 0 | 0 | 0 |
|  |  | Rhytismatales | 0 | 0 | 0 | 0 | 0 | 0 | 0 | 1 | 0 | 0 | 0 | 0 |
|  |  | Thelebolales | 1 | 0 | 1 | 1 | 0 | 1 | 0 | 0 | 0 | 0 | 0 | 0 |
|  |  | Hypocreales | 53 | 47 | 25 | 32 | 16 | 22 | 15 | 13 | 0 | 0 | 0 | 0 |
|  |  | Microascales | 10 | 8 | 7 | 4 | 2 | 3 | 4 | 3 | 0 | 0 | 0 | 0 |
|  |  | Phlogicylindrium | 2 | 0 | 0 | 0 | 0 | 0 | 0 | 0 | 0 | 0 | 0 | 0 |
|  |  | Phyllachorales | 15 | 15 | 10 | 13 | 0 | 0 | 0 | 0 | 0 | 0 | 0 | 0 |
|  |  | Trichosphaeriales | 0 | 0 | 0 | 0 | 0 | 0 | 0 | 1 | 0 | 0 | 0 | 0 |
|  |  | Wallrothiella | 0 | 0 | 0 | 0 | 0 | 0 | 0 | 1 | 0 | 0 | 0 | 0 |
|  |  | Coniochaetales | 0 | 0 | 0 | 0 | 0 | 0 | 1 | 0 | 0 | 0 | 0 | 0 |
|  |  | Diaporthales | 1 | 0 | 0 | 0 | 0 | 0 | 0 | 0 | 0 | 0 | 0 | 0 |
|  |  | Magnaporthales | 5 | 7 | 3 | 1 | 0 | 0 | 0 | 0 | 0 | 0 | 0 | 0 |
|  |  | Ophiostomatales | 1 | 0 | 0 | 1 | 0 | 0 | 0 | 0 | 0 | 0 | 0 | 0 |
|  |  | Sordariales | 22 | 28 | 8 | 23 | 9 | 12 | 8 | 5 | 0 | 0 | 0 | 0 |
|  |  | Sordariomycetidae incertae sedis | 0 | 0 | 0 | 0 | 1 | 0 | 0 | 0 | 0 | 0 | 0 | 0 |
|  |  | unclassified Sordariomycetes | 0 | 0 | 0 | 0 | 0 | 1 | 0 | 0 | 0 | 0 | 0 | 0 |
|  |  | Xylariales | 6 | 4 | 1 | 4 | 1 | 0 | 3 | 0 | 0 | 0 | 0 | 0 |
|  |  | Orbiliales | 4 | 1 | 0 | 0 | 0 | 1 | 2 | 3 | 0 | 0 | 0 | 0 |
|  |  | Pezizales | 120 | 115 | 48 | 47 | 19 | 30 | 25 | 24 | 0 | 0 | 0 | 0 |
|  |  | unclassified Pezizomycetes | 1 | 0 | 6 | 3 | 0 | 0 | 0 | 0 | 0 | 0 | 0 | 0 |
|  |  | unclassified Pezizomycotina | 2 | 1 | 0 | 0 | 0 | 0 | 0 | 0 | 0 | 0 | 0 | 0 |
|  |  | Saccharomycetales | 5 | 7 | 2 | 2 | 0 | 0 | 5 | 6 | 0 | 0 | 0 | 0 |
|  |  | Pneumocystidales | 0 | 0 | 0 | 0 | 0 | 1 | 0 | 0 | 0 | 0 | 0 | 0 |
|  |  | Schizosaccharo-mycetales | 0 | 2 | 0 | 0 | 0 | 0 | 0 | 1 | 0 | 0 | 0 | 0 |
|  |  | unclassified Ascomycota | 23 | 25 | 2 | 0 | 0 | 0 | 0 | 0 | 0 | 0 | 0 | 0 |
|  | Basidiomycota | Auriculariales | 3 | 2 | 5 | 4 | 0 | 3 | 1 | 2 | 0 | 0 | 0 | 0 |
|  |  | Cantharellales | 1 | 2 | 5 | 5 | 6 | 2 | 3 | 4 | 0 | 0 | 0 | 0 |
|  |  | Corticiales | 13 | 11 | 7 | 7 | 4 | 7 | 0 | 0 | 0 | 0 | 0 | 0 |
|  |  | Hymenochaetales | 0 | 2 | 3 | 3 | 0 | 0 | 0 | 0 | 0 | 0 | 0 | 0 |
|  |  | Polyporales | 29 | 21 | 12 | 20 | 9 | 12 | 10 | 14 | 0 | 0 | 0 | 0 |
|  |  | Russulales | 1 | 1 | 0 | 0 | 0 | 0 | 1 | 0 | 0 | 0 | 0 | 0 |
|  |  | Sebacinales | 7 | 4 | 3 | 3 | 0 | 0 | 1 | 2 | 0 | 0 | 0 | 0 |
|  |  | Thelephorales | 37 | 37 | 18 | 19 | 3 | 3 | 4 | 5 | 0 | 0 | 0 | 0 |
|  |  | Trechisporales | 0 | 0 | 0 | 0 | 0 | 1 | 0 | 0 | 0 | 0 | 0 | 0 |
|  |  | Agaricales | 58 | 45 | 42 | 49 | 10 | 9 | 7 | 11 | 0 | 0 | 0 | 0 |
|  |  | Boletales | 2 | 3 | 0 | 0 | 1 | 0 | 1 | 2 | 0 | 0 | 0 | 0 |
|  |  | Gomphales | 0 | 0 | 0 | 0 | 4 | 4 | 4 | 6 | 0 | 0 | 0 | 0 |
|  |  | Hysterangiales | 1 | 1 | 0 | 0 | 0 | 0 | 0 | 0 | 0 | 0 | 0 | 0 |
|  |  | mitosporic Agaricomycotina | 6 | 5 | 0 | 0 | 0 | 0 | 0 | 0 | 0 | 0 | 0 | 0 |
|  |  | Cystofilobasidiales | 20 | 23 | 12 | 15 | 4 | 2 | 6 | 7 | 0 | 0 | 0 | 0 |
|  |  | Filobasidiales | 9 | 8 | 5 | 3 | 0 | 0 | 3 | 5 | 0 | 0 | 0 | 0 |
|  |  | Tremellales | 40 | 31 | 18 | 17 | 2 | 2 | 8 | 9 | 0 | 0 | 0 | 0 |
|  |  | Wallemiales | 0 | 0 | 0 | 0 | 0 | 0 | 1 | 0 | 0 | 0 | 0 | 0 |
|  |  | mitosporic Basidiomycota | 2 | 0 | 0 | 1 | 0 | 0 | 0 | 0 | 0 | 0 | 0 | 0 |
|  |  | Agaricostilbo-mycetes incertae sedis | 2 | 4 | 1 | 1 | 0 | 0 | 0 | 0 | 0 | 0 | 0 | 0 |
|  |  | Atractiellales | 0 | 0 | 1 | 1 | 0 | 0 | 0 | 0 | 0 | 0 | 0 | 0 |
|  |  | Erythrobasidiales | 12 | 1 | 7 | 2 | 2 | 3 | 3 | 0 | 0 | 0 | 0 | 0 |
|  |  | Leucosporidiales | 4 | 4 | 3 | 3 | 0 | 2 | 3 | 2 | 0 | 0 | 0 | 0 |
|  |  | Microbotryomycetes incertae sedis | 5 | 7 | 5 | 5 | 1 | 1 | 1 | 2 | 0 | 0 | 0 | 0 |
|  |  | Sporidiobolales | 14 | 7 | 4 | 2 | 0 | 0 | 2 | 0 | 0 | 0 | 0 | 0 |
|  |  | Platygloeales | 20 | 16 | 5 | 5 | 4 | 2 | 15 | 11 | 0 | 0 | 0 | 0 |
|  |  | Pucciniales | 8 | 9 | 6 | 6 | 0 | 2 | 0 | 0 | 0 | 0 | 0 | 0 |
|  |  | Septobasidiales | 0 | 0 | 0 | 0 | 4 | 3 | 0 | 0 | 0 | 0 | 0 | 0 |
|  |  | unclassified Basidiomycota | 3 | 3 | 0 | 0 | 0 | 0 | 0 | 0 | 0 | 0 | 0 | 0 |
|  |  | Doassansiales | 0 | 0 | 0 | 0 | 0 | 0 | 0 | 1 | 0 | 0 | 0 | 0 |
|  |  | Entylomatales | 3 | 5 | 3 | 4 | 0 | 1 | 2 | 1 | 0 | 0 | 0 | 0 |
|  |  | Georgefischeriales | 0 | 1 | 0 | 0 | 0 | 0 | 1 | 0 | 0 | 0 | 0 | 0 |
|  |  | Malasseziales | 8 | 3 | 0 | 1 | 2 | 0 | 0 | 0 | 0 | 0 | 0 | 0 |
|  |  | Urocystales | 3 | 2 | 0 | 0 | 0 | 0 | 0 | 1 | 0 | 0 | 0 | 0 |
|  |  | Ustilaginales | 2 | 4 | 0 | 0 | 3 | 0 | 3 | 0 | 0 | 0 | 0 | 0 |
|  | Basal fungal lineages | Entomophthorales | 1 | 3 | 0 | 0 | 0 | 0 | 3 | 2 | 0 | 0 | 0 | 0 |
|  |  | Kickxellales | 0 | 0 | 4 | 5 | 0 | 0 | 0 | 0 | 0 | 0 | 0 | 0 |
|  |  | Mortierellales | 21 | 20 | 6 | 10 | 2 | 3 | 3 | 3 | 0 | 0 | 0 | 0 |
|  |  | Mucorales | 6 | 5 | 4 | 5 | 0 | 0 | 0 | 0 | 0 | 0 | 0 | 0 |
|  |  | Zoopagales | 1 | 0 | 0 | 0 | 0 | 0 | 4 | 2 | 0 | 0 | 0 | 0 |
|  | Glomeromycota | Archaeosporales | 5 | 2 | 2 | 3 | 0 | 0 | 0 | 0 | 0 | 0 | 0 | 0 |
|  |  | Diversisporales | 9 | 6 | 0 | 0 | 1 | 0 | 3 | 3 | 0 | 0 | 0 | 0 |
|  |  | Glomerales | 45 | 37 | 16 | 18 | 1 | 1 | 1 | 0 | 0 | 0 | 0 | 0 |
|  |  | Paraglomerales | 0 | 0 | 3 | 2 | 1 | 0 | 1 | 1 | 0 | 0 | 0 | 0 |
|  | Neocallimastigomycota | Neocallimastigales | 2 | 2 | 0 | 0 | 1 | 2 | 0 | 0 | 0 | 0 | 0 | 0 |
| Fungi/Metazoa incertae sedis | |  |  |  |  |  |  |  |  |  |  |  |  |  |
|  |  | Dermocystida | 1 | 0 | 4 | 3 | 0 | 0 | 0 | 0 | 0 | 0 | 0 | 0 |
|  |  | Ichthyophonida | 4 | 8 | 0 | 3 | 0 | 0 | 0 | 0 | 0 | 0 | 0 | 0 |
| Metazoa |  |  |  |  |  |  |  |  |  |  |  |  |  |  |
|  | Platyhelminthes | Cyclophyllidea | 0 | 0 | 0 | 0 | 1 | 0 | 0 | 0 | 0 | 0 | 0 | 0 |
|  |  | Monopisthocotylea | 6 | 2 | 0 | 0 | 0 | 0 | 0 | 0 | 0 | 0 | 0 | 0 |
|  | Echinodermata | Valvatida | 0 | 0 | 0 | 0 | 0 | 0 | 1 | 0 | 0 | 0 | 0 | 0 |
|  | Annelida | Phyllodocida | 0 | 0 | 1 | 0 | 0 | 0 | 0 | 0 | 0 | 0 | 0 | 0 |
|  | Mollusca | Myoida | 0 | 0 | 0 | 0 | 0 | 0 | 1 | 1 | 0 | 0 | 0 | 0 |
|  |  | Solemyoida | 0 | 0 | 0 | 0 | 1 | 0 | 0 | 0 | 0 | 0 | 0 | 0 |
|  | Arthropoda | Mesostigmata | 0 | 0 | 1 | 0 | 0 | 0 | 0 | 1 | 0 | 0 | 0 | 0 |
|  |  | Araneae | 0 | 0 | 0 | 0 | 0 | 0 | 0 | 1 | 0 | 0 | 0 | 0 |
|  |  | Poecilostomatoida | 0 | 0 | 0 | 0 | 0 | 1 | 0 | 0 | 0 | 0 | 0 | 0 |
|  | Tardigrada | Arthrotardigrada | 0 | 0 | 0 | 0 | 0 | 0 | 2 | 3 | 0 | 0 | 0 | 0 |
|  | Gastrotricha | Macrodasyida | 0 | 0 | 0 | 0 | 0 | 0 | 1 | 0 | 0 | 0 | 0 | 0 |
|  | Nematoda | Araeolaimida | 0 | 0 | 0 | 0 | 2 | 3 | 2 | 3 | 0 | 0 | 0 | 0 |
|  |  | Monhysterida | 0 | 0 | 0 | 0 | 0 | 0 | 0 | 1 | 0 | 0 | 0 | 0 |
|  |  | Oxyurida | 0 | 0 | 1 | 2 | 0 | 0 | 0 | 0 | 0 | 0 | 0 | 0 |
|  |  | Rhabditida | 0 | 0 | 0 | 0 | 6 | 10 | 11 | 10 | 0 | 0 | 0 | 0 |
|  |  | Tylenchida | 0 | 0 | 0 | 0 | 5 | 11 | 20 | 21 | 0 | 0 | 0 | 0 |
|  |  | Enoplida | 0 | 0 | 0 | 0 | 0 | 0 | 2 | 0 | 0 | 0 | 0 | 0 |
|  |  | Triplonchida | 0 | 0 | 0 | 0 | 0 | 0 | 0 | 1 | 0 | 0 | 0 | 0 |
|  | Rotifera | Ploimida | 1 | 1 | 0 | 0 | 0 | 0 | 0 | 0 | 0 | 0 | 0 | 0 |
|  | Cnidaria | Hydroida | 0 | 0 | 0 | 0 | 0 | 0 | 2 | 1 | 0 | 0 | 0 | 0 |
|  | Porifera | Leucosolenida | 8 | 6 | 0 | 0 | 0 | 0 | 0 | 0 | 0 | 0 | 0 | 0 |
| Katablepharidophyta | |  |  |  |  |  |  |  |  |  |  |  |  |  |
|  |  | Katablepharido-phyta | 1 | 0 | 0 | 0 | 0 | 0 | 0 | 0 | 0 | 0 | 0 | 0 |
| Rhizaria |  |  |  |  |  |  |  |  |  |  |  |  |  |  |
|  | Cercozoa | Cercomonadida | 1 | 4 | 1 | 4 | 0 | 0 | 0 | 0 | 0 | 0 | 0 | 0 |
|  |  | Plasmodiophorida | 1 | 0 | 0 | 0 | 0 | 0 | 0 | 0 | 0 | 0 | 0 | 0 |
|  |  | Thaumatomonadida | 0 | 1 | 0 | 0 | 0 | 0 | 0 | 0 | 0 | 0 | 0 | 0 |
|  |  | unclassified Cercozoa | 10 | 5 | 0 | 0 | 0 | 0 | 0 | 0 | 0 | 0 | 0 | 0 |
| Rhodophyta | |  |  |  |  |  |  |  |  |  |  |  |  |  |
|  |  | Erythropeltidales | 3 | 1 | 0 | 0 | 0 | 0 | 0 | 0 | 0 | 0 | 0 | 0 |
|  |  | Gigartinales | 0 | 0 | 1 | 3 | 0 | 0 | 0 | 0 | 0 | 0 | 0 | 0 |
| stramenopiles | |  |  |  |  |  |  |  |  |  |  |  |  |  |
|  |  | Developayella | 0 | 0 | 0 | 0 | 0 | 0 | 3 | 3 | 0 | 0 | 0 | 0 |
|  | Oomycetes | Peronosporales | 0 | 0 | 0 | 0 | 2 | 3 | 3 | 3 | 0 | 0 | 0 | 0 |
|  |  | Pythiales | 0 | 0 | 0 | 0 | 5 | 4 | 3 | 1 | 0 | 0 | 0 | 0 |
|  | PX clade | Vaucheriales | 0 | 1 | 0 | 0 | 0 | 0 | 0 | 0 | 0 | 0 | 0 | 0 |
| Viridiplantae | |  |  |  |  |  |  |  |  |  |  |  |  |  |
|  | Chlorophyta | Bryopsidales | 2 | 1 | 0 | 0 | 0 | 0 | 0 | 0 | 0 | 0 | 0 | 0 |
|  | Streptophyta | Bryales | 0 | 0 | 0 | 0 | 0 | 0 | 0 | 0 | 11 | 5 | 22 | 5 |
|  |  | Grimmiales | 0 | 0 | 0 | 0 | 0 | 0 | 0 | 0 | 0 | 2 | 0 | 0 |
|  |  | Pottiales | 0 | 0 | 0 | 0 | 0 | 0 | 0 | 0 | 0 | 0 | 0 | 2 |
|  |  | Funariales | 0 | 0 | 0 | 0 | 0 | 0 | 0 | 0 | 0 | 0 | 0 | 1 |
|  |  | Hypnales | 0 | 0 | 0 | 0 | 0 | 0 | 0 | 0 | 3 | 0 | 0 | 1 |
|  |  | Ricciales | 0 | 0 | 0 | 0 | 0 | 0 | 0 | 0 | 6 | 3 | 14 | 9 |
|  |  | Aneurales | 1 | 1 | 0 | 1 | 0 | 0 | 0 | 0 | 0 | 0 | 0 | 0 |
|  |  | Equisetales | 0 | 0 | 0 | 0 | 0 | 0 | 0 | 0 | 8 | 8 | 22 | 19 |
|  |  | Asterales | 0 | 0 | 0 | 0 | 0 | 0 | 0 | 0 | 14 | 7 | 9 | 7 |
|  |  | Cornales | 0 | 0 | 0 | 0 | 0 | 0 | 0 | 0 | 0 | 0 | 0 | 1 |
|  |  | Lamiales | 1 | 0 | 0 | 0 | 0 | 0 | 1 | 0 | 8 | 4 | 22 | 14 |
|  |  | Solanales | 0 | 0 | 0 | 0 | 0 | 0 | 0 | 0 | 0 | 1 | 1 | 0 |
|  |  | Malpighiales | 0 | 0 | 0 | 0 | 0 | 0 | 0 | 0 | 5 | 5 | 16 | 14 |
|  |  | Rosales | 0 | 0 | 0 | 0 | 0 | 0 | 0 | 0 | 3 | 0 | 8 | 0 |
|  |  | Brassicales | 0 | 0 | 0 | 0 | 0 | 0 | 0 | 0 | 3 | 0 | 4 | 0 |
|  |  | Acorales | 0 | 0 | 0 | 0 | 0 | 0 | 0 | 0 | 10 | 17 | 17 | 20 |
|  |  | Commelinales | 0 | 0 | 0 | 0 | 0 | 0 | 0 | 0 | 1 | 0 | 0 | 0 |
|  |  | Poales | 4 | 5 | 4 | 3 | 1 | 1 | 2 | 0 | 43 | 10 | 64 | 16 |
