# Supplementary material for: Ribosomal DNA and Plastid Markers Used to Sample Fungal and Plant Communities from Wetland Soils Reveals Complementary Biotas
Source: PLoS One. 2016 Jan 5;11(1):e0142759. doi: 10.1371/journal.pone.0142759 (PMC4712138; doi:10.1371/journal.pone.0142759)
Supplement: S3 Table — (DOCX) [file pone.0142759.s008.docx]

**S3 Table. Number of OTUs from species-level MEGAN classifications using GenBank taxonomy.**

|  | |  | |  | **ITS** | | | | **LSU** | | | | **rbcL** | | | |
| --- | --- | --- | --- | --- | --- | --- | --- | --- | --- | --- | --- | --- | --- | --- | --- | --- |
|  | |  | |  | **5’** | | **3’** | | **5’** | | **3’** | | **5’** | | **3’** | |
| **NCBI Taxonomy** | | | | | **A** | **B** | **A** | **B** | **A** | **B** | **A** | **B** | **A** | **B** | **A** | **B** |
| Proteobacteria | | |  | |  |  |  |  |  |  |  |  |  |  |  |  |
|  | Caulobacterales | | Caulobacter segnis | | 0 | 0 | 0 | 0 | 0 | 0 | 0 | 0 | 0 | 0 | 1 | 0 |
|  | Rhizobiales | | Starkeya novella | | 0 | 0 | 0 | 0 | 0 | 0 | 0 | 0 | 0 | 0 | 1 | 0 |
|  | Chromatiales | | Allochromatium vinosum | | 0 | 0 | 0 | 0 | 0 | 0 | 0 | 0 | 0 | 0 | 2 | 0 |
|  |  | | Thioalkalivibrio sp. K90mix | | 0 | 0 | 0 | 0 | 0 | 0 | 0 | 0 | 1 | 0 | 3 | 0 |
| Alveolata | | |  | |  |  |  |  |  |  |  |  |  |  |  |  |
|  | Euplotida | | Uronychia | | 0 | 0 | 0 | 0 | 1 | 0 | 0 | 0 | 0 | 0 | 0 | 0 |
|  | Sporadotrichida | | Oxytricha granulifera | | 0 | 0 | 0 | 0 | 0 | 0 | 1 | 0 | 0 | 0 | 0 | 0 |
|  |  | | Sterkiella histriomuscorum | | 0 | 0 | 0 | 0 | 0 | 2 | 0 | 0 | 0 | 0 | 0 | 0 |
|  | Stichotrichida | | Engelmanniella mobilis | | 0 | 0 | 0 | 0 | 2 | 4 | 0 | 0 | 0 | 0 | 0 | 0 |
|  | Urostylida | | Holosticha polystylata | | 0 | 0 | 0 | 0 | 0 | 0 | 1 | 0 | 0 | 0 | 0 | 0 |
|  |  | | Paruroleptus lepisma | | 0 | 0 | 0 | 0 | 0 | 0 | 2 | 0 | 0 | 0 | 0 | 0 |
|  |  | | Uroleptus gallina | | 0 | 0 | 0 | 0 | 1 | 0 | 5 | 7 | 0 | 0 | 0 | 0 |
|  |  | | Urostyla grandis | | 0 | 0 | 0 | 0 | 0 | 3 | 0 | 0 | 0 | 0 | 0 | 0 |
| Blastocladiomycota | | |  | |  |  |  |  |  |  |  |  |  |  |  |  |
|  | Blastocladiales | | Allomyces moniliformis | | 0 | 1 | 0 | 0 | 0 | 0 | 0 | 2 | 0 | 0 | 0 | 0 |
|  |  | | Blastocladiella britannica | | 0 | 0 | 0 | 0 | 0 | 0 | 2 | 1 | 0 | 0 | 0 | 0 |
|  |  | | Catenaria anguillulae | | 1 | 0 | 0 | 0 | 0 | 0 | 0 | 0 | 0 | 0 | 0 | 0 |
|  |  | | Catenomyces sp. JEL342 | | 3 | 6 | 1 | 1 | 0 | 0 | 0 | 0 | 0 | 0 | 0 | 0 |
|  |  | | Physoderma maydis | | 0 | 1 | 0 | 0 | 0 | 0 | 0 | 0 | 0 | 0 | 0 | 0 |
| Chytridiomycota | | |  | |  |  |  |  |  |  |  |  |  |  |  |  |
|  | Chytridiales | | Chytriomyces | | 2 | 1 | 6 | 8 | 1 | 0 | 1 | 0 | 0 | 0 | 0 | 0 |
|  |  | | Rhizoclosmatium sp. JEL347-h | | 0 | 0 | 1 | 1 | 0 | 0 | 0 | 0 | 0 | 0 | 0 | 0 |
|  |  | | Entophlyctis sp. JEL174 | | 1 | 3 | 3 | 2 | 0 | 2 | 0 | 0 | 0 | 0 | 0 | 0 |
|  |  | | Nowakowskiella sp. JEL127 | | 0 | 0 | 0 | 0 | 1 | 0 | 2 | 1 | 0 | 0 | 0 | 0 |
|  |  | | Synchytrium endobioticum | | 11 | 9 | 0 | 0 | 0 | 0 | 0 | 0 | 0 | 0 | 0 | 0 |
|  |  | | Chytridiales sp. PL70 | | 2 | 4 | 0 | 0 | 0 | 0 | 0 | 0 | 0 | 0 | 0 | 0 |
|  | Cladochytriales | | Cladochytrium replicatum | | 0 | 0 | 0 | 0 | 0 | 1 | 1 | 2 | 0 | 0 | 0 | 0 |
|  |  | | Polychytrium aggregatum | | 0 | 0 | 2 | 3 | 0 | 0 | 0 | 0 | 0 | 0 | 0 | 0 |
|  | Rhizophydiales | | Kappamyces sp. Barr-316 | | 1 | 1 | 0 | 0 | 0 | 0 | 0 | 0 | 0 | 0 | 0 | 0 |
|  |  | | Kappamyces sp. PL-116 | | 0 | 0 | 0 | 0 | 0 | 1 | 0 | 0 | 0 | 0 | 0 | 0 |
|  |  | | Rhizophydium brooksianum | | 0 | 0 | 0 | 0 | 2 | 2 | 0 | 0 | 0 | 0 | 0 | 0 |
|  |  | | Rhizophydium chaetiferum | | 0 | 0 | 0 | 1 | 0 | 0 | 1 | 1 | 0 | 0 | 0 | 0 |
|  |  | | Rhizophydium laterale | | 0 | 0 | 0 | 0 | 0 | 0 | 0 | 1 | 0 | 0 | 0 | 0 |
|  |  | | Rhizophydium sp. ARG055 | | 0 | 0 | 1 | 0 | 0 | 0 | 0 | 0 | 0 | 0 | 0 | 0 |
|  |  | | Rhizophydium sp. JEL-385 | | 0 | 2 | 0 | 0 | 0 | 0 | 0 | 0 | 0 | 0 | 0 | 0 |
|  |  | | Rhizophydium sp. JEL-400 | | 1 | 1 | 0 | 0 | 0 | 0 | 0 | 0 | 0 | 0 | 0 | 0 |
|  |  | | Rhizophydium sp. JEL316 | | 0 | 0 | 0 | 0 | 0 | 0 | 1 | 0 | 0 | 0 | 0 | 0 |
|  |  | | Rhizophydium sp. JEL317 | | 0 | 0 | 0 | 0 | 0 | 1 | 0 | 0 | 0 | 0 | 0 | 0 |
|  |  | | Rhizophydiales sp. JEL142 | | 0 | 0 | 1 | 0 | 0 | 0 | 0 | 0 | 0 | 0 | 0 | 0 |
|  | (Basal fungal lineages) | | Olpidium brassicae | | 2 | 0 | 0 | 1 | 0 | 0 | 0 | 0 | 0 | 0 | 0 | 0 |
|  | Spizellomycetales | | Powellomyces sp. JEL95 | | 0 | 0 | 0 | 0 | 1 | 0 | 0 | 0 | 0 | 0 | 0 | 0 |
|  |  | | Rhizophlyctis harderi | | 0 | 0 | 0 | 0 | 0 | 0 | 2 | 3 | 0 | 0 | 0 | 0 |
|  |  | | Rhizophlyctis rosea | | 1 | 0 | 0 | 0 | 0 | 0 | 2 | 2 | 0 | 0 | 0 | 0 |
|  | (Rozella clade) | | Rozella allomycis | | 0 | 0 | 1 | 0 | 0 | 0 | 0 | 0 | 0 | 0 | 0 | 0 |
|  |  | | Spizellomycetales sp. JEL 549 | | 1 | 0 | 0 | 0 | 0 | 0 | 0 | 0 | 0 | 0 | 0 | 0 |
|  | Monoblepharidales | | Hyaloraphidium curvatum | | 6 | 4 | 5 | 2 | 0 | 0 | 0 | 0 | 0 | 0 | 0 | 0 |
|  |  | | Oedogoniomyces sp. CR84 | | 0 | 0 | 0 | 0 | 0 | 0 | 0 | 1 | 0 | 0 | 0 | 0 |
|  | unclassified | | Chytridiomycota sp. Mori B3 | | 1 | 0 | 3 | 8 | 2 | 4 | 0 | 0 | 0 | 0 | 0 | 0 |
| Ascomycota | | |  | |  |  |  |  |  |  |  |  |  |  |  |  |
|  | Ascomycota incertae sedis | | Dokmaia monthadangii | | 2 | 4 | 0 | 0 | 0 | 0 | 0 | 0 | 0 | 0 | 0 | 0 |
|  |  | | Tromeropsis microtheca | | 0 | 0 | 0 | 0 | 3 | 3 | 0 | 0 | 0 | 0 | 0 | 0 |
|  | mitosporic Ascomycota | | Acremonium cereale | | 3 | 4 | 0 | 0 | 0 | 0 | 0 | 0 | 0 | 0 | 0 | 0 |
|  |  | | Cephaliophora tropica | | 0 | 0 | 1 | 1 | 0 | 0 | 0 | 0 | 0 | 0 | 0 | 0 |
|  |  | | Fonsecaea | | 0 | 0 | 0 | 0 | 0 | 0 | 0 | 1 | 0 | 0 | 0 | 0 |
|  |  | | Isthmolongispora ampulliformis | | 0 | 0 | 0 | 0 | 0 | 0 | 1 | 1 | 0 | 0 | 0 | 0 |
|  |  | | Leptodontidium orchidicola | | 4 | 0 | 0 | 0 | 0 | 0 | 0 | 0 | 0 | 0 | 0 | 0 |
|  |  | | Mycochaetophora sp. MAFF 241068 | | 0 | 0 | 0 | 0 | 1 | 0 | 0 | 0 | 0 | 0 | 0 | 0 |
|  |  | | Ochroconis constricta | | 4 | 3 | 0 | 0 | 0 | 0 | 0 | 0 | 0 | 0 | 0 | 0 |
|  |  | | Ochroconis tshawytschae | | 0 | 0 | 0 | 0 | 1 | 2 | 0 | 0 | 0 | 0 | 0 | 0 |
|  |  | | Phaeomoniella | | 0 | 0 | 0 | 0 | 1 | 0 | 0 | 0 | 0 | 0 | 0 | 0 |
|  | Pleosporales | | Phoma commelinicola | | 0 | 0 | 0 | 0 | 0 | 0 | 0 | 1 | 0 | 0 | 0 | 0 |
|  |  | | Phoma exigua | | 8 | 6 | 0 | 0 | 0 | 0 | 0 | 0 | 0 | 0 | 0 | 0 |
|  |  | | Phoma flavescens | | 0 | 0 | 0 | 0 | 0 | 0 | 0 | 1 | 0 | 0 | 0 | 0 |
|  |  | | Phoma sclerotioides | | 0 | 0 | 2 | 3 | 0 | 0 | 0 | 0 | 0 | 0 | 0 | 0 |
|  |  | | Phoma sp. H39 | | 5 | 7 | 0 | 0 | 0 | 0 | 0 | 0 | 0 | 0 | 0 | 0 |
|  |  | | Phoma sp. JS2122 | | 0 | 0 | 2 | 2 | 0 | 0 | 0 | 0 | 0 | 0 | 0 | 0 |
|  | mitosporic Ascomycota | | Pseudotaeniolina globosa | | 0 | 0 | 0 | 0 | 1 | 0 | 0 | 0 | 0 | 0 | 0 | 0 |
|  |  | | Rhynchosporium orthosporum | | 0 | 1 | 1 | 0 | 0 | 0 | 0 | 0 | 0 | 0 | 0 | 0 |
|  |  | | Scolecobasidium humicola | | 0 | 0 | 0 | 0 | 0 | 0 | 1 | 1 | 0 | 0 | 0 | 0 |
|  |  | | Spirosphaera beverwijkiana | | 0 | 0 | 0 | 1 | 0 | 0 | 0 | 0 | 0 | 0 | 0 | 0 |
|  |  | | Tetracladium breve | | 0 | 0 | 0 | 0 | 3 | 4 | 0 | 0 | 0 | 0 | 0 | 0 |
|  |  | | Tetracladium setigerum | | 0 | 0 | 0 | 0 | 0 | 0 | 1 | 0 | 0 | 0 | 0 | 0 |
|  |  | | Tricladium angulatum | | 1 | 0 | 3 | 6 | 0 | 0 | 0 | 0 | 0 | 0 | 0 | 0 |
|  |  | | Tritirachium oryzae | | 0 | 0 | 0 | 0 | 0 | 1 | 0 | 0 | 0 | 0 | 0 | 0 |
|  |  | | Zalerion arboricola | | 0 | 0 | 0 | 0 | 1 | 0 | 0 | 0 | 0 | 0 | 0 | 0 |
|  | Botryosphaeriales | | Phyllosticta flevolandica | | 0 | 0 | 0 | 0 | 1 | 2 | 0 | 0 | 0 | 0 | 0 | 0 |
|  | Dothidiomycetes incertae sedis | | Helicoma isiola | | 0 | 0 | 1 | 2 | 0 | 0 | 0 | 0 | 0 | 0 | 0 | 0 |
|  |  | | Helicomyces scandens | | 0 | 0 | 0 | 0 | 0 | 1 | 0 | 0 | 0 | 0 | 0 | 0 |
|  |  | | Venturia | | 0 | 0 | 0 | 1 | 0 | 1 | 0 | 0 | 0 | 0 | 0 | 0 |
|  | Capnodiales | | Davidiella tassiana | | 2 | 5 | 0 | 1 | 1 | 0 | 0 | 0 | 0 | 0 | 0 | 0 |
|  |  | | Cladosporium bruhnei | | 0 | 1 | 0 | 0 | 0 | 0 | 0 | 0 | 0 | 0 | 0 | 0 |
|  |  | | Cladosporium cladosporioides | | 0 | 1 | 0 | 0 | 0 | 0 | 0 | 0 | 0 | 0 | 0 | 0 |
|  |  | | Cladosporium sp. 4255 | | 0 | 1 | 0 | 0 | 0 | 0 | 0 | 0 | 0 | 0 | 0 | 0 |
|  |  | | Cladosporium sp. STE-U 5124 | | 0 | 1 | 0 | 0 | 0 | 0 | 0 | 0 | 0 | 0 | 0 | 0 |
|  |  | | Dissoconium aciculare | | 0 | 0 | 0 | 0 | 0 | 1 | 0 | 0 | 0 | 0 | 0 | 0 |
|  |  | | Cryptococcus | | 2 | 4 | 1 | 2 | 0 | 1 | 0 | 0 | 0 | 0 | 0 | 0 |
|  |  | | Pseudocercosporella fraxini | | 2 | 1 | 0 | 0 | 0 | 0 | 0 | 0 | 0 | 0 | 0 | 0 |
|  |  | | Ramularia sp. ascomyc2 | | 1 | 0 | 0 | 0 | 0 | 0 | 0 | 0 | 0 | 0 | 0 | 0 |
|  |  | | Septoria passerinii | | 0 | 0 | 1 | 1 | 0 | 0 | 0 | 0 | 0 | 0 | 0 | 0 |
|  |  | | Mycosphaerella fragariae | | 3 | 4 | 5 | 3 | 0 | 0 | 1 | 0 | 0 | 0 | 0 | 0 |
|  |  | | Mycosphaerella graminicola | | 7 | 5 | 4 | 3 | 2 | 1 | 0 | 0 | 0 | 0 | 0 | 0 |
|  |  | | Mycosphaerella pini | | 0 | 0 | 0 | 0 | 0 | 0 | 1 | 0 | 0 | 0 | 0 | 0 |
|  |  | | Mycosphaerella sp. EHS201 | | 0 | 0 | 1 | 0 | 0 | 0 | 0 | 0 | 0 | 0 | 0 | 0 |
|  |  | | Mycosphaerella sp. F29 | | 1 | 0 | 0 | 0 | 0 | 0 | 0 | 0 | 0 | 0 | 0 | 0 |
|  | Dothideales | | Aureobasidium pullulans | | 1 | 1 | 0 | 0 | 0 | 0 | 0 | 0 | 0 | 0 | 0 | 0 |
|  | Myriangiales | | Elsinoaceae sp. ZLY-2010b | | 0 | 0 | 0 | 0 | 0 | 1 | 0 | 0 | 0 | 0 | 0 | 0 |
|  | mitosporic Dothideomycetes | | Epicoccum nigrum | | 0 | 0 | 0 | 1 | 0 | 0 | 0 | 0 | 0 | 0 | 0 | 0 |
|  | Mytilinidiales | | Quasiconcha reticulata | | 0 | 0 | 0 | 0 | 0 | 0 | 1 | 0 | 0 | 0 | 0 | 0 |
|  | Pleosporales | | Didymosphaeria futilis | | 0 | 0 | 0 | 0 | 3 | 2 | 0 | 0 | 0 | 0 | 0 | 0 |
|  |  | | Lophiostoma arundinis | | 1 | 0 | 0 | 0 | 0 | 0 | 0 | 0 | 0 | 0 | 0 | 0 |
|  |  | | Lophiostoma caulium | | 0 | 1 | 0 | 0 | 0 | 0 | 0 | 0 | 0 | 0 | 0 | 0 |
|  |  | | Lophiostoma macrostomum | | 1 | 0 | 0 | 0 | 0 | 0 | 0 | 0 | 0 | 0 | 0 | 0 |
|  |  | | Lophiostoma sp. MA 4558 | | 0 | 0 | 1 | 1 | 0 | 0 | 0 | 0 | 0 | 0 | 0 | 0 |
|  |  | | Massarina cisti | | 0 | 0 | 0 | 0 | 0 | 0 | 0 | 1 | 0 | 0 | 0 | 0 |
|  |  | | Saccharicola bicolor | | 0 | 0 | 4 | 3 | 0 | 0 | 0 | 0 | 0 | 0 | 0 | 0 |
|  |  | | Ascochyta hordei | | 0 | 0 | 0 | 0 | 0 | 1 | 0 | 0 | 0 | 0 | 0 | 0 |
|  |  | | Coniothyrium sp. BF62 | | 1 | 2 | 0 | 0 | 0 | 0 | 0 | 0 | 0 | 0 | 0 | 0 |
|  |  | | Paraconiothyrium sp. BLE20 | | 0 | 0 | 0 | 1 | 0 | 0 | 0 | 0 | 0 | 0 | 0 | 0 |
|  |  | | Paraconiothyrium sporulosum | | 8 | 10 | 0 | 0 | 0 | 0 | 0 | 0 | 0 | 0 | 0 | 0 |
|  |  | | Paraphaeosphaeria michotii | | 1 | 2 | 0 | 0 | 0 | 0 | 0 | 0 | 0 | 0 | 0 | 0 |
|  |  | | uncultured Leptosphaeriaceae | | 0 | 0 | 5 | 5 | 0 | 0 | 0 | 0 | 0 | 0 | 0 | 0 |
|  |  | | Leptosphaeria microscopica | | 0 | 0 | 3 | 2 | 0 | 0 | 0 | 0 | 0 | 0 | 0 | 0 |
|  |  | | Leptosphaeria sp. GFI 123 | | 0 | 0 | 2 | 0 | 0 | 0 | 0 | 0 | 0 | 0 | 0 | 0 |
|  |  | | Leptosphaeria sp. L413 | | 1 | 0 | 0 | 0 | 0 | 0 | 0 | 0 | 0 | 0 | 0 | 0 |
|  |  | | Leptosphaeria typharum | | 0 | 1 | 1 | 0 | 0 | 0 | 0 | 0 | 0 | 0 | 0 | 0 |
|  |  | | Paraphoma | | 0 | 0 | 2 | 4 | 0 | 0 | 0 | 0 | 0 | 0 | 0 | 0 |
|  |  | | Neophaeosphaeria filamentosa | | 0 | 0 | 0 | 0 | 0 | 0 | 1 | 0 | 0 | 0 | 0 | 0 |
|  |  | | Phaeosphaeria caricinella | | 0 | 0 | 0 | 1 | 0 | 0 | 0 | 0 | 0 | 0 | 0 | 0 |
|  |  | | Phaeosphaeria eustoma | | 3 | 3 | 0 | 0 | 0 | 0 | 0 | 0 | 0 | 0 | 0 | 0 |
|  |  | | Phaeosphaeria juncophila | | 3 | 6 | 2 | 2 | 0 | 0 | 0 | 0 | 0 | 0 | 0 | 0 |
|  |  | | Phaeosphaeria pontiformis | | 4 | 6 | 0 | 0 | 0 | 0 | 0 | 0 | 0 | 0 | 0 | 0 |
|  |  | | Phaeosphaeria rousseliana | | 0 | 0 | 0 | 0 | 1 | 3 | 0 | 0 | 0 | 0 | 0 | 0 |
|  |  | | Phaeosphaeria sp. CC52/6a-1 | | 0 | 0 | 2 | 2 | 0 | 0 | 0 | 0 | 0 | 0 | 0 | 0 |
|  |  | | Phaeosphaeria sp. LC-03-004 | | 0 | 3 | 0 | 0 | 0 | 0 | 0 | 0 | 0 | 0 | 0 | 0 |
|  |  | | Alternaria helianthi | | 0 | 0 | 0 | 0 | 0 | 1 | 0 | 0 | 0 | 0 | 0 | 0 |
|  |  | | Dendryphiella vinosa | | 0 | 1 | 0 | 0 | 0 | 0 | 0 | 0 | 0 | 0 | 0 | 0 |
|  |  | | Pyrenochaeta sp. 14009 | | 0 | 0 | 0 | 0 | 1 | 1 | 0 | 0 | 0 | 0 | 0 | 0 |
|  |  | | Stagonospora sp. 4/99-5 | | 3 | 6 | 0 | 0 | 0 | 0 | 0 | 0 | 0 | 0 | 0 | 0 |
|  |  | | Pleospora sp. AFTOL 2207 | | 0 | 0 | 0 | 0 | 0 | 0 | 1 | 0 | 0 | 0 | 0 | 0 |
|  |  | | Pleosporales sp. MU-2009-2 | | 6 | 4 | 0 | 0 | 0 | 0 | 0 | 0 | 0 | 0 | 0 | 0 |
|  |  | | Pleosporales sp. OY1507 | | 1 | 0 | 0 | 0 | 0 | 0 | 0 | 0 | 0 | 0 | 0 | 0 |
|  | unclassified Dothideomycetes | | Dothideomycetes sp. 11047 | | 0 | 0 | 1 | 0 | 0 | 0 | 0 | 0 | 0 | 0 | 0 | 0 |
|  | Chaetothyriales | | Capronia pilosella | | 2 | 2 | 0 | 0 | 0 | 0 | 0 | 0 | 0 | 0 | 0 | 0 |
|  |  | | Cladophialophora boppii | | 1 | 0 | 0 | 0 | 0 | 0 | 0 | 0 | 0 | 0 | 0 | 0 |
|  |  | | Cladophialophora proteae | | 1 | 0 | 0 | 0 | 0 | 0 | 0 | 0 | 0 | 0 | 0 | 0 |
|  |  | | Phaeococcomyces nigricans | | 0 | 0 | 0 | 0 | 0 | 0 | 1 | 0 | 0 | 0 | 0 | 0 |
|  | Eurotiales | | Trichocomaceae | | 0 | 0 | 1 | 0 | 0 | 0 | 0 | 0 | 0 | 0 | 0 | 0 |
|  | Onygenales | | Spiromastix warcupii | | 2 | 0 | 0 | 0 | 0 | 0 | 0 | 0 | 0 | 0 | 0 | 0 |
|  | Umbilicariales | | Umbilicaria crustulosa | | 3 | 3 | 0 | 0 | 0 | 0 | 0 | 0 | 0 | 0 | 0 | 0 |
|  | Lecanorales | | Lecanora concolor | | 0 | 0 | 0 | 0 | 0 | 0 | 0 | 1 | 0 | 0 | 0 | 0 |
|  |  | | Hypotrachyna pseudosinuosa | | 0 | 0 | 0 | 0 | 0 | 0 | 1 | 0 | 0 | 0 | 0 | 0 |
|  |  | | Amandinea punctata | | 0 | 0 | 0 | 0 | 0 | 0 | 1 | 1 | 0 | 0 | 0 | 0 |
|  | Peltigerales | | Sticta fuliginosa | | 0 | 0 | 0 | 1 | 0 | 0 | 0 | 0 | 0 | 0 | 0 | 0 |
|  |  | | Nephroma isidiosum | | 6 | 6 | 0 | 0 | 0 | 0 | 0 | 0 | 0 | 0 | 0 | 0 |
|  | Agyriales | | Rimularia psephota | | 0 | 0 | 0 | 0 | 0 | 0 | 0 | 1 | 0 | 0 | 0 | 0 |
|  | Ostropales | | Gyalectaceae | | 0 | 0 | 0 | 0 | 0 | 0 | 1 | 1 | 0 | 0 | 0 | 0 |
|  | Lichinales | | Lichinella iodopulchra | | 0 | 0 | 1 | 0 | 0 | 0 | 0 | 0 | 0 | 0 | 0 | 0 |
|  | Pyxidiophorales | | Pyxidiophora arvernensis | | 0 | 0 | 0 | 0 | 0 | 0 | 2 | 3 | 0 | 0 | 0 | 0 |
|  | Cyttariales | | Cyttaria hookeri | | 0 | 0 | 0 | 0 | 4 | 5 | 0 | 0 | 0 | 0 | 0 | 0 |
|  | Erysiphales | | Phyllactinia | | 0 | 0 | 1 | 0 | 0 | 0 | 0 | 0 | 0 | 0 | 0 | 0 |
|  | Helotiales | | Mollisia dextrinospora | | 0 | 1 | 0 | 0 | 0 | 0 | 0 | 0 | 0 | 0 | 0 | 0 |
|  |  | | Neofabraea malicorticis | | 1 | 0 | 0 | 0 | 0 | 0 | 0 | 0 | 0 | 0 | 0 | 0 |
|  |  | | uncultured Helotiales | | 0 | 0 | 6 | 7 | 0 | 0 | 0 | 0 | 0 | 0 | 0 | 0 |
|  |  | | Ascocoryne cylichnium | | 0 | 0 | 0 | 0 | 0 | 2 | 0 | 0 | 0 | 0 | 0 | 0 |
|  |  | | Ascocoryne sarcoides | | 1 | 0 | 0 | 0 | 0 | 0 | 0 | 0 | 0 | 0 | 0 | 0 |
|  |  | | Bryoglossum gracile | | 0 | 0 | 0 | 0 | 0 | 0 | 0 | 1 | 0 | 0 | 0 | 0 |
|  |  | | Crocicreas sp. F-124,806 | | 0 | 0 | 3 | 1 | 0 | 0 | 0 | 0 | 0 | 0 | 0 | 0 |
|  |  | | Cudoniella sp. ZW-Geo49-Clark | | 0 | 0 | 0 | 0 | 1 | 1 | 0 | 0 | 0 | 0 | 0 | 0 |
|  |  | | Holwaya mucida | | 5 | 7 | 0 | 0 | 0 | 0 | 0 | 0 | 0 | 0 | 0 | 0 |
|  |  | | Hymenoscyphus scutula | | 0 | 0 | 0 | 0 | 0 | 1 | 0 | 0 | 0 | 0 | 0 | 0 |
|  |  | | Articulospora proliferata | | 3 | 4 | 0 | 0 | 0 | 0 | 0 | 0 | 0 | 0 | 0 | 0 |
|  |  | | Articulospora tetracladia | | 1 | 0 | 0 | 0 | 0 | 0 | 0 | 0 | 0 | 0 | 0 | 0 |
|  |  | | Helicodendron conglomeratum | | 0 | 0 | 0 | 0 | 3 | 4 | 0 | 0 | 0 | 0 | 0 | 0 |
|  |  | | Rhizoscyphus | | 0 | 0 | 2 | 2 | 0 | 0 | 0 | 0 | 0 | 0 | 0 | 0 |
|  |  | | Mycoarthris | | 0 | 0 | 3 | 4 | 0 | 0 | 0 | 0 | 0 | 0 | 0 | 0 |
|  |  | | Protoventuria alpina | | 3 | 0 | 0 | 0 | 0 | 0 | 0 | 0 | 0 | 0 | 0 | 0 |
|  |  | | Lachnum sp. FC-2244 | | 1 | 0 | 0 | 0 | 0 | 0 | 0 | 0 | 0 | 0 | 0 | 0 |
|  |  | | Lasiobelonium lonicerae | | 0 | 0 | 0 | 0 | 1 | 1 | 0 | 0 | 0 | 0 | 0 | 0 |
|  |  | | Clathrosphaerina | | 2 | 2 | 0 | 0 | 0 | 0 | 0 | 0 | 0 | 0 | 0 | 0 |
|  |  | | Neobulgaria pura | | 1 | 2 | 0 | 0 | 0 | 3 | 3 | 2 | 0 | 0 | 0 | 0 |
|  |  | | Neobulgaria sp. ICMP 18072 | | 0 | 0 | 0 | 0 | 1 | 0 | 0 | 0 | 0 | 0 | 0 | 0 |
|  |  | | Cadophora fastigiata | | 0 | 1 | 0 | 0 | 0 | 0 | 0 | 0 | 0 | 0 | 0 | 0 |
|  |  | | Cadophora luteo-olivacea | | 7 | 7 | 1 | 0 | 0 | 0 | 0 | 0 | 0 | 0 | 0 | 0 |
|  |  | | Chalara fungorum | | 0 | 0 | 0 | 0 | 0 | 0 | 1 | 0 | 0 | 0 | 0 | 0 |
|  |  | | Chalara hyalina | | 0 | 0 | 0 | 0 | 0 | 3 | 0 | 0 | 0 | 0 | 0 | 0 |
|  |  | | Chalara siamense | | 0 | 0 | 0 | 0 | 0 | 0 | 1 | 2 | 0 | 0 | 0 | 0 |
|  |  | | Phialocephala fortinii | | 0 | 0 | 0 | 0 | 0 | 1 | 0 | 0 | 0 | 0 | 0 | 0 |
|  |  | | Rhizocladosporium argillaceum | | 0 | 0 | 0 | 0 | 0 | 0 | 1 | 1 | 0 | 0 | 0 | 0 |
|  |  | | Varicosporium scoparium | | 0 | 0 | 0 | 0 | 1 | 0 | 0 | 0 | 0 | 0 | 0 | 0 |
|  |  | | Helotiales sp. 1959 | | 1 | 1 | 0 | 1 | 0 | 0 | 0 | 0 | 0 | 0 | 0 | 0 |
|  |  | | Helotiales sp. 859 | | 3 | 2 | 2 | 0 | 0 | 0 | 0 | 0 | 0 | 0 | 0 | 0 |
|  | Leotiomycetes incertae sedis | | Amorphotheca resinae | | 0 | 0 | 0 | 0 | 0 | 1 | 0 | 0 | 0 | 0 | 0 | 0 |
|  |  | | Gymnostellatospora alpina | | 0 | 0 | 0 | 2 | 0 | 0 | 0 | 0 | 0 | 0 | 0 | 0 |
|  |  | | Geomyces | | 5 | 6 | 1 | 0 | 0 | 0 | 0 | 0 | 0 | 0 | 0 | 0 |
|  |  | | Oidiodendron | | 0 | 0 | 0 | 0 | 0 | 1 | 0 | 0 | 0 | 0 | 0 | 0 |
|  |  | | Pseudeurotium bakeri | | 7 | 4 | 5 | 6 | 0 | 0 | 0 | 0 | 0 | 0 | 0 | 0 |
|  |  | | Pseudeurotium sp. 795_2_CY02 | | 0 | 0 | 0 | 0 | 0 | 0 | 1 | 2 | 0 | 0 | 0 | 0 |
|  |  | | Pseudeurotium zonatum | | 0 | 0 | 0 | 0 | 1 | 2 | 0 | 0 | 0 | 0 | 0 | 0 |
|  |  | | Pseudeurotiaceae sp. BC14 | | 0 | 0 | 2 | 0 | 0 | 0 | 0 | 0 | 0 | 0 | 0 | 0 |
|  |  | | Leohumicola minima | | 2 | 5 | 0 | 0 | 0 | 0 | 0 | 0 | 0 | 0 | 0 | 0 |
|  | Rhytismatales | | Lophodermium eucalypti | | 0 | 0 | 0 | 0 | 0 | 0 | 0 | 1 | 0 | 0 | 0 | 0 |
|  | Thelebolales | | Thelebolus sp. G1 | | 0 | 0 | 0 | 0 | 0 | 1 | 0 | 0 | 0 | 0 | 0 | 0 |
|  |  | | Thelebolus stercoreus | | 0 | 0 | 1 | 1 | 0 | 0 | 0 | 0 | 0 | 0 | 0 | 0 |
|  | Hypocreales | | Bionectria compactiuscula | | 0 | 0 | 0 | 0 | 0 | 0 | 1 | 0 | 0 | 0 | 0 | 0 |
|  |  | | Bionectria ochroleuca | | 1 | 0 | 0 | 0 | 0 | 0 | 0 | 0 | 0 | 0 | 0 | 0 |
|  |  | | Ijuhya paraparilis | | 2 | 0 | 1 | 0 | 0 | 0 | 0 | 0 | 0 | 0 | 0 | 0 |
|  |  | | Verrucostoma freycinetiae | | 0 | 1 | 0 | 0 | 0 | 0 | 0 | 0 | 0 | 0 | 0 | 0 |
|  |  | | Tolypocladium cylindrosporum | | 0 | 1 | 0 | 0 | 0 | 0 | 0 | 0 | 0 | 0 | 0 | 0 |
|  |  | | Clavicipitaceae sp. LM336 | | 2 | 2 | 0 | 0 | 0 | 0 | 0 | 0 | 0 | 0 | 0 | 0 |
|  |  | | Cordyceps bassiana | | 2 | 4 | 0 | 1 | 0 | 0 | 0 | 0 | 0 | 0 | 0 | 0 |
|  |  | | Hypocrea lixii | | 0 | 2 | 0 | 0 | 0 | 0 | 0 | 0 | 0 | 0 | 0 | 0 |
|  |  | | Emericellopsis terricola | | 0 | 0 | 0 | 0 | 5 | 6 | 0 | 0 | 0 | 0 | 0 | 0 |
|  |  | | Eucasphaeria capensis | | 1 | 0 | 1 | 0 | 1 | 0 | 0 | 0 | 0 | 0 | 0 | 0 |
|  |  | | Acremonium cucurbitacearum | | 0 | 0 | 0 | 1 | 0 | 0 | 0 | 0 | 0 | 0 | 0 | 0 |
|  |  | | Cephalosporium | | 0 | 0 | 1 | 0 | 0 | 0 | 0 | 0 | 0 | 0 | 0 | 0 |
|  |  | | Fusarium lateritium | | 0 | 0 | 1 | 0 | 0 | 0 | 0 | 0 | 0 | 0 | 0 | 0 |
|  |  | | Fusarium merismoides | | 0 | 0 | 0 | 0 | 0 | 1 | 0 | 0 | 0 | 0 | 0 | 0 |
|  |  | | Fusarium sp. Pr13 | | 1 | 1 | 0 | 0 | 0 | 0 | 0 | 0 | 0 | 0 | 0 | 0 |
|  |  | | Fusarium tricinctum | | 0 | 0 | 0 | 0 | 0 | 0 | 1 | 1 | 0 | 0 | 0 | 0 |
|  |  | | Myrothecium roridum | | 0 | 0 | 0 | 0 | 0 | 1 | 0 | 0 | 0 | 0 | 0 | 0 |
|  |  | | Myrothecium sp. HKC11 | | 0 | 0 | 0 | 1 | 0 | 0 | 0 | 0 | 0 | 0 | 0 | 0 |
|  |  | | Calonectria pyrochroa | | 0 | 0 | 0 | 0 | 0 | 1 | 0 | 1 | 0 | 0 | 0 | 0 |
|  |  | | uncultured Nectriaceae | | 0 | 1 | 0 | 0 | 0 | 0 | 0 | 0 | 0 | 0 | 0 | 0 |
|  |  | | Gibberella | | 1 | 0 | 0 | 0 | 0 | 0 | 0 | 0 | 0 | 0 | 0 | 0 |
|  |  | | Lasionectria mantuana | | 0 | 0 | 0 | 0 | 0 | 0 | 2 | 0 | 0 | 0 | 0 | 0 |
|  |  | | Cylindrium | | 4 | 3 | 0 | 0 | 0 | 0 | 0 | 0 | 0 | 0 | 0 | 0 |
|  |  | | Nectriaceae sp. LM140 | | 0 | 1 | 0 | 0 | 0 | 0 | 0 | 0 | 0 | 0 | 0 | 0 |
|  |  | | Elaphocordyceps sp. 20102666 | | 2 | 1 | 0 | 0 | 0 | 0 | 0 | 0 | 0 | 0 | 0 | 0 |
|  |  | | Hypocreales sp. GMG_PPb3 | | 1 | 0 | 0 | 0 | 0 | 0 | 0 | 0 | 0 | 0 | 0 | 0 |
|  |  | | Hypocreales sp. NRRL 46123 | | 0 | 0 | 0 | 0 | 1 | 0 | 0 | 0 | 0 | 0 | 0 | 0 |
|  | Microascales | | Monodictys | | 0 | 1 | 0 | 0 | 0 | 0 | 0 | 0 | 0 | 0 | 0 | 0 |
|  |  | | Periconia | | 0 | 0 | 1 | 0 | 0 | 1 | 0 | 0 | 0 | 0 | 0 | 0 |
|  |  | | Microascaceae sp. LM278 | | 2 | 1 | 0 | 0 | 0 | 0 | 0 | 0 | 0 | 0 | 0 | 0 |
|  |  | | Gabarnaudia betae | | 6 | 6 | 4 | 3 | 2 | 2 | 0 | 0 | 0 | 0 | 0 | 0 |
|  |  | | Sphaeronaemella fimicola | | 0 | 0 | 0 | 0 | 0 | 0 | 3 | 3 | 0 | 0 | 0 | 0 |
|  |  | | Doratomyces | | 2 | 0 | 0 | 0 | 0 | 0 | 0 | 0 | 0 | 0 | 0 | 0 |
|  | Sordariomycetes incertae sedis | | Phlogicylindrium eucalyptorum | | 2 | 0 | 0 | 0 | 0 | 0 | 0 | 0 | 0 | 0 | 0 | 0 |
|  | Phyllachorales | | Verticillium | | 1 | 3 | 1 | 3 | 0 | 0 | 0 | 0 | 0 | 0 | 0 | 0 |
|  |  | | Plectosphaera eucalypti | | 2 | 1 | 0 | 0 | 0 | 0 | 0 | 0 | 0 | 0 | 0 | 0 |
|  | Glomerellales | | Plectosphaerella cucumerina | | 8 | 6 | 6 | 5 | 0 | 0 | 0 | 0 | 0 | 0 | 0 | 0 |
|  |  | | Plectosphaerella sp. I392 | | 3 | 2 | 0 | 0 | 0 | 0 | 0 | 0 | 0 | 0 | 0 | 0 |
|  | Trichosphaeriales | | Trichosphaeria pilosa | | 0 | 0 | 0 | 0 | 0 | 0 | 0 | 1 | 0 | 0 | 0 | 0 |
|  | Sordariomycetes incertae sedis | | Wallrothiella subiculosa | | 0 | 0 | 0 | 0 | 0 | 0 | 0 | 1 | 0 | 0 | 0 | 0 |
|  | Coniochaetales | | Coniochaeta leucoplaca | | 0 | 0 | 0 | 0 | 0 | 0 | 1 | 0 | 0 | 0 | 0 | 0 |
|  | Diaporthales | | Valsaceae | | 1 | 0 | 0 | 0 | 0 | 0 | 0 | 0 | 0 | 0 | 0 | 0 |
|  | Magnaporthales | | Mycoleptodiscus indicus | | 2 | 2 | 0 | 0 | 0 | 0 | 0 | 0 | 0 | 0 | 0 | 0 |
|  |  | | Phialophora sp. C16 | | 0 | 2 | 0 | 0 | 0 | 0 | 0 | 0 | 0 | 0 | 0 | 0 |
|  |  | | Phialophora sp. L5 | | 1 | 0 | 0 | 0 | 0 | 0 | 0 | 0 | 0 | 0 | 0 | 0 |
|  |  | | Phialophora sp. p3847 | | 1 | 0 | 1 | 0 | 0 | 0 | 0 | 0 | 0 | 0 | 0 | 0 |
|  |  | | Phialophora sp. p3901 | | 0 | 0 | 1 | 0 | 0 | 0 | 0 | 0 | 0 | 0 | 0 | 0 |
|  |  | | Pyricularia cortaderiae | | 1 | 3 | 0 | 0 | 0 | 0 | 0 | 0 | 0 | 0 | 0 | 0 |
|  | Ophiostomatales | | Phialocephala | | 1 | 0 | 0 | 0 | 0 | 0 | 0 | 0 | 0 | 0 | 0 | 0 |
|  |  | | Ophiostoma stenoceras | | 0 | 0 | 0 | 1 | 0 | 0 | 0 | 0 | 0 | 0 | 0 | 0 |
|  | Sordariales | | Chaetomium murorum | | 0 | 0 | 0 | 0 | 0 | 0 | 2 | 0 | 0 | 0 | 0 | 0 |
|  |  | | Chaetomium nigricolor | | 1 | 0 | 0 | 0 | 0 | 0 | 0 | 0 | 0 | 0 | 0 | 0 |
|  |  | | Chaetomium sp. 6/97-38 | | 1 | 0 | 0 | 0 | 0 | 0 | 0 | 0 | 0 | 0 | 0 | 0 |
|  |  | | Apiosordaria otanii | | 0 | 0 | 0 | 1 | 0 | 0 | 0 | 0 | 0 | 0 | 0 | 0 |
|  |  | | Cercophora aff. mirabilis SMH4238 | | 0 | 0 | 0 | 0 | 1 | 1 | 0 | 0 | 0 | 0 | 0 | 0 |
|  |  | | Cercophora ambigua | | 0 | 0 | 0 | 2 | 0 | 0 | 0 | 0 | 0 | 0 | 0 | 0 |
|  |  | | Cercophora areolata | | 2 | 3 | 0 | 1 | 2 | 1 | 0 | 0 | 0 | 0 | 0 | 0 |
|  |  | | Cercophora scortea | | 0 | 0 | 0 | 0 | 0 | 1 | 0 | 0 | 0 | 0 | 0 | 0 |
|  |  | | Lasiosphaeris hispida | | 0 | 1 | 0 | 0 | 0 | 0 | 0 | 0 | 0 | 0 | 0 | 0 |
|  |  | | Podospora appendiculata | | 0 | 1 | 1 | 0 | 0 | 0 | 0 | 0 | 0 | 0 | 0 | 0 |
|  |  | | Podospora conica | | 0 | 0 | 1 | 0 | 0 | 0 | 0 | 0 | 0 | 0 | 0 | 0 |
|  |  | | Podospora curvicolla | | 0 | 0 | 0 | 0 | 0 | 0 | 2 | 1 | 0 | 0 | 0 | 0 |
|  |  | | Podospora decidua | | 1 | 0 | 0 | 1 | 0 | 0 | 0 | 0 | 0 | 0 | 0 | 0 |
|  |  | | Podospora ellisiana | | 0 | 1 | 0 | 0 | 0 | 0 | 0 | 0 | 0 | 0 | 0 | 0 |
|  |  | | Podospora glutinans | | 0 | 1 | 0 | 0 | 0 | 0 | 0 | 0 | 0 | 0 | 0 | 0 |
|  |  | | Podospora pyriformis | | 0 | 0 | 1 | 1 | 0 | 0 | 0 | 0 | 0 | 0 | 0 | 0 |
|  |  | | Podospora tetraspora | | 0 | 0 | 0 | 2 | 0 | 0 | 0 | 0 | 0 | 0 | 0 | 0 |
|  |  | | Schizothecium carpinicola | | 1 | 3 | 0 | 0 | 0 | 0 | 0 | 0 | 0 | 0 | 0 | 0 |
|  |  | | Schizothecium vesticola | | 0 | 0 | 0 | 0 | 2 | 4 | 0 | 0 | 0 | 0 | 0 | 0 |
|  |  | | Triangularia tanzaniensis | | 0 | 0 | 0 | 0 | 1 | 1 | 0 | 0 | 0 | 0 | 0 | 0 |
|  |  | | Zygopleurage zygospora | | 3 | 1 | 0 | 0 | 0 | 0 | 0 | 0 | 0 | 0 | 0 | 0 |
|  |  | | Paecilomyces inflatus | | 1 | 0 | 0 | 0 | 0 | 0 | 0 | 0 | 0 | 0 | 0 | 0 |
|  |  | | Sordariaceae | | 0 | 0 | 0 | 0 | 1 | 0 | 0 | 0 | 0 | 0 | 0 | 0 |
|  | Sordariomycetidae incertae sedis | | Jobellisia rhynchostoma | | 0 | 0 | 0 | 0 | 1 | 0 | 0 | 0 | 0 | 0 | 0 | 0 |
|  | unclassified Sordariomycetes | | Sordariomycetes sp. 11323 | | 0 | 0 | 0 | 0 | 0 | 1 | 0 | 0 | 0 | 0 | 0 | 0 |
|  | Xylariales | | Seimatosporium | | 0 | 0 | 0 | 0 | 0 | 0 | 1 | 0 | 0 | 0 | 0 | 0 |
|  |  | | Truncatella angustata | | 1 | 0 | 0 | 0 | 0 | 0 | 0 | 0 | 0 | 0 | 0 | 0 |
|  |  | | Hyponectria sp. HKUCC 5553 | | 0 | 0 | 0 | 0 | 0 | 0 | 1 | 0 | 0 | 0 | 0 | 0 |
|  |  | | Physalospora scirpi | | 5 | 3 | 1 | 4 | 0 | 0 | 0 | 0 | 0 | 0 | 0 | 0 |
|  |  | | Microdochium phragmitis | | 0 | 0 | 0 | 0 | 0 | 0 | 1 | 0 | 0 | 0 | 0 | 0 |
|  |  | | Anthostomella brabeji | | 0 | 0 | 0 | 0 | 1 | 0 | 0 | 0 | 0 | 0 | 0 | 0 |
|  |  | | Nemania | | 0 | 1 | 0 | 0 | 0 | 0 | 0 | 0 | 0 | 0 | 0 | 0 |
|  | Orbiliales | | Arthrobotrys conoides | | 1 | 0 | 0 | 0 | 0 | 0 | 0 | 0 | 0 | 0 | 0 | 0 |
|  |  | | Dactylaria ampulliformis | | 2 | 0 | 0 | 0 | 0 | 0 | 0 | 0 | 0 | 0 | 0 | 0 |
|  |  | | Dactylaria belliana | | 0 | 0 | 0 | 0 | 0 | 0 | 0 | 1 | 0 | 0 | 0 | 0 |
|  |  | | Dactylella | | 0 | 0 | 0 | 0 | 0 | 0 | 1 | 0 | 0 | 0 | 0 | 0 |
|  |  | | Monacrosporium multiforme | | 0 | 0 | 0 | 0 | 0 | 0 | 1 | 1 | 0 | 0 | 0 | 0 |
|  |  | | Orbilia dorsalia | | 1 | 1 | 0 | 0 | 0 | 0 | 0 | 0 | 0 | 0 | 0 | 0 |
|  | Pezizales | | Ascobolus crenulatus | | 2 | 1 | 0 | 0 | 0 | 0 | 0 | 0 | 0 | 0 | 0 | 0 |
|  |  | | Ascobolus denudatus | | 0 | 0 | 0 | 0 | 0 | 0 | 0 | 1 | 0 | 0 | 0 | 0 |
|  |  | | Ascobolus immersus | | 0 | 1 | 0 | 0 | 0 | 0 | 0 | 0 | 0 | 0 | 0 | 0 |
|  |  | | Ascobolus stercorarius | | 0 | 1 | 0 | 0 | 0 | 0 | 0 | 0 | 0 | 0 | 0 | 0 |
|  |  | | Ascobolaceae sp. 4 YS-2010 | | 3 | 5 | 0 | 0 | 0 | 0 | 0 | 0 | 0 | 0 | 0 | 0 |
|  |  | | Ascodesmis nigricans | | 0 | 0 | 0 | 0 | 1 | 0 | 0 | 0 | 0 | 0 | 0 | 0 |
|  |  | | Helvella maculata | | 0 | 0 | 7 | 6 | 2 | 1 | 6 | 4 | 0 | 0 | 0 | 0 |
|  |  | | Helvella sp. SOC902 | | 1 | 0 | 0 | 0 | 0 | 0 | 0 | 0 | 0 | 0 | 0 | 0 |
|  |  | | Morchella crassipes | | 1 | 0 | 0 | 0 | 0 | 0 | 0 | 0 | 0 | 0 | 0 | 0 |
|  |  | | Imaia gigantea | | 3 | 3 | 0 | 0 | 0 | 0 | 0 | 0 | 0 | 0 | 0 | 0 |
|  |  | | Chromelosporium sp. JMP0018 | | 0 | 1 | 0 | 0 | 0 | 0 | 0 | 0 | 0 | 0 | 0 | 0 |
|  |  | | Peziza alaskana | | 6 | 8 | 0 | 0 | 0 | 1 | 0 | 0 | 0 | 0 | 0 | 0 |
|  |  | | Peziza badia | | 8 | 8 | 6 | 9 | 6 | 7 | 0 | 0 | 0 | 0 | 0 | 0 |
|  |  | | Peziza badiofusca | | 0 | 0 | 0 | 0 | 0 | 0 | 6 | 4 | 0 | 0 | 0 | 0 |
|  |  | | Peziza depressa | | 1 | 0 | 0 | 0 | 0 | 0 | 0 | 0 | 0 | 0 | 0 | 0 |
|  |  | | Peziza infossa | | 0 | 1 | 0 | 0 | 0 | 0 | 0 | 0 | 0 | 0 | 0 | 0 |
|  |  | | Peziza ostracoderma | | 0 | 0 | 6 | 5 | 0 | 0 | 0 | 0 | 0 | 0 | 0 | 0 |
|  |  | | Peziza sp. B276 | | 0 | 0 | 0 | 1 | 0 | 0 | 0 | 0 | 0 | 0 | 0 | 0 |
|  |  | | Peziza subviolacea | | 0 | 0 | 0 | 0 | 0 | 1 | 0 | 0 | 0 | 0 | 0 | 0 |
|  |  | | Ruhlandiella berolinensis | | 0 | 0 | 0 | 0 | 0 | 0 | 0 | 1 | 0 | 0 | 0 | 0 |
|  |  | | Sarcosphaera coronaria | | 0 | 1 | 0 | 0 | 0 | 0 | 0 | 0 | 0 | 0 | 0 | 0 |
|  |  | | Phialea strobilina | | 0 | 0 | 0 | 0 | 0 | 0 | 0 | 2 | 0 | 0 | 0 | 0 |
|  |  | | Genea hispidula | | 2 | 3 | 0 | 0 | 0 | 0 | 0 | 0 | 0 | 0 | 0 | 0 |
|  |  | | Geopora cervina | | 1 | 1 | 0 | 0 | 0 | 0 | 0 | 0 | 0 | 0 | 0 | 0 |
|  |  | | Geopora cf. cervina KH.03.61 | | 0 | 0 | 4 | 7 | 0 | 0 | 2 | 2 | 0 | 0 | 0 | 0 |
|  |  | | Geopora sp. B109 | | 0 | 0 | 0 | 0 | 0 | 1 | 0 | 0 | 0 | 0 | 0 | 0 |
|  |  | | Geopora sp. H VH-22925 | | 7 | 4 | 0 | 0 | 0 | 0 | 0 | 0 | 0 | 0 | 0 | 0 |
|  |  | | Geopora sp. KH.03.109 | | 0 | 0 | 0 | 0 | 0 | 0 | 1 | 0 | 0 | 0 | 0 | 0 |
|  |  | | Geopora sp. KH.03.35 | | 0 | 0 | 0 | 0 | 1 | 2 | 0 | 0 | 0 | 0 | 0 | 0 |
|  |  | | Geopora sp. TAA 192232 | | 1 | 0 | 0 | 0 | 0 | 0 | 0 | 0 | 0 | 0 | 0 | 0 |
|  |  | | Kotlabaea deformis | | 0 | 0 | 0 | 0 | 0 | 0 | 1 | 3 | 0 | 0 | 0 | 0 |
|  |  | | Octospora phagospora | | 0 | 0 | 0 | 0 | 0 | 0 | 1 | 0 | 0 | 0 | 0 | 0 |
|  |  | | Pseudaleuria quinaultiana | | 0 | 0 | 1 | 0 | 0 | 0 | 0 | 0 | 0 | 0 | 0 | 0 |
|  |  | | Pulvinula constellatio | | 0 | 0 | 1 | 2 | 1 | 3 | 2 | 2 | 0 | 0 | 0 | 0 |
|  |  | | Scutellinia colensoi | | 2 | 1 | 0 | 0 | 0 | 0 | 0 | 0 | 0 | 0 | 0 | 0 |
|  |  | | Scutellinia sp. MCKU 020829-12 | | 0 | 0 | 1 | 1 | 0 | 0 | 0 | 0 | 0 | 0 | 0 | 0 |
|  |  | | Tricharina ascophanoides | | 3 | 2 | 0 | 0 | 0 | 0 | 0 | 0 | 0 | 0 | 0 | 0 |
|  |  | | Trichophaea | | 0 | 1 | 0 | 0 | 0 | 0 | 0 | 0 | 0 | 0 | 0 | 0 |
|  |  | | Wilcoxina alaskana | | 2 | 3 | 0 | 0 | 0 | 0 | 0 | 0 | 0 | 0 | 0 | 0 |
|  |  | | Conoplea fusca | | 2 | 2 | 0 | 0 | 0 | 0 | 0 | 0 | 0 | 0 | 0 | 0 |
|  |  | | Pithya cupressina | | 1 | 2 | 0 | 0 | 0 | 0 | 0 | 0 | 0 | 0 | 0 | 0 |
|  |  | | Tuber | | 4 | 3 | 1 | 0 | 0 | 0 | 0 | 0 | 0 | 0 | 0 | 0 |
|  |  | | Pezizomycetes sp. 11237 | | 1 | 0 | 4 | 2 | 0 | 0 | 0 | 0 | 0 | 0 | 0 | 0 |
|  |  | | Pezizomycetes sp. BLD9 | | 0 | 0 | 1 | 0 | 0 | 0 | 0 | 0 | 0 | 0 | 0 | 0 |
|  |  | | Pezizomycetes sp. DC2145 | | 0 | 0 | 1 | 1 | 0 | 0 | 0 | 0 | 0 | 0 | 0 | 0 |
|  |  | | unclassified Pezizomycotina | | 2 | 1 | 0 | 0 | 0 | 0 | 0 | 0 | 0 | 0 | 0 | 0 |
|  | Saccharomycetales | | Scheffersomyces stipitis | | 0 | 0 | 0 | 0 | 0 | 0 | 0 | 1 | 0 | 0 | 0 | 0 |
|  |  | | Galactomyces geotrichum | | 0 | 0 | 0 | 1 | 0 | 0 | 1 | 0 | 0 | 0 | 0 | 0 |
|  |  | | Dipodascaceae sp. LM267 | | 1 | 0 | 0 | 0 | 0 | 0 | 0 | 0 | 0 | 0 | 0 | 0 |
|  |  | | Dipodascopsis tothii | | 0 | 0 | 0 | 0 | 0 | 0 | 0 | 1 | 0 | 0 | 0 | 0 |
|  |  | | Candida auringiensis | | 0 | 0 | 0 | 0 | 0 | 0 | 1 | 0 | 0 | 0 | 0 | 0 |
|  |  | | Candida parapsilosis | | 1 | 0 | 0 | 0 | 0 | 0 | 0 | 0 | 0 | 0 | 0 | 0 |
|  |  | | Candida spandovensis | | 0 | 0 | 0 | 0 | 0 | 0 | 0 | 1 | 0 | 0 | 0 | 0 |
|  |  | | Kluyveromyces lactis | | 0 | 1 | 0 | 0 | 0 | 0 | 0 | 0 | 0 | 0 | 0 | 0 |
|  |  | | Kluyveromyces marxianus | | 2 | 2 | 0 | 0 | 0 | 0 | 0 | 0 | 0 | 0 | 0 | 0 |
|  |  | | Pichia | | 0 | 0 | 0 | 0 | 0 | 0 | 1 | 0 | 0 | 0 | 0 | 0 |
|  |  | | Saccharomycetales sp. LM502 | | 0 | 1 | 0 | 0 | 0 | 0 | 0 | 0 | 0 | 0 | 0 | 0 |
|  | Pneumocystidales | | Pneumocystis carinii | | 0 | 0 | 0 | 0 | 0 | 1 | 0 | 0 | 0 | 0 | 0 | 0 |
|  |  | | Schizosaccharomyces pombe | | 0 | 0 | 0 | 0 | 0 | 0 | 0 | 1 | 0 | 0 | 0 | 0 |
|  | unclassified Ascomycota | | Ascomycota sp. BBC | | 1 | 1 | 0 | 0 | 0 | 0 | 0 | 0 | 0 | 0 | 0 | 0 |
|  |  | | Ascomycota sp. G47 | | 6 | 7 | 0 | 0 | 0 | 0 | 0 | 0 | 0 | 0 | 0 | 0 |
|  |  | | Ascomycota sp. H33 | | 1 | 0 | 0 | 0 | 0 | 0 | 0 | 0 | 0 | 0 | 0 | 0 |
|  |  | | Ascomycota sp. I304 | | 4 | 5 | 2 | 0 | 0 | 0 | 0 | 0 | 0 | 0 | 0 | 0 |
|  |  | | Ascomycota sp. I391 | | 5 | 5 | 0 | 0 | 0 | 0 | 0 | 0 | 0 | 0 | 0 | 0 |
|  |  | | Ascomycota sp. L13 | | 6 | 7 | 0 | 0 | 0 | 0 | 0 | 0 | 0 | 0 | 0 | 0 |
| Basidiomycota | | |  | |  |  |  |  |  |  |  |  |  |  |  |  |
|  | Auriculariales | | Exidiopsis calcea | | 0 | 0 | 0 | 0 | 0 | 0 | 1 | 1 | 0 | 0 | 0 | 0 |
|  |  | | Protodontia piceicola | | 0 | 0 | 0 | 0 | 0 | 1 | 0 | 0 | 0 | 0 | 0 | 0 |
|  |  | | Pseudohydnum gelatinosum | | 0 | 0 | 0 | 0 | 0 | 2 | 0 | 0 | 0 | 0 | 0 | 0 |
|  |  | | Tremiscus helvelloides | | 0 | 1 | 0 | 0 | 0 | 0 | 0 | 0 | 0 | 0 | 0 | 0 |
|  | Cantharellales | | Ceratobasidium sp. AG-A | | 0 | 0 | 1 | 1 | 0 | 0 | 0 | 0 | 0 | 0 | 0 | 0 |
|  |  | | Ceratobasidium sp. AG-G | | 0 | 0 | 0 | 0 | 0 | 0 | 0 | 1 | 0 | 0 | 0 | 0 |
|  |  | | Ceratobasidium sp. FO 38200 | | 0 | 0 | 0 | 0 | 0 | 0 | 0 | 1 | 0 | 0 | 0 | 0 |
|  |  | | Ceratobasidium sp. R20 | | 0 | 0 | 4 | 4 | 0 | 0 | 0 | 0 | 0 | 0 | 0 | 0 |
|  |  | | Thanatephorus cucumeris | | 0 | 0 | 0 | 0 | 0 | 0 | 1 | 0 | 0 | 0 | 0 | 0 |
|  |  | | Clavulina | | 0 | 0 | 0 | 0 | 1 | 0 | 0 | 0 | 0 | 0 | 0 | 0 |
|  |  | | Epulorhiza | | 0 | 0 | 0 | 0 | 1 | 0 | 0 | 0 | 0 | 0 | 0 | 0 |
|  | Corticiales | | Bulbillomyces farinosus | | 0 | 0 | 0 | 1 | 0 | 0 | 0 | 0 | 0 | 0 | 0 | 0 |
|  |  | | Hyphodontia sambuci | | 3 | 3 | 0 | 1 | 0 | 0 | 0 | 0 | 0 | 0 | 0 | 0 |
|  |  | | Hypochniciellum subillaqueatum | | 0 | 0 | 0 | 0 | 4 | 7 | 0 | 0 | 0 | 0 | 0 | 0 |
|  |  | | Hypochnicium karstenii | | 0 | 0 | 0 | 2 | 0 | 0 | 0 | 0 | 0 | 0 | 0 | 0 |
|  |  | | Hypochnicium vellereum | | 0 | 1 | 0 | 0 | 0 | 0 | 0 | 0 | 0 | 0 | 0 | 0 |
|  |  | | Aegerita candida | | 3 | 2 | 0 | 0 | 0 | 0 | 0 | 0 | 0 | 0 | 0 | 0 |
|  |  | | Phlebia uda | | 4 | 0 | 4 | 0 | 0 | 0 | 0 | 0 | 0 | 0 | 0 | 0 |
|  | Agaricomycetes incertae sedis | | Hymenochaetales sp. MO-2010a | | 0 | 2 | 0 | 0 | 0 | 0 | 0 | 0 | 0 | 0 | 0 | 0 |
|  | Polyporales | | Artomyces candelabrus | | 1 | 0 | 0 | 0 | 0 | 0 | 0 | 0 | 0 | 0 | 0 | 0 |
|  |  | | Lentinellus cochleatus | | 0 | 0 | 0 | 0 | 0 | 1 | 0 | 0 | 0 | 0 | 0 | 0 |
|  |  | | Antrodia | | 1 | 1 | 0 | 0 | 0 | 0 | 0 | 0 | 0 | 0 | 0 | 0 |
|  |  | | Oxyporus populinus | | 1 | 1 | 0 | 1 | 0 | 0 | 0 | 0 | 0 | 0 | 0 | 0 |
|  |  | | Oxyporus subpopulinus | | 2 | 2 | 0 | 0 | 0 | 0 | 0 | 0 | 0 | 0 | 0 | 0 |
|  |  | | Postia hibernica | | 0 | 0 | 0 | 1 | 0 | 0 | 0 | 0 | 0 | 0 | 0 | 0 |
|  |  | | Trametes versicolor | | 0 | 1 | 0 | 0 | 0 | 0 | 0 | 0 | 0 | 0 | 0 | 0 |
|  |  | | Ganoderma australe | | 0 | 0 | 0 | 0 | 0 | 0 | 3 | 1 | 0 | 0 | 0 | 0 |
|  |  | | Ganoderma weberianum | | 1 | 0 | 0 | 0 | 0 | 0 | 0 | 0 | 0 | 0 | 0 | 0 |
|  |  | | Perenniporia medulla-panis | | 1 | 1 | 0 | 0 | 0 | 0 | 0 | 0 | 0 | 0 | 0 | 0 |
|  |  | | Polyporus virgatus | | 1 | 0 | 0 | 0 | 0 | 0 | 0 | 0 | 0 | 0 | 0 | 0 |
|  |  | | Pterula echo | | 12 | 7 | 4 | 7 | 2 | 4 | 0 | 0 | 0 | 0 | 0 | 0 |
|  |  | | Pterula epiphylla | | 0 | 0 | 0 | 0 | 0 | 0 | 1 | 5 | 0 | 0 | 0 | 0 |
|  | Russulales | | Hericium coralloides | | 0 | 1 | 0 | 0 | 0 | 0 | 0 | 0 | 0 | 0 | 0 | 0 |
|  |  | | Mycoacia uda | | 1 | 0 | 0 | 0 | 0 | 0 | 0 | 0 | 0 | 0 | 0 | 0 |
|  |  | | Radulodon erikssonii | | 0 | 0 | 0 | 0 | 0 | 0 | 1 | 0 | 0 | 0 | 0 | 0 |
|  | Sebacinales | | Sebacina | | 6 | 4 | 3 | 3 | 0 | 0 | 0 | 0 | 0 | 0 | 0 | 0 |
|  |  | | Bankeraceae | | 0 | 0 | 0 | 1 | 0 | 0 | 0 | 0 | 0 | 0 | 0 | 0 |
|  |  | | Hydnellum mirabile | | 1 | 0 | 0 | 0 | 0 | 0 | 0 | 0 | 0 | 0 | 0 | 0 |
|  |  | | Hydnellum peckii | | 0 | 1 | 0 | 0 | 0 | 0 | 0 | 0 | 0 | 0 | 0 | 0 |
|  |  | | Pseudotomentella sp. K98C35T239 | | 1 | 0 | 0 | 0 | 0 | 0 | 0 | 0 | 0 | 0 | 0 | 0 |
|  |  | | Thelephora | | 0 | 0 | 0 | 0 | 1 | 2 | 0 | 0 | 0 | 0 | 0 | 0 |
|  |  | | Tomentella brunneocystidia | | 1 | 0 | 0 | 0 | 0 | 0 | 0 | 0 | 0 | 0 | 0 | 0 |
|  |  | | Tomentella ellisii | | 0 | 1 | 0 | 0 | 0 | 0 | 0 | 0 | 0 | 0 | 0 | 0 |
|  |  | | Tomentella sp. src822 | | 0 | 0 | 0 | 2 | 0 | 0 | 0 | 0 | 0 | 0 | 0 | 0 |
|  |  | | Tomentella sp. TU 105091 | | 0 | 0 | 0 | 0 | 0 | 0 | 0 | 1 | 0 | 0 | 0 | 0 |
|  |  | | Tomentella sublilacina | | 4 | 2 | 0 | 0 | 0 | 0 | 0 | 0 | 0 | 0 | 0 | 0 |
|  |  | | Typhula incarnata | | 0 | 0 | 2 | 4 | 0 | 0 | 0 | 0 | 0 | 0 | 0 | 0 |
|  |  | | Typhula maritima | | 8 | 11 | 0 | 0 | 0 | 0 | 0 | 0 | 0 | 0 | 0 | 0 |
|  |  | | Typhula sp. Wh-1 | | 4 | 5 | 4 | 3 | 0 | 0 | 0 | 0 | 0 | 0 | 0 | 0 |
|  | Thelephorales | | Thelephorales sp. A.Becerra 06 | | 0 | 0 | 1 | 0 | 0 | 0 | 0 | 0 | 0 | 0 | 0 | 0 |
|  | Trichisporales | | Porpomyces mucidus | | 0 | 0 | 0 | 0 | 0 | 1 | 0 | 0 | 0 | 0 | 0 | 0 |
|  | Agaricales | | Lacrymaria lacrymabunda | | 0 | 0 | 0 | 1 | 0 | 0 | 0 | 0 | 0 | 0 | 0 | 0 |
|  |  | | Micropsalliota sp. Laessoe 6025 | | 0 | 0 | 0 | 0 | 0 | 0 | 2 | 1 | 0 | 0 | 0 | 0 |
|  |  | | Amanita farinosa | | 1 | 0 | 0 | 0 | 0 | 0 | 0 | 0 | 0 | 0 | 0 | 0 |
|  |  | | Clavaria | | 0 | 0 | 1 | 0 | 0 | 0 | 0 | 1 | 0 | 0 | 0 | 0 |
|  |  | | Alnicola | | 1 | 0 | 0 | 0 | 0 | 0 | 0 | 0 | 0 | 0 | 0 | 0 |
|  |  | | Astrosporina viscata | | 2 | 0 | 0 | 0 | 0 | 0 | 0 | 0 | 0 | 0 | 0 | 0 |
|  |  | | Cortinarius bovinus | | 1 | 0 | 1 | 1 | 0 | 0 | 0 | 0 | 0 | 0 | 0 | 0 |
|  |  | | Cortinarius cf. paragaudis OUC97241 | | 1 | 0 | 0 | 0 | 0 | 0 | 0 | 0 | 0 | 0 | 0 | 0 |
|  |  | | Cortinarius limonius | | 0 | 0 | 0 | 1 | 0 | 0 | 0 | 0 | 0 | 0 | 0 | 0 |
|  |  | | Hebeloma | | 2 | 3 | 2 | 1 | 0 | 1 | 1 | 0 | 0 | 0 | 0 | 0 |
|  |  | | Cyphella digitalis | | 0 | 0 | 1 | 0 | 0 | 0 | 0 | 0 | 0 | 0 | 0 | 0 |
|  |  | | Limnoperdon incarnatum | | 0 | 0 | 1 | 1 | 0 | 0 | 0 | 1 | 0 | 0 | 0 | 0 |
|  |  | | Hygrocybe citrinopallida | | 0 | 0 | 1 | 1 | 0 | 0 | 0 | 0 | 0 | 0 | 0 | 0 |
|  |  | | Hygrophorus | | 3 | 0 | 0 | 0 | 0 | 0 | 0 | 0 | 0 | 0 | 0 | 0 |
|  |  | | Hymenogaster | | 1 | 1 | 0 | 0 | 0 | 0 | 0 | 0 | 0 | 0 | 0 | 0 |
|  |  | | Inocybe gymnocarpa | | 0 | 0 | 2 | 4 | 0 | 0 | 0 | 0 | 0 | 0 | 0 | 0 |
|  |  | | Lycoperdon pyriforme | | 4 | 3 | 0 | 0 | 0 | 0 | 0 | 0 | 0 | 0 | 0 | 0 |
|  |  | | Morganella pyriformis | | 0 | 0 | 1 | 5 | 0 | 0 | 0 | 0 | 0 | 0 | 0 | 0 |
|  |  | | Lentinula edodes | | 0 | 1 | 0 | 0 | 0 | 0 | 0 | 0 | 0 | 0 | 0 | 0 |
|  |  | | Pleurotus calyptratus | | 0 | 0 | 1 | 0 | 0 | 0 | 0 | 0 | 0 | 0 | 0 | 0 |
|  |  | | Pluteus cervinus | | 1 | 0 | 0 | 0 | 0 | 0 | 0 | 0 | 0 | 0 | 0 | 0 |
|  |  | | Coprinellus aff. radians 1-2PS | | 2 | 2 | 0 | 0 | 0 | 0 | 0 | 0 | 0 | 0 | 0 | 0 |
|  |  | | Coprinellus micaceus | | 1 | 3 | 1 | 1 | 0 | 0 | 0 | 0 | 0 | 0 | 0 | 0 |
|  |  | | Coprinellus verrucispermus | | 1 | 0 | 0 | 0 | 0 | 0 | 0 | 0 | 0 | 0 | 0 | 0 |
|  |  | | Coprinopsis atramentaria | | 3 | 2 | 2 | 5 | 1 | 1 | 0 | 1 | 0 | 0 | 0 | 0 |
|  |  | | Parasola | | 0 | 0 | 0 | 0 | 0 | 0 | 0 | 2 | 0 | 0 | 0 | 0 |
|  |  | | Psathyrella lutensis | | 0 | 0 | 0 | 0 | 0 | 1 | 0 | 0 | 0 | 0 | 0 | 0 |
|  |  | | Psathyrella pyrotricha | | 0 | 2 | 0 | 0 | 0 | 0 | 0 | 0 | 0 | 0 | 0 | 0 |
|  |  | | Flammula alnicola | | 0 | 1 | 0 | 0 | 0 | 0 | 0 | 0 | 0 | 0 | 0 | 0 |
|  |  | | Galerina | | 2 | 3 | 0 | 0 | 0 | 0 | 0 | 0 | 0 | 0 | 0 | 0 |
|  |  | | Hypholoma tuberosum | | 0 | 0 | 0 | 0 | 1 | 0 | 0 | 0 | 0 | 0 | 0 | 0 |
|  |  | | Pholiota adiposa | | 1 | 2 | 0 | 0 | 0 | 0 | 0 | 0 | 0 | 0 | 0 | 0 |
|  |  | | Pholiota conissans | | 0 | 0 | 1 | 0 | 0 | 0 | 0 | 0 | 0 | 0 | 0 | 0 |
|  |  | | Pholiota squarrosa | | 1 | 0 | 1 | 2 | 0 | 0 | 0 | 0 | 0 | 0 | 0 | 0 |
|  |  | | Psilocybe | | 0 | 0 | 0 | 0 | 0 | 1 | 0 | 0 | 0 | 0 | 0 | 0 |
|  |  | | Calyptella sp. 7110217 | | 0 | 0 | 0 | 1 | 0 | 0 | 0 | 0 | 0 | 0 | 0 | 0 |
|  |  | | Clitocybe subditopoda | | 0 | 0 | 1 | 0 | 0 | 0 | 0 | 0 | 0 | 0 | 0 | 0 |
|  |  | | Gamundia leucophylla | | 0 | 0 | 0 | 1 | 0 | 0 | 0 | 0 | 0 | 0 | 0 | 0 |
|  |  | | Leucopaxillus tricolor | | 2 | 0 | 0 | 0 | 0 | 0 | 0 | 0 | 0 | 0 | 0 | 0 |
|  |  | | Lyophyllum | | 0 | 0 | 3 | 3 | 0 | 0 | 0 | 0 | 0 | 0 | 0 | 0 |
|  |  | | Resupinatus conspersus | | 0 | 0 | 0 | 0 | 1 | 0 | 0 | 0 | 0 | 0 | 0 | 0 |
|  |  | | Tricholoma anatolicum | | 2 | 0 | 0 | 0 | 0 | 0 | 0 | 0 | 0 | 0 | 0 | 0 |
|  |  | | Tricholomataceae sp. RT00032 | | 1 | 0 | 0 | 0 | 0 | 0 | 0 | 0 | 0 | 0 | 0 | 0 |
|  | Boletales | | Xerocomus illudens | | 0 | 0 | 0 | 0 | 0 | 0 | 0 | 1 | 0 | 0 | 0 | 0 |
|  |  | | Alpova diplophloeus | | 0 | 0 | 0 | 0 | 1 | 0 | 1 | 0 | 0 | 0 | 0 | 0 |
|  |  | | Suillus brevipes | | 2 | 3 | 0 | 0 | 0 | 0 | 0 | 0 | 0 | 0 | 0 | 0 |
|  |  | | Suillus grevillei | | 0 | 0 | 0 | 0 | 0 | 0 | 0 | 1 | 0 | 0 | 0 | 0 |
|  | Gomphales | | Lentaria albovinacea | | 0 | 0 | 0 | 0 | 4 | 4 | 4 | 6 | 0 | 0 | 0 | 0 |
|  | Hysterangiales | | Hysterangium | | 1 | 1 | 0 | 0 | 0 | 0 | 0 | 0 | 0 | 0 | 0 | 0 |
|  | Agaricomycotina | | Rhizoctonia sp. Eab-F7 | | 1 | 1 | 0 | 0 | 0 | 0 | 0 | 0 | 0 | 0 | 0 | 0 |
|  | Cystofilobasidiales | | Cystofilobasidium | | 1 | 0 | 1 | 3 | 0 | 0 | 2 | 0 | 0 | 0 | 0 | 0 |
|  |  | | Mrakia frigida | | 0 | 0 | 0 | 0 | 0 | 0 | 0 | 2 | 0 | 0 | 0 | 0 |
|  |  | | Xanthophyllomyces sp. MIC-CONC-2006-762 | | 0 | 0 | 0 | 0 | 1 | 0 | 0 | 0 | 0 | 0 | 0 | 0 |
|  |  | | Cryptococcus macerans | | 3 | 5 | 0 | 0 | 0 | 0 | 0 | 0 | 0 | 0 | 0 | 0 |
|  |  | | Itersonilia perplexans | | 6 | 9 | 3 | 4 | 0 | 0 | 1 | 3 | 0 | 0 | 0 | 0 |
|  |  | | Mrakiella aquatica | | 0 | 0 | 2 | 2 | 0 | 0 | 0 | 0 | 0 | 0 | 0 | 0 |
|  |  | | Udeniomyces pannonicus | | 2 | 2 | 2 | 1 | 0 | 0 | 0 | 0 | 0 | 0 | 0 | 0 |
|  | Filobasidiales | | Cryptococcus gastricus | | 1 | 0 | 0 | 0 | 0 | 0 | 3 | 4 | 0 | 0 | 0 | 0 |
|  |  | | Cryptococcus magnus | | 0 | 0 | 0 | 0 | 0 | 0 | 0 | 1 | 0 | 0 | 0 | 0 |
|  |  | | Cryptococcus sp. CRUB 1154 | | 0 | 0 | 2 | 0 | 0 | 0 | 0 | 0 | 0 | 0 | 0 | 0 |
|  |  | | Cryptococcus sp. JJP-2009a | | 4 | 3 | 0 | 0 | 0 | 0 | 0 | 0 | 0 | 0 | 0 | 0 |
|  |  | | Cryptococcus sp. YKS 2004 | | 3 | 4 | 0 | 0 | 0 | 0 | 0 | 0 | 0 | 0 | 0 | 0 |
|  |  | | Bullera globispora | | 6 | 5 | 4 | 5 | 0 | 0 | 0 | 0 | 0 | 0 | 0 | 0 |
|  |  | | Cryptococcus curvatus | | 0 | 3 | 1 | 2 | 0 | 0 | 0 | 0 | 0 | 0 | 0 | 0 |
|  |  | | Cryptococcus fragicola | | 2 | 0 | 0 | 0 | 0 | 0 | 0 | 0 | 0 | 0 | 0 | 0 |
|  |  | | Cryptococcus sp. BF108 | | 2 | 0 | 0 | 0 | 0 | 0 | 0 | 0 | 0 | 0 | 0 | 0 |
|  |  | | Cryptococcus sp. CBS 681.93 | | 0 | 0 | 0 | 0 | 0 | 0 | 8 | 8 | 0 | 0 | 0 | 0 |
|  |  | | Cryptococcus sp. ZIM 2300 | | 0 | 0 | 0 | 0 | 1 | 1 | 0 | 0 | 0 | 0 | 0 | 0 |
|  |  | | Cryptococcus tephrensis | | 12 | 15 | 0 | 0 | 0 | 0 | 0 | 0 | 0 | 0 | 0 | 0 |
|  |  | | Dioszegia fristingensis | | 4 | 4 | 2 | 2 | 0 | 0 | 0 | 0 | 0 | 0 | 0 | 0 |
|  |  | | Dioszegia hungarica | | 5 | 3 | 3 | 6 | 0 | 0 | 0 | 0 | 0 | 0 | 0 | 0 |
|  |  | | Trichosporon coprophilum | | 0 | 0 | 0 | 0 | 1 | 1 | 0 | 0 | 0 | 0 | 0 | 0 |
|  |  | | Trichosporon debeurmannianum | | 0 | 0 | 1 | 1 | 0 | 0 | 0 | 0 | 0 | 0 | 0 | 0 |
|  |  | | Bulleribasidium oberjochense | | 1 | 0 | 0 | 0 | 0 | 0 | 0 | 0 | 0 | 0 | 0 | 0 |
|  | Holtermanniales | | Holtermannia corniformis | | 0 | 1 | 0 | 0 | 0 | 0 | 0 | 0 | 0 | 0 | 0 | 0 |
|  | Wallemiales | | Wallemia | | 0 | 0 | 0 | 0 | 0 | 0 | 1 | 0 | 0 | 0 | 0 | 0 |
|  | Agaricostilbomycetes incertae sedis | | Rhodotorula | | 0 | 0 | 0 | 1 | 0 | 0 | 0 | 0 | 0 | 0 | 0 | 0 |
|  |  | | Bensingtonia changbaiensis | | 2 | 3 | 1 | 1 | 0 | 0 | 0 | 0 | 0 | 0 | 0 | 0 |
|  | Atractiellales | | Atractiella solani | | 0 | 0 | 1 | 1 | 0 | 0 | 0 | 0 | 0 | 0 | 0 | 0 |
|  | Erythrobasidiales | | Erythrobasidium hasegawianum | | 0 | 0 | 4 | 0 | 0 | 0 | 3 | 0 | 0 | 0 | 0 | 0 |
|  |  | | Occultifur | | 1 | 0 | 0 | 0 | 0 | 0 | 0 | 0 | 0 | 0 | 0 | 0 |
|  |  | | Rhodotorula lamellibrachiae | | 0 | 0 | 0 | 0 | 0 | 1 | 0 | 0 | 0 | 0 | 0 | 0 |
|  |  | | Rhodotorula sp. 5-19 | | 0 | 0 | 0 | 1 | 0 | 0 | 0 | 0 | 0 | 0 | 0 | 0 |
|  |  | | Sporobolomyces bischofiae | | 0 | 0 | 0 | 0 | 1 | 1 | 0 | 0 | 0 | 0 | 0 | 0 |
|  |  | | Sporobolomyces syzygii | | 0 | 0 | 0 | 0 | 1 | 1 | 0 | 0 | 0 | 0 | 0 | 0 |
|  |  | | Sporobolomyces yunnanensis | | 0 | 0 | 1 | 0 | 0 | 0 | 0 | 0 | 0 | 0 | 0 | 0 |
|  |  | | Erythrobasidium clade sp. LM681 | | 4 | 0 | 0 | 0 | 0 | 0 | 0 | 0 | 0 | 0 | 0 | 0 |
|  | Leucosporidiales | | Leucosporidium antarcticum | | 0 | 0 | 0 | 0 | 0 | 1 | 1 | 0 | 0 | 0 | 0 | 0 |
|  |  | | Leucosporidium scottii | | 0 | 0 | 0 | 0 | 0 | 0 | 1 | 1 | 0 | 0 | 0 | 0 |
|  |  | | Leucosporidium sp. AY30 | | 3 | 3 | 0 | 0 | 0 | 0 | 0 | 0 | 0 | 0 | 0 | 0 |
|  |  | | Antarctic yeast CBS 8927 | | 1 | 0 | 0 | 0 | 0 | 0 | 0 | 0 | 0 | 0 | 0 | 0 |
|  | Microbotryomycetes incertae sedis | | Kriegeria eriophori | | 3 | 2 | 1 | 0 | 1 | 0 | 0 | 0 | 0 | 0 | 0 | 0 |
|  |  | | Rhodotorula psychrophenolica | | 0 | 0 | 0 | 0 | 0 | 1 | 0 | 0 | 0 | 0 | 0 | 0 |
|  |  | | Zymoxenogloea | | 2 | 5 | 4 | 5 | 0 | 0 | 0 | 0 | 0 | 0 | 0 | 0 |
|  | Sporidiobolales | | Sporobolomyces sp. JJP-2009a | | 2 | 1 | 0 | 0 | 0 | 0 | 0 | 0 | 0 | 0 | 0 | 0 |
|  |  | | Rhodosporidium fluviale | | 3 | 0 | 3 | 1 | 0 | 0 | 1 | 0 | 0 | 0 | 0 | 0 |
|  |  | | Sporidiobolales sp. LM524 | | 7 | 1 | 0 | 0 | 0 | 0 | 0 | 0 | 0 | 0 | 0 | 0 |
|  | Platygloeales | | Eocronartium muscicola | | 0 | 0 | 0 | 0 | 2 | 2 | 15 | 10 | 0 | 0 | 0 | 0 |
|  |  | | Jola cf. javensis DJM739 | | 0 | 0 | 0 | 0 | 0 | 0 | 0 | 1 | 0 | 0 | 0 | 0 |
|  |  | | Platygloea disciformis | | 20 | 16 | 5 | 5 | 0 | 0 | 0 | 0 | 0 | 0 | 0 | 0 |
|  | Pucciniales | | Chrysomyxa arctostaphyli | | 0 | 0 | 5 | 6 | 0 | 0 | 0 | 0 | 0 | 0 | 0 | 0 |
|  |  | | Phakopsora pachyrhizi | | 1 | 0 | 0 | 0 | 0 | 0 | 0 | 0 | 0 | 0 | 0 | 0 |
|  |  | | Pileolaria terebinthi | | 3 | 4 | 0 | 0 | 0 | 0 | 0 | 0 | 0 | 0 | 0 | 0 |
|  |  | | Gymnosporangium | | 0 | 0 | 0 | 0 | 0 | 1 | 0 | 0 | 0 | 0 | 0 | 0 |
|  |  | | Puccinia allii | | 1 | 0 | 0 | 0 | 0 | 0 | 0 | 0 | 0 | 0 | 0 | 0 |
|  |  | | Ravenelia havanensis | | 0 | 0 | 0 | 0 | 0 | 1 | 0 | 0 | 0 | 0 | 0 | 0 |
|  |  | | Tranzschelia | | 3 | 4 | 0 | 0 | 0 | 0 | 0 | 0 | 0 | 0 | 0 | 0 |
|  | Septobasidiales | | Auriculoscypha anacardiicola | | 0 | 0 | 0 | 0 | 4 | 3 | 0 | 0 | 0 | 0 | 0 | 0 |
|  | unclassified Basidiomycota | | Basidiomycota sp. I7 | | 1 | 0 | 0 | 0 | 0 | 0 | 0 | 0 | 0 | 0 | 0 | 0 |
|  |  | | Basidiomycota sp. L61 | | 0 | 1 | 0 | 0 | 0 | 0 | 0 | 0 | 0 | 0 | 0 | 0 |
|  |  | | oat root associated basidiomycete 00012 | | 1 | 1 | 0 | 0 | 0 | 0 | 0 | 0 | 0 | 0 | 0 | 0 |
|  |  | | oat root associated basidiomycete 00026 | | 1 | 1 | 0 | 0 | 0 | 0 | 0 | 0 | 0 | 0 | 0 | 0 |
|  | Doassansiales | | Rhamphospora nymphaeae | | 0 | 0 | 0 | 0 | 0 | 0 | 0 | 1 | 0 | 0 | 0 | 0 |
|  | Entylomatales | | Tilletiopsis washingtonensis | | 3 | 4 | 3 | 4 | 0 | 1 | 2 | 1 | 0 | 0 | 0 | 0 |
|  |  | | Tilletiopsis minor | | 0 | 1 | 0 | 0 | 0 | 0 | 0 | 0 | 0 | 0 | 0 | 0 |
|  | Malasseziales | | Malassezia restricta | | 4 | 0 | 0 | 0 | 0 | 0 | 0 | 0 | 0 | 0 | 0 | 0 |
|  |  | | Malassezia sp. LCP-2008a | | 1 | 3 | 0 | 0 | 0 | 0 | 0 | 0 | 0 | 0 | 0 | 0 |
|  | Urocystales | | Thecaphora amaranthi | | 3 | 2 | 0 | 0 | 0 | 0 | 0 | 0 | 0 | 0 | 0 | 0 |
|  | Ustilaginales | | Ustilaginaceae | | 0 | 0 | 0 | 0 | 2 | 0 | 3 | 0 | 0 | 0 | 0 | 0 |
| Basal fungal lineages | | |  | |  |  |  |  |  |  |  |  |  |  |  |  |
|  | | Entomophthorales | Basidiobolus meristosporus | | 0 | 0 | 0 | 0 | 0 | 0 | 0 | 1 | 0 | 0 | 0 | 0 |
|  | |  | Schizangiella | | 0 | 1 | 0 | 0 | 0 | 0 | 2 | 0 | 0 | 0 | 0 | 0 |
|  | |  | Entomophthoraceae sp. ARSEF 7942 | | 0 | 0 | 0 | 0 | 0 | 0 | 1 | 1 | 0 | 0 | 0 | 0 |
|  | | Kickxellales | Coemansia reversa | | 0 | 0 | 4 | 3 | 0 | 0 | 0 | 0 | 0 | 0 | 0 | 0 |
|  | | Mortierellales | Mortierella elongata | | 2 | 3 | 0 | 0 | 0 | 0 | 0 | 0 | 0 | 0 | 0 | 0 |
|  | |  | Mortierella fimbricystis | | 0 | 0 | 0 | 3 | 0 | 0 | 0 | 0 | 0 | 0 | 0 | 0 |
|  | |  | Mortierella globalpina | | 0 | 0 | 0 | 0 | 0 | 1 | 0 | 0 | 0 | 0 | 0 | 0 |
|  | |  | Mortierella indohii | | 0 | 0 | 3 | 3 | 0 | 0 | 0 | 0 | 0 | 0 | 0 | 0 |
|  | |  | Mortierella sp. MS-6 | | 0 | 0 | 0 | 0 | 1 | 1 | 1 | 0 | 0 | 0 | 0 | 0 |
|  | |  | Mortierella verticillata | | 0 | 0 | 0 | 0 | 1 | 0 | 2 | 3 | 0 | 0 | 0 | 0 |
|  | |  | Mortierellaceae sp. LN07-7-4 | | 0 | 0 | 2 | 0 | 0 | 0 | 0 | 0 | 0 | 0 | 0 | 0 |
|  | | Mucorales | Gongronella | | 1 | 0 | 0 | 0 | 0 | 0 | 0 | 0 | 0 | 0 | 0 | 0 |
|  | |  | Mucor hiemalis | | 1 | 0 | 0 | 0 | 0 | 0 | 0 | 0 | 0 | 0 | 0 | 0 |
|  | |  | Rhizomucor | | 0 | 1 | 0 | 0 | 0 | 0 | 0 | 0 | 0 | 0 | 0 | 0 |
|  | |  | Rhizopus oryzae | | 1 | 0 | 0 | 1 | 0 | 0 | 0 | 0 | 0 | 0 | 0 | 0 |
|  | |  | Rhizopus sexualis | | 1 | 1 | 0 | 0 | 0 | 0 | 0 | 0 | 0 | 0 | 0 | 0 |
|  | |  | Umbelopsis ramanniana | | 0 | 0 | 4 | 3 | 0 | 0 | 0 | 0 | 0 | 0 | 0 | 0 |
|  | | Zoopagales | Rhopalomyces elegans | | 0 | 0 | 0 | 0 | 0 | 0 | 1 | 0 | 0 | 0 | 0 | 0 |
|  | |  | Piptocephalidaceae | | 1 | 0 | 0 | 0 | 0 | 0 | 3 | 2 | 0 | 0 | 0 | 0 |
| Glomeromycota | | |  | |  |  |  |  |  |  |  |  |  |  |  |  |
|  | | Archaeosporales | Archaeospora | | 5 | 2 | 2 | 3 | 0 | 0 | 0 | 0 | 0 | 0 | 0 | 0 |
|  | | Diversisporales | Acaulospora sp. ZS2005 | | 1 | 0 | 0 | 0 | 0 | 0 | 0 | 0 | 0 | 0 | 0 | 0 |
|  | |  | Entrophospora schenckii | | 1 | 0 | 0 | 0 | 0 | 0 | 0 | 0 | 0 | 0 | 0 | 0 |
|  | |  | Diversispora celata | | 0 | 0 | 0 | 0 | 0 | 0 | 3 | 3 | 0 | 0 | 0 | 0 |
|  | |  | Diversispora sp. AS-2007a | | 5 | 3 | 0 | 0 | 0 | 0 | 0 | 0 | 0 | 0 | 0 | 0 |
|  | |  | Scutellospora gilmorei | | 0 | 0 | 0 | 0 | 1 | 0 | 0 | 0 | 0 | 0 | 0 | 0 |
|  | | Glomerales | Glomus claroideum | | 0 | 1 | 0 | 0 | 0 | 0 | 0 | 0 | 0 | 0 | 0 | 0 |
|  | |  | Glomus drummondi | | 1 | 0 | 0 | 0 | 0 | 0 | 0 | 0 | 0 | 0 | 0 | 0 |
|  | |  | Glomus intraradices | | 2 | 2 | 0 | 0 | 0 | 0 | 0 | 0 | 0 | 0 | 0 | 0 |
|  | |  | Glomus mosseae | | 4 | 4 | 3 | 4 | 0 | 0 | 1 | 0 | 0 | 0 | 0 | 0 |
|  | |  | Glomus sp. 4.1 | | 0 | 0 | 0 | 1 | 0 | 0 | 0 | 0 | 0 | 0 | 0 | 0 |
|  | |  | Glomus sp. NB101 | | 0 | 0 | 1 | 0 | 0 | 0 | 0 | 0 | 0 | 0 | 0 | 0 |
|  | |  | Glomus sp. W3349 | | 1 | 0 | 1 | 1 | 0 | 0 | 0 | 0 | 0 | 0 | 0 | 0 |
|  | |  | Glomus versiforme | | 16 | 14 | 5 | 7 | 1 | 1 | 0 | 0 | 0 | 0 | 0 | 0 |
|  | | Paraglomerales | Paraglomus laccatum | | 0 | 0 | 0 | 0 | 1 | 0 | 0 | 0 | 0 | 0 | 0 | 0 |
|  | |  | Paraglomus occultum | | 0 | 0 | 0 | 0 | 0 | 0 | 1 | 1 | 0 | 0 | 0 | 0 |
| Neocallimastigomycota | | |  | |  |  |  |  |  |  |  |  |  |  |  |  |
|  | | Neocallimastigales | Anaeromyces sp. JB-1999 | | 0 | 1 | 0 | 0 | 0 | 0 | 0 | 0 | 0 | 0 | 0 | 0 |
|  | |  | Neocallimastix sp. GE13 | | 0 | 0 | 0 | 0 | 1 | 2 | 0 | 0 | 0 | 0 | 0 | 0 |
| Fungi/Metazoa incertae sedis | | |  | |  |  |  |  |  |  |  |  |  |  |  |  |
|  | | Ichthyosporea | Sphaerothecum destruens | | 1 | 0 | 4 | 3 | 0 | 0 | 0 | 0 | 0 | 0 | 0 | 0 |
|  | |  | Anurofeca sp. LAH-2003 | | 3 | 7 | 0 | 0 | 0 | 0 | 0 | 0 | 0 | 0 | 0 | 0 |
|  | |  | Ichthyophonida sp. fragrantissima | | 1 | 1 | 0 | 3 | 0 | 0 | 0 | 0 | 0 | 0 | 0 | 0 |
| Platyhelminthes | | |  | |  |  |  |  |  |  |  |  |  |  |  |  |
|  | | Cyclophyllidea | Anoplocephalidae | | 0 | 0 | 0 | 0 | 1 | 0 | 0 | 0 | 0 | 0 | 0 | 0 |
|  | | Cichlidogyridae | Cichlidogyrus sp. 2 XW-2006 | | 6 | 2 | 0 | 0 | 0 | 0 | 0 | 0 | 0 | 0 | 0 | 0 |
| Echinodermata | |  |  | |  |  |  |  |  |  |  |  |  |  |  |  |
|  | | Valvatida | Nectria | | 0 | 0 | 0 | 0 | 0 | 0 | 1 | 0 | 0 | 0 | 0 | 0 |
|  | | Phyllodocida | Perinereis | | 0 | 0 | 1 | 0 | 0 | 0 | 0 | 0 | 0 | 0 | 0 | 0 |
| Mollusca | |  |  | |  |  |  |  |  |  |  |  |  |  |  |  |
|  | | Myoida | Teredinidae | | 0 | 0 | 0 | 0 | 0 | 0 | 1 | 1 | 0 | 0 | 0 | 0 |
|  | | Solemyoida | Solemya velum | | 0 | 0 | 0 | 0 | 1 | 0 | 0 | 0 | 0 | 0 | 0 | 0 |
| Arthropoda | |  |  | |  |  |  |  |  |  |  |  |  |  |  |  |
|  | | Mesostigmata | Dermanyssus sp. LB18 | | 0 | 0 | 1 | 0 | 0 | 0 | 0 | 0 | 0 | 0 | 0 | 0 |
|  | |  | Phorytocarpais fimetorum | | 0 | 0 | 0 | 0 | 0 | 0 | 0 | 1 | 0 | 0 | 0 | 0 |
|  | | Araneae | Entelegynae | | 0 | 0 | 0 | 0 | 0 | 0 | 0 | 1 | 0 | 0 | 0 | 0 |
|  | | Poecilostomatoida | Oncaea | | 0 | 0 | 0 | 0 | 0 | 1 | 0 | 0 | 0 | 0 | 0 | 0 |
| Tardigrada | |  |  | |  |  |  |  |  |  |  |  |  |  |  |  |
|  | | Arthrotardigrada | Stygarctus sp. AJ-2010 | | 0 | 0 | 0 | 0 | 0 | 0 | 2 | 3 | 0 | 0 | 0 | 0 |
| Gastrotricha | |  |  | |  |  |  |  |  |  |  |  |  |  |  |  |
|  | | Macrodasyida | Dactylopodola | | 0 | 0 | 0 | 0 | 0 | 0 | 1 | 0 | 0 | 0 | 0 | 0 |
| Nematoda | |  |  | |  |  |  |  |  |  |  |  |  |  |  |  |
|  | | Araeolaimida | Plectus aquatilis | | 0 | 0 | 0 | 0 | 2 | 3 | 1 | 0 | 0 | 0 | 0 | 0 |
|  | | Monhysterida | Geomonhystera | | 0 | 0 | 0 | 0 | 0 | 0 | 0 | 1 | 0 | 0 | 0 | 0 |
|  | | Oxyurida | Passalurus ambiguus | | 0 | 0 | 1 | 2 | 0 | 0 | 0 | 0 | 0 | 0 | 0 | 0 |
|  | | Rhabditida | Bunonema reticulatum | | 0 | 0 | 0 | 0 | 0 | 1 | 0 | 0 | 0 | 0 | 0 | 0 |
|  | |  | Acrobeloides | | 0 | 0 | 0 | 0 | 0 | 0 | 0 | 1 | 0 | 0 | 0 | 0 |
|  | |  | Eucephalobus sp. JB-55 | | 0 | 0 | 0 | 0 | 0 | 0 | 1 | 0 | 0 | 0 | 0 | 0 |
|  | |  | Heterocephalobellus sp. JB-8 | | 0 | 0 | 0 | 0 | 1 | 0 | 0 | 0 | 0 | 0 | 0 | 0 |
|  | |  | Pseudacrobeles | | 0 | 0 | 0 | 0 | 1 | 1 | 0 | 0 | 0 | 0 | 0 | 0 |
|  | |  | Myolaimus sp. RGD233 | | 0 | 0 | 0 | 0 | 0 | 0 | 1 | 1 | 0 | 0 | 0 | 0 |
|  | |  | unclassified Rhabditida | | 0 | 0 | 0 | 0 | 0 | 0 | 1 | 0 | 0 | 0 | 0 | 0 |
|  | | Tylenchida | Aphelenchus sp. SAN-2005 | | 0 | 0 | 0 | 0 | 0 | 0 | 3 | 0 | 0 | 0 | 0 | 0 |
|  | |  | Aphelenchoides fragariae | | 0 | 0 | 0 | 0 | 0 | 0 | 3 | 2 | 0 | 0 | 0 | 0 |
|  | |  | Laimaphelenchus sp. RGD636L | | 0 | 0 | 0 | 0 | 0 | 0 | 0 | 1 | 0 | 0 | 0 | 0 |
|  | |  | Deladenus siricidicola | | 0 | 0 | 0 | 0 | 0 | 0 | 1 | 3 | 0 | 0 | 0 | 0 |
|  | |  | Mesocriconema xenoplax | | 0 | 0 | 0 | 0 | 0 | 0 | 1 | 0 | 0 | 0 | 0 | 0 |
|  | |  | Hemicycliophora conida | | 0 | 0 | 0 | 0 | 0 | 0 | 1 | 1 | 0 | 0 | 0 | 0 |
|  | |  | Ditylenchus halictus | | 0 | 0 | 0 | 0 | 0 | 0 | 0 | 1 | 0 | 0 | 0 | 0 |
|  | |  | Nagelus leptus | | 0 | 0 | 0 | 0 | 0 | 0 | 1 | 2 | 0 | 0 | 0 | 0 |
|  | |  | Amplimerlinius icarus | | 0 | 0 | 0 | 0 | 0 | 0 | 3 | 2 | 0 | 0 | 0 | 0 |
|  | |  | Globodera | | 0 | 0 | 0 | 0 | 2 | 3 | 0 | 0 | 0 | 0 | 0 | 0 |
|  | |  | Hoplolaimus concaudajuvencus | | 0 | 0 | 0 | 0 | 0 | 1 | 0 | 0 | 0 | 0 | 0 | 0 |
|  | |  | Hoplolaimus galeatus | | 0 | 0 | 0 | 0 | 1 | 4 | 0 | 0 | 0 | 0 | 0 | 0 |
|  | |  | Hoplolaimus magnistylus | | 0 | 0 | 0 | 0 | 0 | 1 | 0 | 0 | 0 | 0 | 0 | 0 |
|  | |  | Scutellonema bradys | | 0 | 0 | 0 | 0 | 1 | 2 | 0 | 0 | 0 | 0 | 0 | 0 |
|  | |  | Coslenchus costatus | | 0 | 0 | 0 | 0 | 1 | 0 | 4 | 5 | 0 | 0 | 0 | 0 |
|  | |  | Psilenchus sp. USA CA9 | | 0 | 0 | 0 | 0 | 0 | 0 | 1 | 3 | 0 | 0 | 0 | 0 |
|  | | Enoplida | Anoplostoma sp. BUS21 | | 0 | 0 | 0 | 0 | 0 | 0 | 1 | 0 | 0 | 0 | 0 | 0 |
|  | |  | Tripylella sp. ZQZ-2010a | | 0 | 0 | 0 | 0 | 0 | 0 | 1 | 0 | 0 | 0 | 0 | 0 |
|  | |  | Trichodorus nanjingensis | | 0 | 0 | 0 | 0 | 0 | 0 | 0 | 1 | 0 | 0 | 0 | 0 |
| Rotifera | |  |  | |  |  |  |  |  |  |  |  |  |  |  |  |
|  | | Ploimida | Brachionus rotundiformis | | 1 | 1 | 0 | 0 | 0 | 0 | 0 | 0 | 0 | 0 | 0 | 0 |
| Cnidaria | |  |  | |  |  |  |  |  |  |  |  |  |  |  |  |
|  | | Hydroida | Symplectoscyphus turgidus | | 0 | 0 | 0 | 0 | 0 | 0 | 2 | 1 | 0 | 0 | 0 | 0 |
|  | | Leucosolenida | Sycon capricorn | | 8 | 6 | 0 | 0 | 0 | 0 | 0 | 0 | 0 | 0 | 0 | 0 |
| Katablepharidophyta | | |  | |  |  |  |  |  |  |  |  |  |  |  |  |
|  | |  | Katablepharidaceae | | 1 | 0 | 0 | 0 | 0 | 0 | 0 | 0 | 0 | 0 | 0 | 0 |
| Rhizaria | |  |  | |  |  |  |  |  |  |  |  |  |  |  |  |
|  | | Cercomonadida | Cercomonas celer | | 0 | 0 | 0 | 3 | 0 | 0 | 0 | 0 | 0 | 0 | 0 | 0 |
|  | |  | Cercomonas vacuolata | | 0 | 0 | 0 | 1 | 0 | 0 | 0 | 0 | 0 | 0 | 0 | 0 |
|  | |  | Eocercomonas sp. 12VMO | | 0 | 1 | 0 | 0 | 0 | 0 | 0 | 0 | 0 | 0 | 0 | 0 |
|  | |  | Eocercomonas sp. PT208-21 | | 1 | 2 | 0 | 0 | 0 | 0 | 0 | 0 | 0 | 0 | 0 | 0 |
|  | |  | Eocercomonas tribula | | 0 | 0 | 1 | 0 | 0 | 0 | 0 | 0 | 0 | 0 | 0 | 0 |
|  | | Plasmodiophorida | Plasmodiophoridae | | 1 | 0 | 0 | 0 | 0 | 0 | 0 | 0 | 0 | 0 | 0 | 0 |
|  | | Thaumatomonadida | Thaumatomonas seravini | | 0 | 1 | 0 | 0 | 0 | 0 | 0 | 0 | 0 | 0 | 0 | 0 |
|  | | unclassified Cercozoa | Cercozoa sp. DDB-2008c | | 2 | 1 | 0 | 0 | 0 | 0 | 0 | 0 | 0 | 0 | 0 | 0 |
|  | |  | Cercozoa sp. DDB-2008d | | 7 | 4 | 0 | 0 | 0 | 0 | 0 | 0 | 0 | 0 | 0 | 0 |
|  | |  | Cercozoa sp. DDB-2008e | | 1 | 0 | 0 | 0 | 0 | 0 | 0 | 0 | 0 | 0 | 0 | 0 |
| Rhodophyta | |  |  | |  |  |  |  |  |  |  |  |  |  |  |  |
|  | | Compsopogonales | Boldia erythrosiphon | | 3 | 1 | 0 | 0 | 0 | 0 | 0 | 0 | 0 | 0 | 0 | 0 |
|  | | Gigartinales | Mazzaella laminarioides | | 0 | 0 | 1 | 3 | 0 | 0 | 0 | 0 | 0 | 0 | 0 | 0 |
| stramenopiles | |  |  | |  |  |  |  |  |  |  |  |  |  |  |  |
|  | |  | Developayella elegans | | 0 | 0 | 0 | 0 | 0 | 0 | 3 | 3 | 0 | 0 | 0 | 0 |
|  | | Oomycetes | Peronosporaceae | | 0 | 0 | 0 | 0 | 0 | 1 | 0 | 0 | 0 | 0 | 0 | 0 |
|  | |  | Phytophthora drechsleri | | 0 | 0 | 0 | 0 | 0 | 0 | 3 | 2 | 0 | 0 | 0 | 0 |
|  | |  | Pythium aphanidermatum | | 0 | 0 | 0 | 0 | 0 | 1 | 0 | 0 | 0 | 0 | 0 | 0 |
|  | |  | Pythium monospermum | | 0 | 0 | 0 | 0 | 0 | 0 | 1 | 0 | 0 | 0 | 0 | 0 |
|  | | PX clade | Vaucheria litorea | | 0 | 1 | 0 | 0 | 0 | 0 | 0 | 0 | 0 | 0 | 0 | 0 |
| Viridiplantae | |  |  | |  |  |  |  |  |  |  |  |  |  |  |  |
|  | | Chlorophyta | Caulerpaceae sp. LM98 | | 1 | 1 | 0 | 0 | 0 | 0 | 0 | 0 | 0 | 0 | 0 | 0 |
|  | | Streptophyta | Acidodontium ramicola | | 0 | 0 | 0 | 0 | 0 | 0 | 0 | 0 | 0 | 0 | 1 | 0 |
|  | |  | Bryum argenteum | | 0 | 0 | 0 | 0 | 0 | 0 | 0 | 0 | 0 | 0 | 2 | 1 |
|  | |  | Bryum caespiticium | | 0 | 0 | 0 | 0 | 0 | 0 | 0 | 0 | 2 | 0 | 0 | 0 |
|  | |  | Bryum pachytheca | | 0 | 0 | 0 | 0 | 0 | 0 | 0 | 0 | 1 | 0 | 0 | 0 |
|  | |  | Mielichhoferia elongata | | 0 | 0 | 0 | 0 | 0 | 0 | 0 | 0 | 0 | 0 | 0 | 1 |
|  | |  | Pohlia cruda | | 0 | 0 | 0 | 0 | 0 | 0 | 0 | 0 | 0 | 1 | 0 | 0 |
|  | |  | Campylostelium saxicola | | 0 | 0 | 0 | 0 | 0 | 0 | 0 | 0 | 0 | 1 | 0 | 0 |
|  | |  | Streptopogon calymperes | | 0 | 0 | 0 | 0 | 0 | 0 | 0 | 0 | 0 | 0 | 0 | 2 |
|  | |  | Physcomitrella patens | | 0 | 0 | 0 | 0 | 0 | 0 | 0 | 0 | 0 | 0 | 0 | 1 |
|  | |  | Hypnum dieckei | | 0 | 0 | 0 | 0 | 0 | 0 | 0 | 0 | 1 | 0 | 0 | 0 |
|  | |  | Macrohymenium muelleri | | 0 | 0 | 0 | 0 | 0 | 0 | 0 | 0 | 0 | 0 | 0 | 1 |
|  | |  | Oxymitra incrassata | | 0 | 0 | 0 | 0 | 0 | 0 | 0 | 0 | 0 | 0 | 7 | 7 |
|  | |  | Riccia ciliifera | | 0 | 0 | 0 | 0 | 0 | 0 | 0 | 0 | 0 | 0 | 2 | 1 |
|  | |  | Riccia fluitans | | 0 | 0 | 0 | 0 | 0 | 0 | 0 | 0 | 0 | 0 | 1 | 0 |
|  | |  | Riccia huebeneriana | | 0 | 0 | 0 | 0 | 0 | 0 | 0 | 0 | 1 | 0 | 1 | 0 |
|  | |  | Riccia sp. Qiu 94094 | | 0 | 0 | 0 | 0 | 0 | 0 | 0 | 0 | 3 | 3 | 0 | 0 |
|  | |  | Riccardia | | 1 | 1 | 0 | 1 | 0 | 0 | 0 | 0 | 0 | 0 | 0 | 0 |
|  | |  | Equisetum arvense | | 0 | 0 | 0 | 0 | 0 | 0 | 0 | 0 | 6 | 3 | 0 | 0 |
|  | |  | Equisetum telmateia | | 0 | 0 | 0 | 0 | 0 | 0 | 0 | 0 | 0 | 0 | 1 | 0 |
|  | |  | Artemisia | | 0 | 0 | 0 | 0 | 0 | 0 | 0 | 0 | 4 | 4 | 3 | 3 |
|  | |  | Chrysanthemum | | 0 | 0 | 0 | 0 | 0 | 0 | 0 | 0 | 2 | 2 | 0 | 0 |
|  | |  | Athanasiinae | | 0 | 0 | 0 | 0 | 0 | 0 | 0 | 0 | 0 | 0 | 0 | 1 |
|  | |  | Ursinia sp. Balele 38 | | 0 | 0 | 0 | 0 | 0 | 0 | 0 | 0 | 0 | 0 | 1 | 0 |
|  | |  | Kirengeshoma palmata | | 0 | 0 | 0 | 0 | 0 | 0 | 0 | 0 | 0 | 0 | 0 | 1 |
|  | |  | Glechoma hederacea | | 1 | 0 | 0 | 0 | 0 | 0 | 1 | 0 | 0 | 0 | 0 | 0 |
|  | |  | Origanum | | 0 | 0 | 0 | 0 | 0 | 0 | 0 | 0 | 0 | 0 | 2 | 0 |
|  | |  | Salvia | | 0 | 0 | 0 | 0 | 0 | 0 | 0 | 0 | 1 | 3 | 0 | 0 |
|  | |  | Cressa truxillensis | | 0 | 0 | 0 | 0 | 0 | 0 | 0 | 0 | 0 | 1 | 0 | 0 |
|  | |  | Solanum dulcamara | | 0 | 0 | 0 | 0 | 0 | 0 | 0 | 0 | 0 | 0 | 1 | 0 |
|  | |  | Rhizophoraceae | | 0 | 0 | 0 | 0 | 0 | 0 | 0 | 0 | 0 | 0 | 0 | 1 |
|  | |  | Lunania | | 0 | 0 | 0 | 0 | 0 | 0 | 0 | 0 | 0 | 0 | 0 | 1 |
|  | |  | Populus | | 0 | 0 | 0 | 0 | 0 | 0 | 0 | 0 | 1 | 2 | 0 | 0 |
|  | |  | Salix exigua | | 0 | 0 | 0 | 0 | 0 | 0 | 0 | 0 | 0 | 0 | 10 | 10 |
|  | |  | Prunus persica | | 0 | 0 | 0 | 0 | 0 | 0 | 0 | 0 | 2 | 0 | 3 | 0 |
|  | |  | Cakile maritima | | 0 | 0 | 0 | 0 | 0 | 0 | 0 | 0 | 0 | 0 | 2 | 0 |
|  | |  | Acorus | | 0 | 0 | 0 | 0 | 0 | 0 | 0 | 0 | 10 | 17 | 17 | 20 |
|  | |  | Pontederia sagittata | | 0 | 0 | 0 | 0 | 0 | 0 | 0 | 0 | 1 | 0 | 0 | 0 |
|  | |  | Carex sp. SH-2010 | | 0 | 0 | 0 | 0 | 0 | 0 | 0 | 0 | 2 | 2 | 0 | 0 |
|  | |  | Oreobolopsis clementis | | 0 | 0 | 0 | 0 | 0 | 0 | 0 | 0 | 0 | 0 | 0 | 1 |
|  | |  | Juncus ensifolius | | 0 | 0 | 0 | 0 | 0 | 0 | 0 | 0 | 1 | 0 | 0 | 0 |
|  | |  | Juncus himalensis | | 0 | 0 | 0 | 0 | 0 | 0 | 0 | 0 | 1 | 0 | 0 | 0 |
|  | |  | Calamagrostis epigeios | | 4 | 5 | 4 | 3 | 1 | 1 | 2 | 0 | 0 | 0 | 0 | 0 |
|  | |  | Avena | | 0 | 0 | 0 | 0 | 0 | 0 | 0 | 0 | 2 | 0 | 0 | 0 |
|  | |  | Phalaris arundinacea | | 0 | 0 | 0 | 0 | 0 | 0 | 0 | 0 | 4 | 0 | 6 | 0 |
|  | |  | PACCAD clade | | 0 | 0 | 0 | 0 | 0 | 0 | 0 | 0 | 0 | 0 | 1 | 0 |
|  | |  | Typha | | 0 | 0 | 0 | 0 | 0 | 0 | 0 | 0 | 21 | 8 | 31 | 10 |
