# Supplementary material for: Ribosomal DNA and Plastid Markers Used to Sample Fungal and Plant Communities from Wetland Soils Reveals Complementary Biotas
Source: PLoS One. 2016 Jan 5;11(1):e0142759. doi: 10.1371/journal.pone.0142759 (PMC4712138; doi:10.1371/journal.pone.0142759)
Supplement: S4 Table — (DOCX) [file pone.0142759.s009.docx]

**S4 Table. The most frequent ITS, LSU, and rbcL categories summarized by MEGAN at the species level.**

| **MEGAN Node / Species** | **Number of OTUs (Site A, Site B)** | |
| --- | --- | --- |
|  | **ITS** | **LSU** |
| Platygloea disciformis (Fungi, Basidiomycota) | 46 (25, 21) | 0 (0, 0) |
| Glomus versiforme (Fungi, Glomeromycota) | 42 (21, 21) | 2 (1, 1) |
| Peziza badia (Fungi, Ascomycota) | 31 (14, 17) | 13 (6, 7) |
| Pterula echo (Fungi, Basidiomycota) | 30 (16, 14) | 6 (2, 4) |
| Cryptococcus tephrensis (Fungi, Basidiomycota) | 27 (12, 15) | 0 (0, 0) |
| Plectosphaerella cucumerina (Fungi, Ascomycota) | 25 (14, 11) | 0 (0, 0) |
| Itersonilia perplexans (Fungi, Basidiomycota) | 22 (9, 13) | 4 (1, 3) |
| Synchytrium endobioticum (Fungi, Chytridiomycota) | 20 (11, 9) | 0 (0, 0) |
| Bullera globispora (Fungi, Basidiomycota) | 20 (10, 10) | 0 (0, 0) |
| Mycosphaerella graminicola (Fungi, Ascomycota) | 19 (11, 8) | 3 (2, 1) |
| Gabarnaudia betae (Fungi, Ascomycota) | 19 (10, 9) | 4 (2, 2) |
| Typhula maritima (Fungi Basidiomycota) | 19 (8, 11) | 0 (0, 0) |
| Paraconiothyrium sporulosum (Fungi, Ascomycota) | 18 (8, 10) | 0 (0, 0) |
|  | **LSU** | **ITS** |
| Eocronartium muscicola (Fungi, Basidiomycota) | 29 (17, 12) | 0 (0, 0) |
| Lentaria albovinacea (Fungi, Basidiomycota) | 18 (8, 10) | 0 (0, 0) |
| Cryptococcus sp. CBS 681.93 (Fungi, Basidiomycota) | 16 (8, 8) | 0 (0, 0) |
| Peziza badia (Fungi, Ascomycota) | 13 (6, 7) | 31 (14, 17) |
| Helvella maculata (Fungi, Ascomycota) | 13 (8, 5) | 13 (7, 6) |
| Uroleptus gallina (Alveolata, Ciliophora) | 13 (6, 7) | 0 (0, 0) |
| Emericellopsis terricola (Fungi, Ascomycota) | 11 (5, 6) | 0 (0, 0) |
| Hypochniciellum subillaqueatum (Fungi, Basidiomycota) | 11 (4, 7) | 0 (0, 0) |
| Peziza badiofusca (Fungi, Ascomycota) | 10 (6, 4) | 0 (0, 0) |
| Coslenchus costatus (Metazoa, Nematoda) | 10 (5, 5) | 0 (0, 0) |
| Cyttaria hookeri (Fungi, Ascomycota) | 9 (4, 5) | 0 (0, 0) |
| Neobulgaria pura (Fungi, Ascomycota) | 8 (3, 5) | 3 (1, 2) |
| Pulvinula constellatio (Fungi, Ascomycota) | 8 (3, 5) | 3 (1, 2) |
| Cryptococcus gastricus (Fungi, Basidiomycota) | 7 (3, 4) | 1 (1, 0) |
| Tetracladium breve (Fungi, Ascomycota) | 7 (3, 4) | 0 (0, 0) |
| Helicodendron conglomeratum (Fungi, Ascomycota) | 7 (3, 4) | 0 (0, 0) |
| Auriculoscypha anacardiicola (Fungi, Basidiomycota) | 7 (4, 3) | 0 (0, 0) |
| Pterula echo (Fungi, Basidiomycota) | 6 (2, 4) | 30 (16, 14) |
| Chytridiomycota sp. Mori B3 (Fungi, Chytridiomycota) | 6 (2, 4) | 12 (4, 8) |
| Engelmanniella mobilis (Alveolata, Ciliophora) | 6 (2, 4) | 0 (0, 0) |
| Tromeropsis microtheca (Fungi, Ascomycota) | 6 (3, 3) | 0 (0, 0) |
| Sphaeronaemella fimicola (Fungi, Ascomycota) | 6 (3, 3) | 0 (0, 0) |
| Schizothecium vesticola (Fungi, Ascomycota) | 6 (2, 4) | 0 (0, 0) |
| Pterula epiphylla (Fungi, Basidiomycota) | 6 (1, 5) | 0 (0, 0) |
| Mortierella verticillata (Fungi, Basal fungal lineages) | 6 (3, 3) | 0 (0, 0) |
| Diversispora celata (Fungi, Glomeromycota) | 6 (3, 3) | 0 (0, 0) |
| Plectus aquatilis (Metazoa, Nematoda) | 6 (3, 3) | 0 (0, 0) |
| Developayella elegans (stramenopiles) | 6 (3, 3) | 0 (0, 0) |
|  | **rbcL** | |
| Typha (Viridiplantae, Streptophyta) | 70 (52, 18) | |
| Acorus (Viridiplantae, Streptophyta) | 64 (27, 37) | |
| Salix exigua (Viridiplantae, Streptophyta) | 20 (10, 10) | |
| Oxymitra incrassata (Viridiplantae, Streptophyta) | 14 (7, 7) | |
| Artemisia (Viridiplantae, Streptophyta) | 14 (7, 7) | |
| Phalaris arundinacea (Viridiplantae, Streptophyta) | 10 (10, 0) | |
| Equisetum arvense (Viridiplantae, Streptophyta) | 9 (6, 3) | |
| Riccia sp. Qiu 94094 (Viridiplantae, Streptophyta) | 6 (3, 3) | |
| Prunus persica (Viridiplantae, Streptophyta) | 5 (5, 0) | |
| Thioalkalivibrio sp. K90mix (Bacteria, Proteobacteria) | 4 (4, 0) | |
| Chrysanthemum (Viridiplantae, Streptophyta) | 4 (2, 2) | |
| Salvia (Viridiplantae, Streptophyta) | 4 (1, 3) | |
| Carex sp. SH-2010 (Viridiplantae, Streptophyta) | 4 (2, 2) | |
| Bryum argenteum (Viridiplantae, Streptophyta) | 3 (2, 1) | |
| Riccia ciliifera (Viridiplantae, Streptophyta) | 3 (2, 1) | |
| Populus (Viridiplantae, Streptophyta) | 3 (1, 2) | |
