# Supplementary material for: Ribosomal DNA and Plastid Markers Used to Sample Fungal and Plant Communities from Wetland Soils Reveals Complementary Biotas
Source: PLoS One. 2016 Jan 5;11(1):e0142759. doi: 10.1371/journal.pone.0142759 (PMC4712138; doi:10.1371/journal.pone.0142759)
Supplement: S1 Text — (DOCX) [file pone.0142759.s010.docx]

**Methods**

**Characterizing the ITS1/ITS2 portion of ITS reads**

Since the variable ITS1 and ITS2 transcribed spacers were targeted using primers that anneal to conserved sequences in the 3’ SSU rDNA and 5’ LSU rDNA regions, the ITS reads are composed of a mixture of conserved and variable sequence regions. We analyzed how well we were able to target the variable ITS1 and ITS2 regions using the Fungal ITS Extractor (Nilsson et al., 2010; S1 Fig). The Fungal ITS Extractor was able to identify the conserved rDNA genes and extract an ITS1 and/or ITS2 sequence from 90% of reads (S1a Fig). We plotted a frequency histogram of the read length distribution of the extracted ITS1 and ITS2 reads (S1b Fig). After removing the conserved rDNA genes flanking the ITS1/ITS2 variable regions, about half our dataset is less than 100 bp in length and likely to represent only partial ITS1/ITS2 sequences. Additionally, neither ITS1 nor ITS2 was successfully extracted from ~10% of our ITS sequences. It is possible that at least some of these sequences belong to taxonomic groups that have deviant ribosomal genes and are not well defined using existing hidden Markov models such as members of the early diverging fungal lineages. Thus, to retain the maximum sequence information available in our ITS dataset we used ‘unextracted’ sequences in all subsequent analyses.

**Choosing sequence similarity cutoffs for OTU clustering**

In most studies, OTU sequence similarity cutoffs are selected based on estimated levels of intra- or inter-specific variation such as 97% sequence similarity for the ITS region (such as Nilsson et al., 2008). However, since levels of variation can be different among taxonomic groups any level chosen is mainly for computational convenience. The effect of choosing a sequence similarity cutoff on the number of clustered OTUs is shown in S2a Fig. The percentage of OTUs was measured with respect to the total number of OTUs when clustered at 100% sequence similarity. As expected, the number of recovered OTUs increases in a nearly exponential manner as the sequence similarity criterion approaches 100%.

To pick a consistent cutoff for clustering sequences we plotted the percent increase in the number of clustered OTUs as the sequence similarity criterion increased from 90% - 100% (S2b Fig). We used an arbitrary 20% OTU increase to delimit the point where the number of OTUs begins to increase nearly exponentially as sequence similarity increases by 1% increments. Using this criterion, a 97% sequence similarity cutoff was chosen for the ITS and 5’ LSU datasets, and a 95% sequence similarity cutoff was chosen for 3’ LSU and rbcL datasets. The average sequence similarity within the resulting OTU clusters ranged from 99.3-99.4% for ITS, 99.0-99.2% for LSU, and 98.8-99.1% for rbcL. It is likely that OTUs defined this way both splits and lumps a number of taxonomic species, but they are used here to facilitate taxonomic classifications and statistical comparisons using semi-automated methods (Lindner et al., 2011; Gazis et al., 2011).

***Pterula echo*: positive classification or missing reference sequence?**

Neighbor joining analyses were conducted by aligning all the seed sequences classified by MEGAN as *Pterula echo* with the sequences of four of the most common top BLAST hits using MUSCLE 3.8.31 (Edgar, 2004). The alignment was manually adjusted to correct for reverse complemented sequences in Mesquite version 2.74 (Maddison and Maddison, 2010). Since 5’ and 3’ fragments only fully covered either the ITS1 or ITS2 regions, they were analyzed separately. Each region was automatically re-aligned using MUSCLE. PHYLIP version 3.69 was used to conduct neighbor joining analyses using the Kimura two parameter model (K2P) with default parameters (Kimura, 1980). The resulting trees were midpoint rooted. 1000 neighbor joining bootstrap replicates were conducted and values greater than 60% are shown at the nodes.

**Results**

***Pterula echo*: positive classification or missing reference sequence?**

*P. echo* was a frequently sampled fungal species in this study. This particular species was first described from Trinidad and belongs to a genus with a mostly tropical distribution (McLaughlin and McLaughlin, 1972; McLaughlin and McLaughlin, 1979). A neighbor joining analysis shows that seed sequences classified as *P. echo* do cluster near the *P. echo* reference sequence, but with a substantial amount of sequence divergence (S5 Fig). Although it is possible that this is the first record of *P. echo* from temperate wetland soils, this cannot be reliably confirmed without collecting the fruiting body itself. It is also possible that these sequences represent a closely related but distinct *Pterula* species without a reference ITS sequence in GenBank.

References

Edgar RC. MUSCLE: multiple sequence alignment with high accuracy and high throughput. Nucleic Acids Research 2004;32: 1792-1797.

Gazis R, Rehner S, Chaverri P. Species delimitation in fungal endophyte diversity studies and its implications in ecological and biogeographic inferences. Molecular Ecology 2011;20: 3001-3013.

Kimura M. A Simple Method for Estimating Evolutionary Rates of Base Substitutions Through Comparative Studies of Nucleotide Sequences. Journal of Molecular Evolution 1980;16: 111-120.

Lindner DL, Banik MT. Intragenomic variation in the ITS rDNA region obscures phylogenetic relationships and inflates estimates of operational taxonomic units in genus *Laetiporus*. Mycologia 2011;103: 731-740.

Maddison WP, Maddison DR. 2010. Mesquite: a modular system for evolutionary analysis. Version 2.73 <http://mesquiteproject.org>

McLaughlin DJ, McLaughlin EG. Pure culture studies of fruiting and sporulation in a clavarioid fungus, *Pterula* sp. Mycologia 1972;64: 599-608.

McLaughlin DJ, McLaughlin EG. A new species of *Pterula* (Aphyllophorales) with corticioid characteristics. Canadian Journal of Botany 1979;58: 1327-1333.

Nilsson RH, Kristiansson E, Ryberg M, Hallenberg N, Larsson K-H. Intraspecific ITS Variability in the Kingdom Fungi as Expressed in the International Sequence Databases and Its Implications for Molecular Species Identification. Evolutionary Bioinformatics 2008;4: 193-201.

Nilsson RH, Veldre V, Hartmann M, Unterseher M, Amend A, Bergsten J, et al. An open source software package for automated extraction of ITS1 and ITS2 from fungal ITS sequences for use in high-throughput community assays and molecular ecology. Fungal Ecology 2010;3: 284-287.
